# A 13.06 Ma widespread ignimbrite in the Pannonian Basin captured a snapshot of shallow marine to coastal environment in Central Paratethys

**DOI:** 10.1038/s41598-025-07002-9

**Published:** 2025-07-02

**Authors:** Dávid Karátson, Pierre Lahitte, Maxim Portnyagin, Márton Palotai, Sándor Józsa, Emő Márton, Emőke Tóth, Boglárka Erdei, Sébastien Nomade, Károly Németh, Levente Iván, Márton Krasznai, Fanni Vörös, Tamás Biró, Jean-Louis Paquette, Lilla Hably, János Hír, Péter Prakfalvi, János Kiss, Zoltán Pécskay, Daniel A. Frick, Mátyás Hencz

**Affiliations:** 1 Department of Physical Geography, Eötvös Loránd University, Budapest, Hungary; 2 Laboratoire GEOPS, CNRS, Université Paris-Saclay, Orsay, France; 3 GEOMAR Helmholtz Centre for Ocean Research, Kiel, Germany; 4 Geology and Laboratory Department, Supervisory Authority for Regulatory Affairs, Budapest, Hungary; 5 Department of Petrology and Geochemistry, Eötvös Loránd University, Budapest, Hungary; 6 Paleomagnetic Laboratory, Supervisory Authority for Regulatory Affairs, Budapest, Hungary; 7 Department of Paleontology, Eötvös Loránd University, Budapest, Hungary; 8 Hungarian Natural History Museum, Budapest, Hungary; 9 Laboratoire des Sciences du Climat et de l’Environnement (LSCE), CEA, Université de Versailles St-Quentin et Paris-Saclay, Gif-sur-Yvette, France; 10 MTA-EPSS FluidsByDepth Lendület Research Group, HUN-REN Institute of Earth Physics and Space Science, Sopron, Hungary; 11 Saudi Geological Survey, National Program of Earthquakes and Volcanoes, Jeddah, Saudi Arabia; 12 Volcanic Risk Solutions, Massey University, Palmerston North, New Zealand; 13 Laboratoire Magmas et Volcans, Université Clermont Auvergne, Clermont-Ferrand, France; 14 Natural History Museum, Pásztó, Hungary; 15 Department of Geological, Geophysical and Mining Data Store, Supervisory Authority for Regulatory Affairs, Budapest, Hungary; 16 Department of Mineral Raw Material Exploration and Geophysics, Supervisory Authority for Regulatory Affairs, Budapest, Hungary; 17 HUN-REN Institute for Nuclear Research, Debrecen, Hungary; 18 Institute of Geosciences, Christian-Albrechts-University Kiel, Ludewig-Meyn-Str. 10, 24118 Kiel, Germany

**Keywords:** Natural hazards, Solid Earth sciences

## Abstract

Voluminous Miocene silicic volcanism sourced mainly from the extensional Pannonian Basin played a major role in the evolution of the Central Paratethys. Here, we identify a widely distributed (> 3150 km^2^) member of the Upper Rhyolite Tuff in Hungary, called the Dobi Ignimbrite, with a precise sanidine/plagioclase ^40^Ar/^39^Ar age of 13.064 ± 0.065 Ma (~ Badenian/Sarmatian boundary in Central Paratethys chronology). It has distinctive glass geochemistry with wide compositional variations, which conforms with large-scale silicic explosive eruptions. In line with this, the calculated minimum volume (~ 200 km^3^) of the Dobi Ignimbrite is consistent with a high-end VEI 7 eruption, with possible ultradistal transport distance of over 300 km. Most of the pyroclastic succession, which erupted in two phases, was emplaced on land, as it contains leaves and tree trunks in the basal layer that we correlate with the Badenian/Sarmatian ‘volcanic floras’ of northern Hungary. At the same time, the ignimbrite has a strongly phreatomagmatic character, and, together with the presence of free-floating foraminifera, this suggest that the source vent was located in coastal waters of the Central Paratethys. These findings indicate either a late Badenian marine incursion prior to the eruption, or the shift of the eruption center toward the sea.

## Introduction

**Fig. 1:**
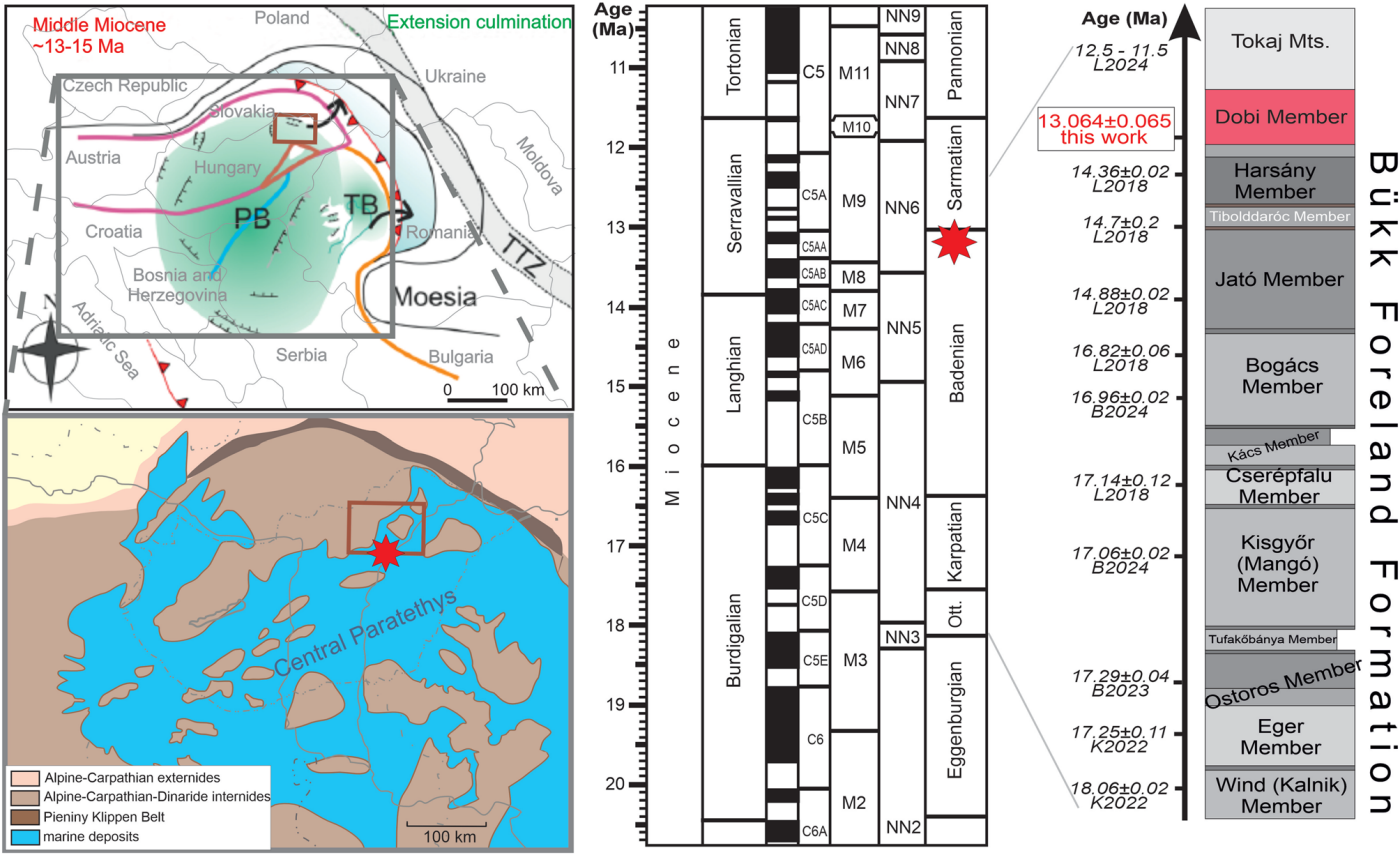
Tectonics, chronostratigraphy and paleogeography of the Dobi Ignimbrite. Left panel: Mid-Miocene (Late Badenian) plate tectonic setting (upper left map simplified after Balázs et al., 2016^7^, Free Access, ©2016 Americal Geophysical Union); green color marks the area of syn-rift subsidence. Gray inset refers to lower left map showing prevailing paleogeography of the vicinity of the study area (modified after Kovác et al.^18^, Open Access, https://geologicacarpathica.com/browse/archive/?journal_article_no=4943), which was an archipelagic sea of Central Paratethys. PB: Pannonian Basin, TB: Transylvanian Basin, TTZ: Trans-European Suture Zone. The small brown quadrangles mark the study area in Fig. 2; the red star indicates the hypothesized vent of the Dobi eruption. Middle panel: chronostratigraphy with Mediterranean and Central Paratethys stages, geomagnetic polarities, and biozones (after Kovác et al.^2^, Open Access, https://doi.org/10.1515/geoca-2018-0017). Right panel: members of the Bükk Foreland Volcanic Area volcanic formation (BFVA)^13^ showing the position of the subsequent Dobi eruption (member) and the even younger Tokaj Mts. volcanism^19^. Radiometric ages after Lukács et al.^12^ (L2018), Lukács et al.^19^ (L2024), Karátson et al.^20^ (K2022), Brlek et al.^21^ (B2023) and Brlek et al.^22^ (B2024).

The paleogeographic evolution of the Central Paratethys in Miocene times^1–4^ was coeval with extensional tectonics in the Pannonian Basin^5–8^, associated with large-scale ignimbrite volcanism^9–13^ (Fig. 1). This period was characterized by fluctuating transgressive and regressive periods^14,15^. However, deciphering the rapidly changing paleoenvironments and correlating the successive stages with the global time scale (GTS) is challenging due to the scarcity of well-defined marker horizons^2,16,17^.

Globally, one of the most promising ways of tracing paleogeographical changes is to identify and date massive volcanic eruptions that affected the surrounding environment^23–26^. The ignimbrite flare-up in the Pannonian Basin produced explosive events with far-reaching products, providing correlative tephras over Central Europe^12,20–22^. Moreover, tephras that preserve floral or faunal elements provide a characteristic snapshot of the paleogeography at the time of deposition.

Karátson et al.^20^ recently studied the 17.25 Ma Ipolytarnóc Fossil Site, famous for its rich flora and a sandstone footprint^27,28^ preserved by thick pyroclastics of a VEI ≥ 7 eruption. Building on this and other recent studies^13,28,29^, we highlight evidence suggesting that similar terrestrial landscapes dominated the northern Pannonian Basin for at least 5 million years during the emplacement of successive ignimbrites in the Bükk Foreland Volcanic Area (BFVA).

Here, we identify a 13.06 Ma large-magnitude eruption that followed the BFVA ignimbrites (18.06–14.36 Ma), producing—as we call it—the Dobi Ignimbrite. This eruption covered large areas in northern Hungary, with deposits still identifiable over >3000 km^2^. We suggest that this eruption affected even more distant regions of Central Europe and can serve as a significant marker horizon; and most importantly, we document that the source vent, for the first time after the emplacement of earlier ignimbrites, changed dramatically: the eruption took place in a submarine environment. Based on this evidence, we infer an onset of marine flooding of the epicontinental sea around the Badenian–Sarmatian boundary in the Central Paratethys from the east, or the shift of the eruption center seaward.

## Results

### Physical volcanology

The forested, hilly (< 1000 m), rugged terrain of the study area has been affected by neotectonic uplift, which resulted in significant erosion of ignimbrites outcropping in a highly scattered pattern.

**Fig. 2:**
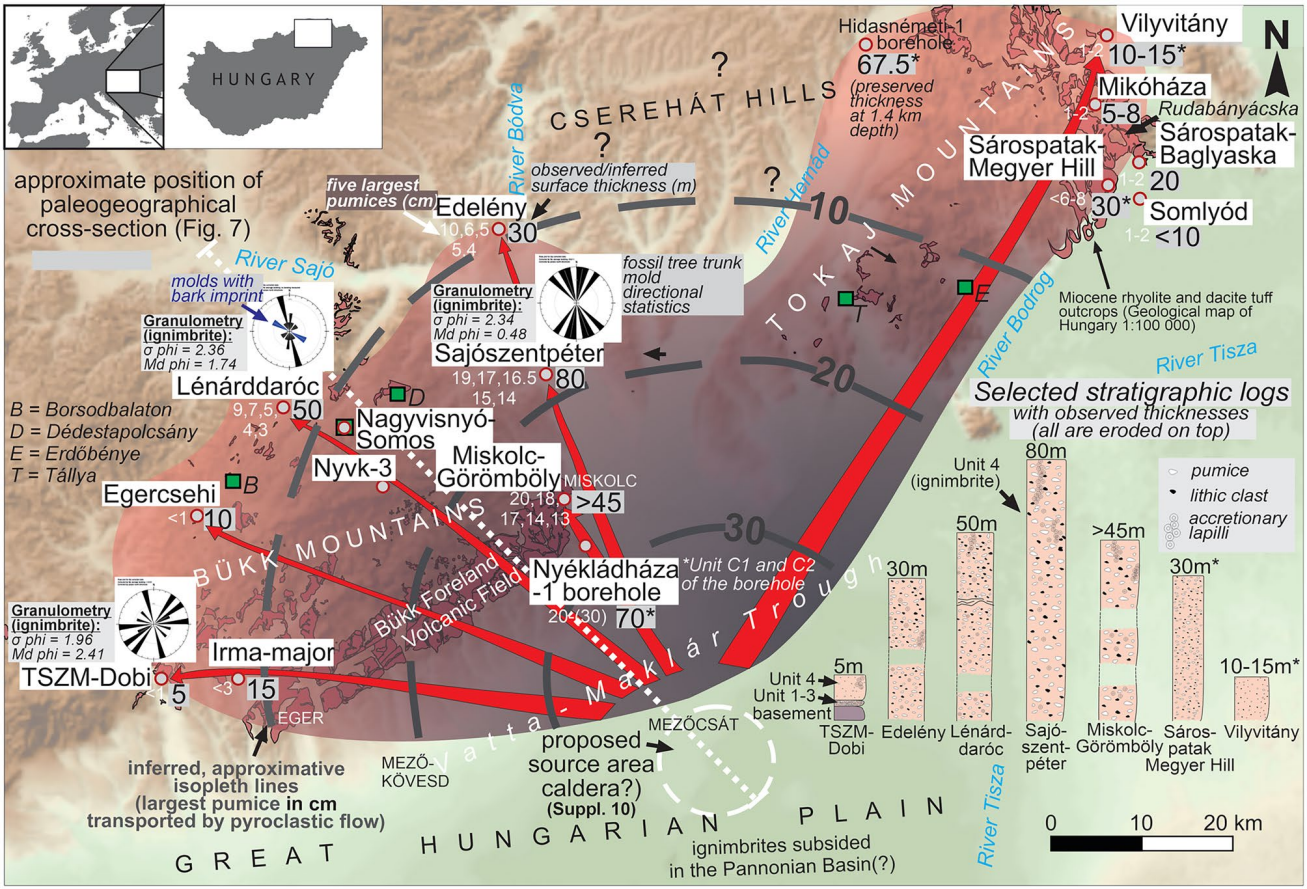
Study area showing the reconstructed areal distribution of the Dobi Ignimbrite observable today, displaying outcrops and boreholes (with maximum pumice size of Unit 4 ignimbrites in cm, also showing approximate isopleths); red arrows indicating assumed spread of pyroclastic flows; and directional statistics (black rose diagrams). Map is draped over a shaded relief image derived from the 50 m DEM of Hungary; source of surface occurrences from Mining and Geological Survey of Hungary MBFSZ 1:100,000 geological map (https://map.mbfsz.gov.hu/); image created with Surfer 13, version 13.0.383 Golden Software. Gray circles with red outline refer to study sites, green quadrangles to sites with known paleoflora (full names to the left). In the lower right corner, selected, simplified stratigraphic logs are shown (arranged from SW to NE). Data marked with * are taken from Lukács et al.^19^. White dashed line marks the rough position of the proposed paleogeographic cross-section in Fig. 7.

As a result of extensive fieldwork, we have correlated thirteen outcrops and two boreholes. Complemented by petrography and geochemistry (see below), these are proposed to belong to the Dobi Ignimbrite (Fig. 2; for volcanological description, see Supplement 1; for representative granulometry data, see Supplement 2). All but two sites (where fine tuffs with only a few dm thickness are found) reveal monotonous, massive, pumiceous, fine- to coarse-grained tuffs/lapilli tuffs interpreted as ignimbrites. The upper boundaries of the pyroclastics are eroded, while the lower contact is generally not exposed, except at one key site (TSZM-Dobi). However, quarry heights or hillslopes with stepwise outcrops allow an estimation of minimum uneroded ignimbrite thicknesses of up to 80 m (Fig. 2 and Supplement 1).

Within the ignimbrite, pumice clasts are subangular to slightly rounded, with sizes ranging from < 1 mm to 20 cm, and a systematic decrease from the vicinity of Miskolc town toward the west (Mátra Mts.), north (Cserehát Hills) and northeast (Tokaj Mts). This allows tentative isopleths to be established (Fig. 2 and Supplement 1). A detailed granulometry of three key sites shows decreasing clast size (Md phi) and slightly improved sorting (σ phi) of the ignimbrite with distance (Fig. 2 and Supplementary figure in Supplement 2). In accordance with these physical volcanological finds, we suggest a continuous facies change from medial (S) to distal (W, N and NW) occurrences (see Fig. 2)*.*

The basal sequence is exposed only at the Tarnaszentmária (TSZM-) Dobi site, in a cellar dug into the ignimbrite (Fig. 4). Here, a 5 m-thick pyroclastic sequence overlies a claystone-siltstone substrate capped by a 5–10 cm thick lithified paleosol. The first eruptive product with a sharp contact is a 1–2 cm, very fine tuff interpreted as an initial blast (Unit 1), followed by a ~15 cm coarse tuff, a distal pumice fall (Unit 2). The contact of the paleosol and the pyroclastics reveals leaf imprints and abundant stem and branch remains. The pumice-fall deposit is overlain by a ~5 cm thick tuff, a pyroclastic surge deposit (Unit 3) and finally a several m-thick, fine- to coarse-grained tuff, a distal pumiceous (≤ 1 cm grain size) pyroclastic-flow deposit (ignimbrite; Unit 4). The fine matrix of the ignimbrite contains glass shards (mostly cuspate-type) and subordinate sedimentary lithics (Supplement 4), and < 1 cm, evenly distributed, rim-type (ash-coated) accretionary lapilli, sometimes concentrated in gas escape structures. Charred tree branches and molds also appear randomly.

Near Lénárddaróc (Fig. 2), a hillside consists of Unit 4 ignimbrite exposed in a two-level quarry. Here, the ignimbrite is coarser-grained (lapilli tuff with cm-sized pumices), and in the lower quarry it is divided by an undulating, cross-bedded unit up to 0.5 m thick. This bed was described previously as a surge deposit^19,30,31^ but we interpret it as an epiclastic unit since it has the same granulometry as the ignimbrite, showing slight reworking.

Directional statistics were performed on the elongated tree molds within the ignimbrite (see Methods and Supplement 4). Figure 2 displays the tilt-corrected plunge directions of molds measured at TSZM-Dobi, Lénárddaróc and Sajószentpéter sites. At Sajószentpéter, mold orientations show a clear N-S trend. At Lénárddaróc and TSZM-Dobi sites, orientations indicate the preferred transport directions, i.e. NW–SE and W–E, respectively, although perpendicular and other orientations were also observed. At Lénárddaróc, a distinction can be made between the orientation of trunk molds showing bark imprint and that of trunk molds lacking bark (see Discussion).

### Petrography and geochemistry

Set in a matrix of glass shards, the mineral assemblage of the Dobi Ignimbrite (Supplement 4) consists of plagioclase, quartz, biotite and sanidine, in decreasing order, and a small quantity of zircon, apatite, and Fe–Ti oxides. Fresh pumice lapilli with tubular, vesicular structure, ranging from millimeters to (more rarely) centimeters in size, are frequent. Accessory lithics include characteristic claystone (or meta-claystone) often containing planktonic foraminifera such as globigerinids, as well as phyllite, muscovite, quartzite, calcite, micritic limestone, and andesite. Foraminifera also occur in the ignimbrite matrix (see Paleontology).

In addition to sites of the western and central part of the area covered by the Dobi Ignimbrite, thin section analyses of sanidine-bearing rhyolite tuffs of the Rudabányácska area (Fig. 2, Supplement 4: sites 12–14) in the Tokaj Mts.—dated earlier by the K–Ar method around 13 Ma^32,33^ (see Discussion)—suggest that these tuffs also belong to the Dobi Ignimbrite. Unfortunately, no fresh pumice glass was found in the samples due to their alteration (K-metasomatism: > 8 wt% K_2_O^33^); however, we can rely on the recent results of Lukács et al.^19^, who correlated the Lénárddaróc ignimbrite with the Sátoraljaújhely Unit (SAU) in the Tokaj Mts. based on similar zircon trace element composition and ~13.1 Ma zircon U–Pb ages (see Discussion).

**Fig. 3:**
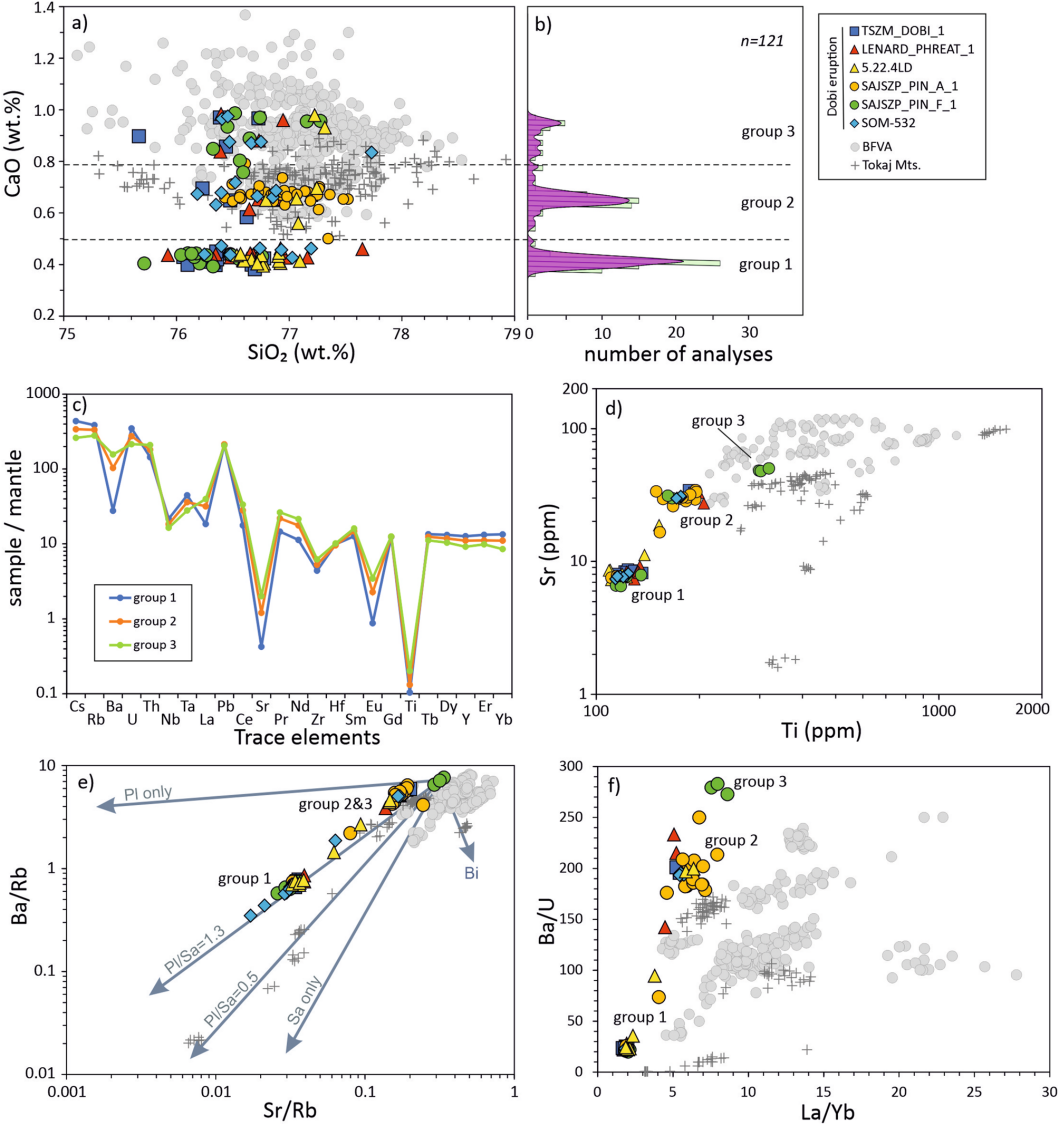
Major and trace element composition of volcanic glass from the Dobi Ignimbrite collected at TSZM-Dobi, Sajószentpéter, Lénárddaróc and Nagyvisnyó-Somos sites (including an analyzed sample of Lénárddaróc by Lukács et al. 2004^19^) in comparison with data from the 18.06–14.36 Ma ignimbrites of the Bükk Foreland Volcanic Area (BFVA; Hencz et al. 2024)^13^ and Tokaj Mts. ^19^ (**a**) Cao versus SiO_2_; (**b**) Histogram and Kernel density distribution of CaO content in three groups of the Dobi Ignimbrite glasses; (**c**) Spider diagram ofmantle-normalized trace elements in the three groups of glasses. Primitive mantle composition is after McDonough and Sun^101^; (**d**) Sr vs. Ti; (**e**) Sr/Rb vs. Ba/Rb illustrating crystal fractionation control on the composition of samples studied. Dobi glass can be explained by crystallisation of plagioclase and sanidine in proportion of ~ 1.3 in addition to quartz and biotite. Many Tokaj glasses require a larger amount of sanidine of up to  ~ 0.5. Sanidine is not a major phase in BFVA ignimbrites. Crystallization trajectories at different proportions of plagioclase (Pl) and sanidine (Sa) are after Lukacs et al.^19^; (**f**) La/Yb versus Ba/U. Dobi Ignimbrite glasses have the lowest Ba/U at a given La/Yb in comparison with glasses from the BFVA and Tokaj ignimbrites.

Chemical analyses of fresh glass from pumice samples were obtained from four sites (see Methods and Supplement 5A). The glass has a high-SiO_2_ (76–78 wt%) rhyolitic composition, and many of the major and trace elements overlap with compositions reported for ignimbrites of BFVA^13^ and the neighboring Tokaj Mts^19^. Despite these general similarities, Dobi glasses exhibit distinctive features (Fig. 3). Firstly, Dobi glasses exhibit trimodality in the content of CaO analyzed by electron probe (Fig. 3a, b). The most abundant glasses of Group 1 have CaO < 0.5 wt% (peak at 0.43 wt%), which differs from the BFVA and Tokaj ignimbrites. Less abundant glasses of Group 2 with CaO = 0.5–0.8 wt% (peak at 0.67 wt%) are similar to the prevailing BFVA and Tokaj glass compositions. The least abundant Group 3 glasses have CaO >0.8 wt% (peak at 0.97 wt%) and are the most similar to high-Ca BFVA glasses. Glasses belonging to all three groups were identified in every sample analyzed. Thus, the large variations of CaO in glasses are a very distinctive feature of the Dobi eruption. The presence of two or more populations of glasses is not a ubiquitous feature of silicic tephras but it has been documented for some large-volume (e.g. VEI = 7) explosive products in the Pannonian Basin such as Harsány Ignimbrite^34^ and Wind/Kalnik Ignimbrite^21^; moreover, there are examples from the more recent past such as the 39.8 ka Campanian Ignimbrite^35^ or the 74–75 ka Youngest Toba Tuff^36–38^.

The CaO content in Dobi glasses correlates positively with Ti, Sr, Ba, Th, Eu, light and middle REEs and negatively with Mn, Cs, U, Rb, Nb, Ta, Y and heavy REEs (Supplement 5B), so that the three groups of glasses have distinctive trace element patterns (Fig. 3c) and can be distinguished on paired diagrams of absolute contents of trace elements or their ratios (Fig. 3d, f)*.* As mentioned above, Lukács et al.^19^ correlated the Lénárddaróc ignimbrite with the Sátoraljaújhely Unit (SAU) in the Tokaj Mts. Indeed, the trend of Dobi glasses towards low Sr < 10 ppm, Ba < 200 ppm, Ba/Rb < 1 and Sr/Rb < 0.1 is similar to that observed for some Tokaj ignimbrites^19^ and reflects crystallization of K-feldspar from rhyolite melts (Fig. 3e). However, all Dobi glasses have significantly lower Ti at given Si and Ba contents (Fig. 3d), as well as lower La/Yb at a given Ba/U (Fig. 3f) compared to Tokaj glasses. On this basis, they cannot be unequivocally interpreted as deriving from either BFVA or Tokaj Mts.

### Volume of the ignimbrite

The inferred areal distribution of the Dobi Ignimbrite with 60–80 km runout (Fig. 2, Supplement 1) provides the basis for estimating its minimum bulk volume. However, even for modern ignimbrites, this calculation is subject to large uncertainties^40–43^. For the Dobi Ignimbrite, the original thicknesses are underestimated due to the significant erosion. This is proven by an outlier thickness data of 67.5 m in the Hidasnémeti-1 borehole^19^ (Fig. 2: see Discussion). Yet, starting from observed/inferred surface thickness data as minimum values (Supplement 1), they seem to follow a concentric pattern with smaller values toward distal areas (Supplementary Fig. 1 in Supplement 6).

For recent ignimbrites, the areal distribution of ignimbrites was considered either using an average thickness value^44–46^ or a maximum thickness at the center and zero volume at the perimeter^40,46^. Using the observed/inferred surface thicknesses of the Dobi Ignimbrite, we constructed apparent isopach lines and applied a geometric approximation (Supplementary Fig. 2 in Supplement 6). This approach yields a minimum estimate of 170 km^3^ and a more realistic value of ~200 km^3^. A theoretical maximum volume has also been calculated (370 km^3^).

### Paleontology

Micropaleontological studies were carried out on the underlying strata and the ignimbrite from the TSZM-Dobi and Lénárddaróc sites. Only siliceous spicules of *Demospongea* sponges were extracted from the prevolcanic claystone which can be classified in the suborder *Astrophorina* and the family *Geodiidae.* Today, these demosponge sponges are found worldwide in normal marine environments, with a wide depth distribution from shallow to bathyal regions^47–49^. For the Pannonian Basin, Hajós^50^ described geodiid sterraster-type sponge spicules from Badenian open marine sediments, which are also proposed for the underlying succession of the Dobi Ignimbrite.

**Fig. 4:**
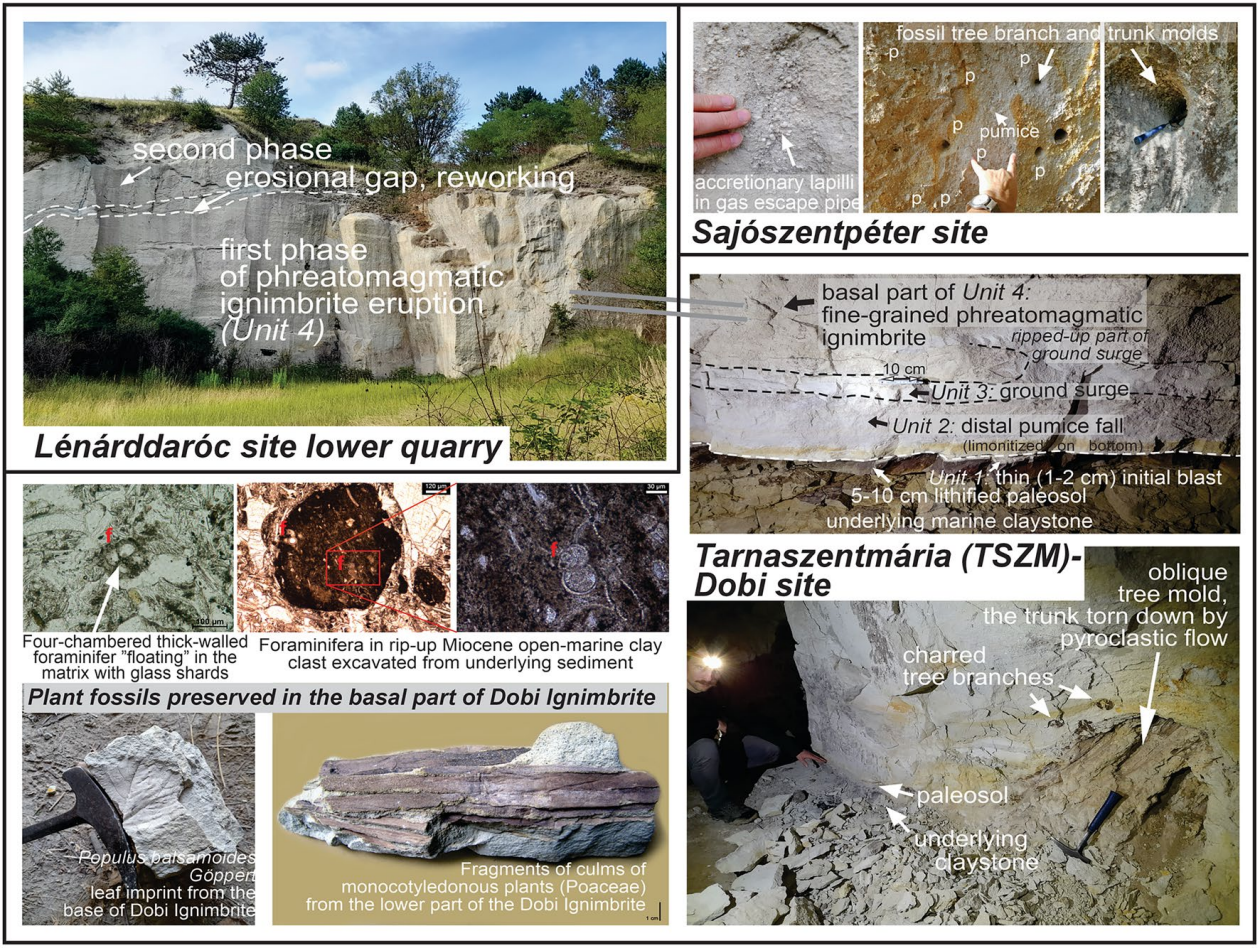
Photo compilation of main features of the Dobi Ignimbrite. Upper left photo and right panels: field relationships of the eruptive sequence from three key sites. On the middle left photos, typical foraminifera (f), while on the lower left photos, plant imprints/fossils from the TSZM-Dobi site are shown (inventory numbers for the plant fossils: HNHM-PBO 2024.471.1.–483.1).

Rare foraminifera, which are absent in BFVA ignimbrites^13^, were identified in ignimbrite thin sections. Foraminifera occur in two types of preservation. First, the consolidated claystone lithics in the matrix (Supplement 4) contain only small-sized, thin-walled Miocene planktonic foraminifera (Fig. 4) which indicates an open-marine environment representing the prevolcanic sedimentary succession. We suggest that these clasts were excavated from the underlying substrate during the Dobi eruption. Second, the ignimbrite matrix also contains some free-floating, isolated benthic and thick-walled planktonic foraminifera. They also refer to a marine environment; the planktonic forms might have drifted into coastal waters of the inland sea where they were ripped up by the eruption (see Discussion).

By contrast, plant fossils found at and above the lower contact of the Dobi Ignimbrite (see Fig. 4) indicate terrestrial emplacement. They can be divided into (1) imprints or molds of stems, tree branches, twigs and leaves, and (2) charred tree trunks and branches (Fig. 4). Among the leaf imprints, including damaged, unidentifiable leaf fragments of dicotyledonous plants, some have been assigned to the species *Populus balsamoides* Göppert which is considered an element of wetland vegetation during the Miocene. In the Central Paratethys, this species appeared in the Karpatian^51^ and became dominant in some Badenian flora and, in the northern Pannonian Basin, especially in Sarmatian flora^52^, together with other poplar species. Among the impressions and molds of stems, twigs and subordinate leaves, monocotyledon plants are dominant, identified as remains of *Poaceae (?Bambusoideae),* which corroborate the presence of wetland vegetation.

### U–Pb and Ar–Ar dating

Two dating techniques were applied to determine the precise eruption age. U–Pb dating^53^ on the TSZM-Dobi site (see Methods and Supplement 7) was followed by ^40^Ar/^39^Ar dating^54^ on TSZM-Dobi and Lénárddaróc sites (Methods and Supplement 8A, 8B), previously correlated by geochemistry. All errors are reported at 2σ.

Zircon ^206^Pb/^238^U dating yielded an age of 13.202 ± 0.153 Ma (2σ), MSWD = 0.55, n = 33**.**

**Fig. 5:**
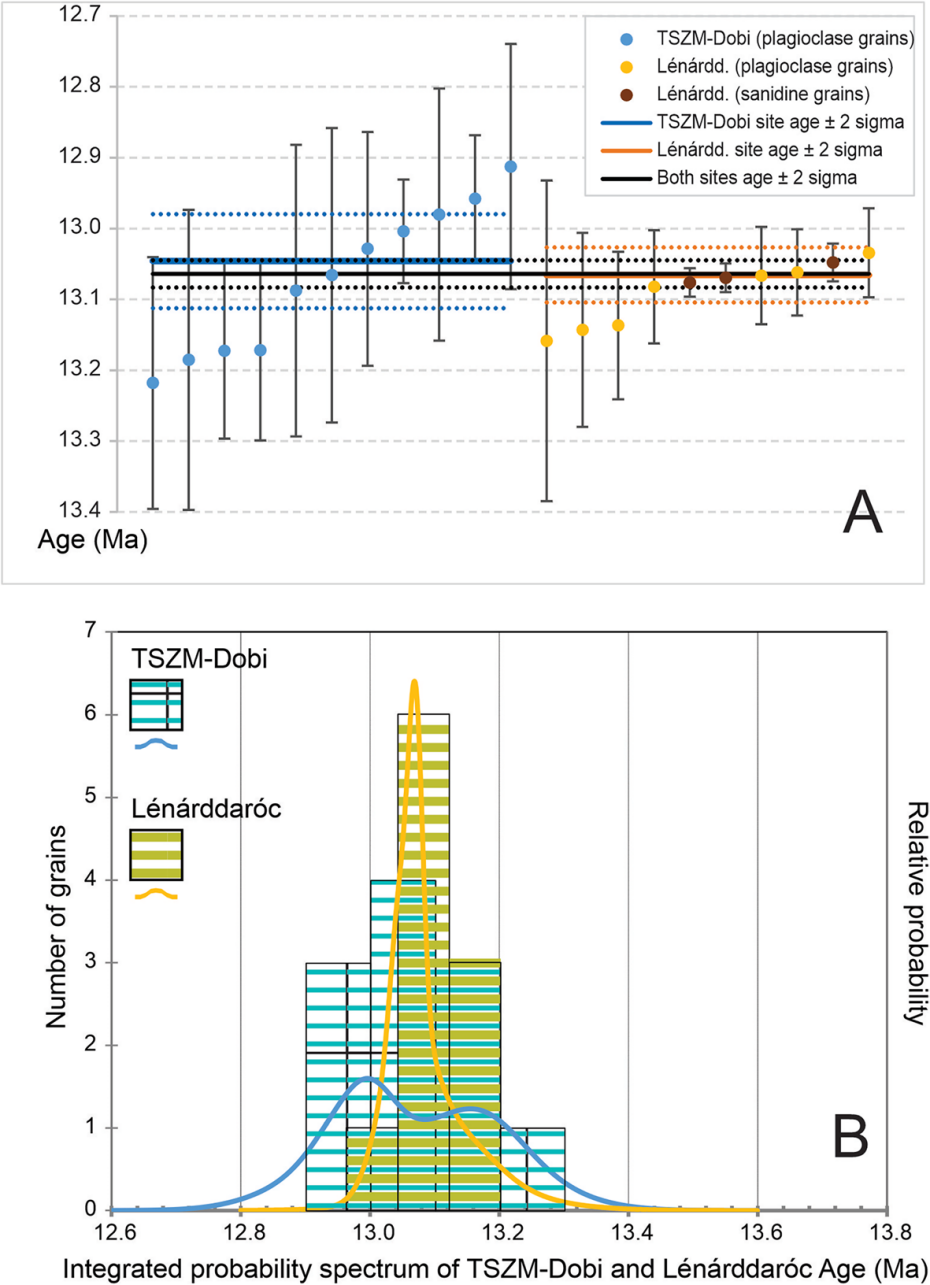
Ar–Ar geochronology of the Dobi Ignimbrite. Upper plot: weighted mean ages for the 11 and 10 dated grains, respectively, from the TSZM-Dobi and Lénárddaróc site, showing 2 σ error bars. Lower plot: probability spectrum of both datings.

Results of sanidine and plagioclase ^40^Ar/^39^Ar dating show individual single crystal ages and corresponding probability diagrams (Fig. 5A, B). For TSZM-Dobi, the dating of eleven crystals yielded a weighted mean age of 13.046 ± 0.091 Ma including external uncertainties (MSWD = 2.31, p = 0.021) with individual ages ranging from 12.91 ± 0.17 to 13.22 ± 0.18 Ma. For Lénárddaróc, a more precise 13.066 ± 0.073 Ma age (MSWD = 0.84, p = 0.58) was obtained: the probability spectrum, based on ten dated crystals, is less scattered with individual ^40^Ar/^39^Ar ages ranging from 13.03 ± 0.06 to 13.16 ± 0.23 Ma. The higher precision is due to the presence of three sanidines among the dated grains, with analytical uncertainties five times better than for plagioclases. Importantly, ages obtained from the three sanidine grains alone (13.069 ± 0.031 Ma) and those from the seven plagioclase grains (13.065 ± 0.033 Ma) are indistinguishable, which supports the coeval crystallization of these minerals.

From the analysis of twenty-one grains, taking into account that both sites give compatible ages, the proposed eruption age is 13.064 ± 0.019 Ma (for comparison with ^40^Ar/^39^Ar ages), and 13.064 ± 0.065 Ma (including all external errors for comparison with other dating methods; MSWD = 1.4, p = 0.25).

### Geophysics

The Dobi Ignimbrite occurrences were drilled from the W–NW (sites 1–3) and NE (sites 4 and 5) margins of the Bükk Mts. for paleomagnetic investigation^39,55,56^ (see Methods and Supplement 9). From a total of 40 cores, 35 samples yielded positive results^57^.

**Fig. 6:**
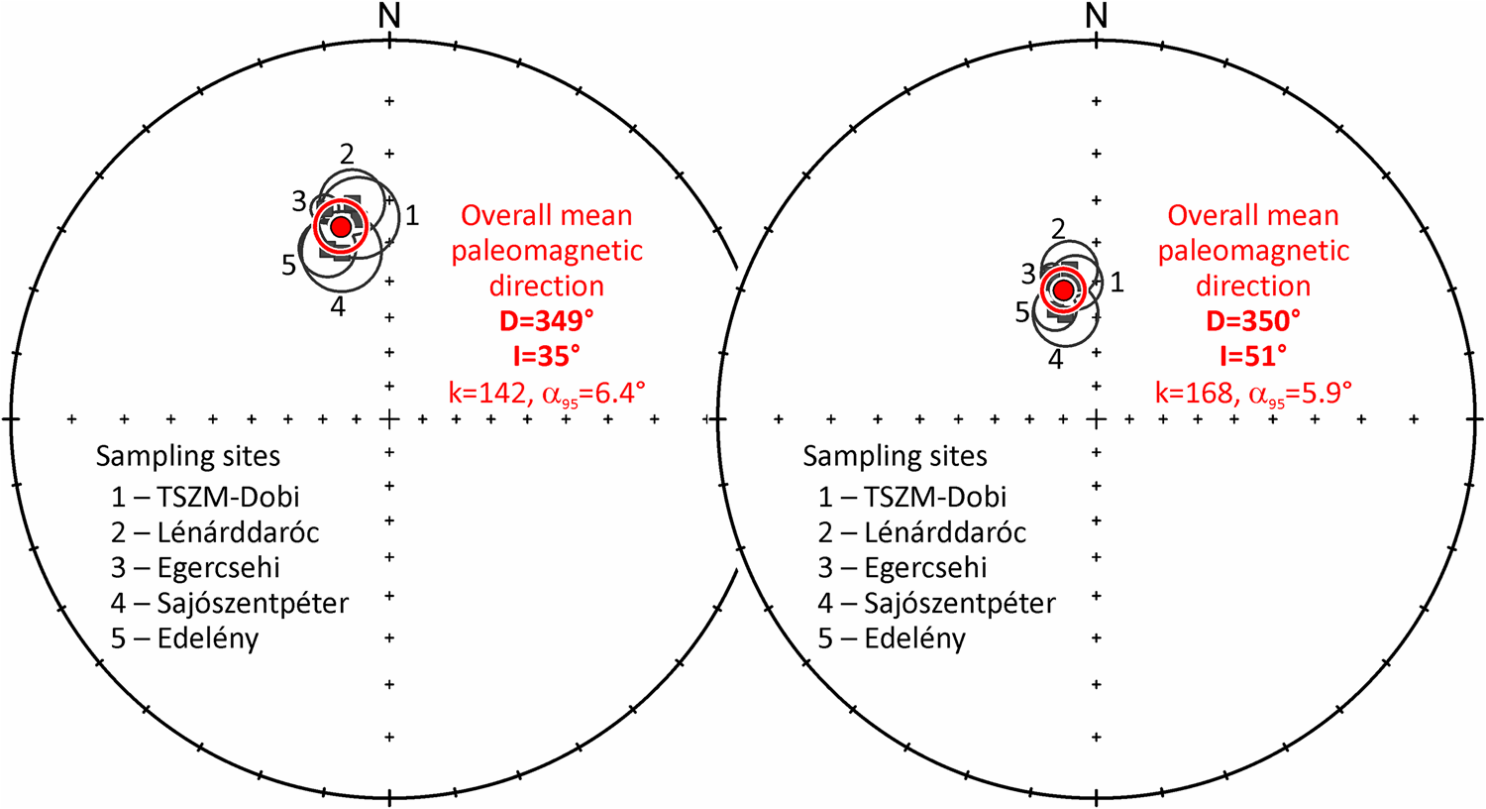
Paleomagnetic correlation of Dobi Ignimbrite exposures. Paleomagnetic directions of five sites are shown with confidence circles before (left) and after (right) tilt and flattening corrections. Full red circle marks the overall mean paleomagnetic direction.

Statistical evaluation on site^58^ resulted in well-defined northerly paleomagnetic directions (Fig. 6 left), all of them with normal polarity and shallow inclination. The shallow inclinations, attributed to compaction, were corrected using the elongation-inclination (E/I) method^59^ (Fig. 6 right). The corrected overall-mean paleomagnetic direction, in line with Ar–Ar dating, confirms that the Dobi Ignimbrite was deposited after the termination of the Miocene CCW rotation of the larger area^55^.

Bouguer anomaly and ΔZ magnetic anomaly maps of NE Hungary were also compiled to constrain the source area (see Methods and Supplement 10). Possible source vents (calderas) have long been envisaged along the neotectonic Vatta-Maklár Trough^13,29,60–62^ (Fig. 2): e.g. a Bouguer anomaly minimum zone SE of Eger town was linked to the proposed center of the Eger-Ipolytarnóc eruption^20^. A further 10 km to the SE, we outline another minimum zone (Supplementary figure in Supplement 10) whose position can be tentatively correlated with the source area of the Dobi eruption*.*

## Discussion

### The volcanic eruption

By correlating mosaic-like ignimbrite occurrences over an area of >3000 km^2^ in the northern Pannonian Basin, we identify a powerful silicic explosive eruption called the Dobi eruption. It is characterized by a specific, uniform chemistry and younger age relative to the known pyroclastic members of the nearby BFVA^13^. The trimodality of its glass composition implies a large-volume eruption with the involvement of separate magma batches, while the rarity of intermediate compositions between the three groups is in line with limited magma mixing. The major and trace element systematics of the glass compositions suggest that the three groups of glasses may be cogenetic and related to different extents of crystal fractionation (Fig. 3e), although they evolved spatially separate from each other. However, additional isotope analyses and mass-balance calculations are required to fully justify this conclusion.

During the eruption, magmatic and phreatomagmatic fragmentation are both suggested to have taken place^63–65^, as shown, in addition to the large amount of pumice, by the fine tuff matrix along the whole sequence up to 60–80 m thick, and the evenly distributed rim-type accretionary lapilli^66^. As these components are unchanged vertically at any location, we infer that magma-water contact was continuous during the eruption (except for the unknown, eroded topmost part). For the BFVA, the presence of phreatoplinian eruptions was already documented^29^ but without a clear source of water. Here, the available water was undoubtedly seawater, as evidenced by isolated, ripped-up foraminifera, and typical claystone clasts indicative of a marine substrate (i.e. excavated as accessory lithics from the underlying beds). In addition, subaerially, a portion of accretionary lapilli may have been generated in medial to distal settings by the occurrence of subtropical rain.

For similar submarine eruptions, foraminifera were reported from e.g. the recently discovered 510 ka Archaeos Tuff, Santorini^64^, Greece. However, in contrast to the Archaeos Tuff where most of the succession was deposited on the seafloor, the Dobi eruption must have occurred at shallower depths, as all deposits observable today were emplaced on land, and the basal contact at the TSZM-Dobi site exposes the fine details of a terrestrial environment. We propose that the high energy of the eruption, probably ensured by the intense gas flux^67^, was capable of generating a significant subaerial plume that emplaced a thick succession toward the coastal area (while submarine PDCs and related deposits cannot be excluded either).

After the initial powerful blast that blanketed the landscape, the successive phases of the eruption consisted of a typical Plinian sequence with pumice fall and a collapsing column that produced PDCs (surge deposit and much thicker ignimbrite). While the basal succession is exposed only in the Dobi cellar, field observations at Lénárddaróc on an intercalated epiclastic bed within the ignimbrite suggest there was possibly a time interval separating two phases of column collapse. Such an interval is also indicated in the Nyékládháza-1 borehole, where both the C1 and C2 ignimbrite units^68^ start with a thin blast, followed by a 20–30 cm-thick pumice fallout.

No data for the proximal setting were found based on the facies characteristics observed in the field. With regard to the inferred marine origin and the proposed southerly source vent of the Dobi eruption (see below), the proximal areas may have been buried beneath the Pannonian basin since the Miocene subsidence.

### Areal distribution, source region and magnitude of the eruption

The magnitude of an eruption in terms of the Volcanic Explosion Index (VEI) scale^69^ can be constrained by the area covered and the volume inferred. Despite the scattered outcrops, the Dobi Ignimbrite obviously blanketed the landscape over a vast area. The current large extent observable at the surface is also explained by the fact that the 18.06–14.36 Ma BFVA eruptions generally covered the older eruptive products, while after the Dobi eruption the silicic explosive volcanism of the Pannonian Basin shifted eastward to the Tokaj Mts.^19,32,70,71^.

Covering an area of over ~3150 km^2^, the Dobi Ignimbrite has already been identified in varying contexts. On the 1:100,000 scale geological map of Hungary^72^, the occurrences correlated herein with the Dobi Ignimbrite belong to the Harsány Rhyolite Tuff, which was dated to 14.36 Ma^12^, and recently classified as a single formation^73^. This general information may have led to the much younger Dobi Ignimbrite being under-interpreted. At the same time, within the Harsány Rhyolite Tuff, several occurrences containing accretionary lapilli, plant imprints and marine fossils were described (e.g. the “Kőkötőhegy” and “Bábaszék” members ^70,72^), with some of them likely assigned to the Dobi Ignimbrite.

Lukács et al.^19^, correlating the Lénárddaróc site with their SAU unit in the Tokaj Mts., proposed the unit to be made up of a 300–600 m-thick succession of various pyroclastics (massive and stratified) of rhyolitic to dacitic composition, intercalated with Badenian sediments. They claimed that the “Sátoraljaújhely eruption”—producing pyroclastic flows with possibly >100 km runout—was sourced from the area of Tokaj Mts, beginning the volcanism here. However, this suggestion was only based on the assumed, extraordinary thickness of the SAU unit total, without supporting radiometric or geochemical data. In this work, apart from the well-documented Tokaj Mts. surface exposures (sites 11–15), only the 67.5 m-thick ignimbrite in the Hidasnémeti-1 borehole, dated at 13.1 Ma by zircon U-Pb^19^, is considered to belong to the Dobi eruption (as part of the SAU succession), and even this thickness could be interpreted as local thickening of a valley-ponded ignimbrite.

Moreover, we see no justification for a source of Dobi Ignimbrite in the Tokaj Mts. for two reasons:

1. The unambiguous decrease of maximum pumice clast size from ~20 to 1–2 cm is traceable from the vicinity of Miskolc (sites 9, 10) towards either the western foothills of the Bükk Mts. (sites 1–3) or the Tokaj Mts. (sites 11–15; Fig. 2, Supplement 1). Regarding such a pattern, we refer to the basic findings by Giordano and Doronzo^74^ and Palladino and Giordano^75^, who distinguished two end-member ignimbrite types, one being characteristic of large-volume eruptions and flat topography. Ideally, this type of pyroclastic flow spreads out radially on an open surface (with negligible topographic control), and thickness and maximum lithic and pumice sizes show a mild, linear decrease with distance. For the Dobi Ignimbrite, although the proximal facies is unknown, the trend is obvious from medial toward distal settings (see Fig. 2).
2. Directional fabrics of torn-down trees transported by PDCs are commonly reported parallel-to-flow^76,77^, although individual directions can also be influenced by log size and transport distance. While our statistics (Fig. 2 and Supplement 3) are far from being comprehensive, the resultant directions closest (Sajószentpéter) to the assumed vent area fit a transport direction from the S/SE. At Lénárddaróc, tree molds with bark imprint are also parallel to the assumed flow (SE–NW: Fig. 2). These trunks may have been living trees torn down and carried away by the PDC^77^. In contrast, the molds lacking a bark imprint may represent previously fallen, dead trunks that were ripped up closer to the site of observation and are therefore less oriented. Moreover, the hypothesized source vent > 30 km south of Miskolc also seems to be supported by the Bouguer anomaly assessment, as presented above.

Assuming a flat terrain and a symmetrical spread of the Dobi Ignimbrite (northward of the Vatta-Maklár Trough), the original distribution and volume may have been larger than our estimated ~170–200 km^3^. There were presumably also significant intracaldera volumes, which cannot be seen due to basin subsidence. With all these restrictions, our bulk volume estimate is worth comparing to similar-sized examples worldwide. The Campanian Ignimbrite covers almost the same area and has the same maximum thickness (3215 km^2^ and 70–80 m, respectively; Supplement 6). Its calculated bulk volume is 457–660 km^3^, but half to two-thirds of this value is accounted for by co-ignimbrite ash which is unknown for the Dobi Ignimbrite. For the Dobi eruption, one candidate of ultradistal products might be the Babczyn Tuff recovered from a borehole in the Polish Carpathian Foredeep. Composed of glass shards, pumice, feldspar and biotite, it was dated at 13.06 ± 0.11 Ma (volcanic glass and biotite ^40^Ar/^39^Ar)^78^. Furthermore, its location over more than 300 km from Miskolc to the NE is in line with airborne fallout from a southerly transport direction.

### Age of the eruption

The first K–Ar dates for the youngest ignimbrites we assign to the Dobi Ignimbrite, including the sanidine-bearing rhyolite tuffs in the Tokaj Mts., e.g. Somlyód Hill (Fig. 2), were performed in 1986^32^, and yielded an age of 13.1 ± 0.5 Ma (Table 1). However, at that time, this age was not considered significant for understanding the silicic explosive volcanism of the northern Pannonian Basin, largely due to the formerly established young age of the Tokaj volcanism, and the absence of correlations with the western pyroclastic occurrences.

**Table 1:** Radiometric dating results (this work) and comparison with previously published dates of rhyolite tuffs in the northern Pannonian Basin assigned to the Dobi Ignimbrite (sites from SW to NE). *youngest zircon age, **Bayesian eruption age estimate.

Locality	Age (Ma)	External error (± 2σ)	Dating technique	Reference (see main text)
Tarnaszentmária (TSZM)-Dobi	13.046	0.091	Ar–Ar plagioclase	This work
	13.202	0.153	U–Pb zircon LA-ICP-MS	This work
Lénárddaróc	13.066	0.073	Ar–Ar sanidine, plagioclase	This work
	13.05	0.20	U–Pb zircon LA-ICP-MS	Lukács et al.^19^
Average of TSZM-Dobi + Lénárddaróc (proposed eruption age)	13.064	0.065	Ar–Ar sanidine, plagioclase	this work
Nyékládháza Ny-1 borehole 183.3 m	12.18	1.40	K–Ar biotite	Lukács et al.^68^
228.4 m	12.96	0.75	K–Ar biotite	Lukács et al.^68^
Sátoraljaújhely Baglyaska	13.2	0.8	K–Ar biotite	Pécskay et al.^32^
Somlyód Hill	13.1	0.5	K–Ar sanidine	Pécskay et al.^32^
Sátoraljaújhely Kopaszka Hill	13.3	0.5	K–Ar biotite	Pécskay and Molnár^33^
Mikóháza MIK-1	13.17	0.21	U–Pb zircon LA-ICP-MS	Lukács et al.^19^
	13.103*	0.024	U–Pb zircon CA-ID-TIMS	Lukács et al.^19^
	13.092**	-0.044 + 0.025	U–Pb zircon CA-ID-TIMS	Lukács et al.^19^
Sátoraljaújhely SAU-1	13.27	0.21	U–Pb zircon LA-ICP-MS	Lukács et al.^19^
Sárospatak Megyer Hill SP-MH-1	13.20	0.20	U–Pb zircon LA-ICP-MS	Lukács et al.^19^
Hn1_1483.7–1405.5 m borehole	13.11	0.20	U–Pb zircon LA-ICP-MS	Lukács et al.^19^

As presented above, a recent study^19^, presenting high-precision U–Pb zircon dates of the Tokaj Mts., correlated the oldest occurrences with the Lénárddaróc site, also showing ages of around 13.1 Ma. Commonly, zircon U–Pb ages tend to be older than the eruption ages (up to 0.1–0.5 My) due to magma residence time issues^79–81^. Indeed, our proposed ^40^Ar/^39^Ar sanidine and plagioclase eruption age of the Dobi Ignimbrite (13.064 ± 0.065 Ma), similar to the 13.06 ± 0.11 Ma age of the Babczyn Tuff, is slightly younger than most of the U–Pb zircon ages. At the same time, this age is indistinguishable within analytical uncertainty from the modeled zircon CA-ID-TIMS U–Pb age (13.092 ± 0.043) for the oldest Tokaj Mts. unit recently proposed^19^. More significantly, field volcanology data as well as the geochemical correlations presented in this study and by Lukács et al.^19^ have enabled the linkage of numerous previously undated or roughly dated sites thereby delineating the extensive areal distribution of the Dobi Ignimbrite.

### Paleoenvironmental implications

The free-floating foraminifera isolated in the ignimbrite matrix point to a marine environment of the source vent of the eruption. By contrast, the main depositional environment is likely to have been along the flat, but complex, coastal areas where shallow marine to terrestrial wetland environments changed within short distances. In a broader context, the paleogeography of the Central Paratethys underwent frequent, successive changes during Miocene times. In particular, between 15 and 12 Ma, the salinity conditions changed from brackish to hypersaline (Fig. 1), reaching a peak during the Badenian Salinity Crisis (BSC) probably triggered by a global sea-level drop^82^. The extended period of BSC (c. 13.8–13.3 Ma^83^) terminated when the Central Paratethys returned to open-marine conditions caused by sea-level rise in the Eastern Paratethys^18,84,85^. The new transgressive event culminated in a seawater incursion into the semi-open basin system^86^.

The BSC was followed by the Badenian-Sarmatian Extinction Event (BSEE)^87,88^, which defines the Badenian/Sarmatian stage boundary^87^. However, its timing is controversial (from 13.3 to 12.6 Ma^89^). As Kováč et al.^2^ emphasized, the BSEE might have been diachronous due to the complex tectonic evolution of the Carpathian–Pannonian region, and point-based data are required to trace the paleoenvironmental changes.

During the BSEE, the stenohaline groups disappeared, and the faunal diversity decreased markedly^90^. The presence of free-floating stenohaline planktonic foraminifera in the Dobi Ignimbrite suggests that the eruption preceded the extinction. In conclusion, the normal marine conditions in the northern Pannonian Basin may have existed by at least 13.06 Ma.

At the same time, the emplacement of the Dobi eruption captured a sharp paleoenvironmental boundary. The basal layers of the TSZM-Dobi site document a forested, presumably wetland environment. In addition, another tuff occurrence at the NW foothills of Bükk Mts.—referred to as the Nagyvisnyó–Somos valley by its first collector, I. Gaál (1949: Fig. 2)—also reveals a limited flora. The “Dobi–Somos” flora, preserved by the 13.06 Ma eruption, may be the first among the Badenian–Sarmatian volcanic floras in the North Pannonian region^52,91^, probably giving rise to the development of other known volcanic floras in the larger region (Balaton-Dellő, Dédestapolcsány, Bánhorváti, Erdőbénye and Tállya^52,92,93^: Fig. 2). Overall, the repetitive Miocene eruptions provide insights into the development of the vegetation in the region, from the evergreen, strongly thermophilous Early Miocene flora of Ipolytarnóc^20,27^, through the less thermophilous floras of the Badenian Nógrádszakál^94^, to the late Middle Miocene volcanic floras of the Bükk–Tokaj region, which are precisely dated here for the first time.

Finally, we claim that the areal distribution of the Dobi Ignimbrite can contribute to the understanding of the recent uplift history of the Bükk Mts. After a long record of repetitive subsidence and uplift terminated by a poorly dated Mid-Miocene exhumation, the final rise of these mountains started only around 2–3 Ma based on fission-track dating^95^. Although the concept of a Miocene, subaerial tuff cover over the Bükk Mts. prior to the uplift is widely accepted^96,97^, the specific ignimbrites have not been identified. In our study, the Nvk-3 shallow borehole (site 6) yielded a reworked tuffaceous sample from a karstic sinkhole on the Bükk plateau (Supplement 4) with a mineral assemblage of quartz, plagioclase and sanidine, i.e. supporting a Dobi Ignimbrite origin. Although the Harsány Ignimbrite at BFVA (14.36 Ma)^12^ also has the same minerals, no foraminifera were reported from the latter, while earlier studies documented foraminifera from the tuffaceous material ^97,98^ covering the karstified Bükk plateau. If so, the Bükk Mts. must have begun to be exhumed before the Dobi eruption, and existed as a lowland already emerging from the archipelago.

**Fig. 7:**
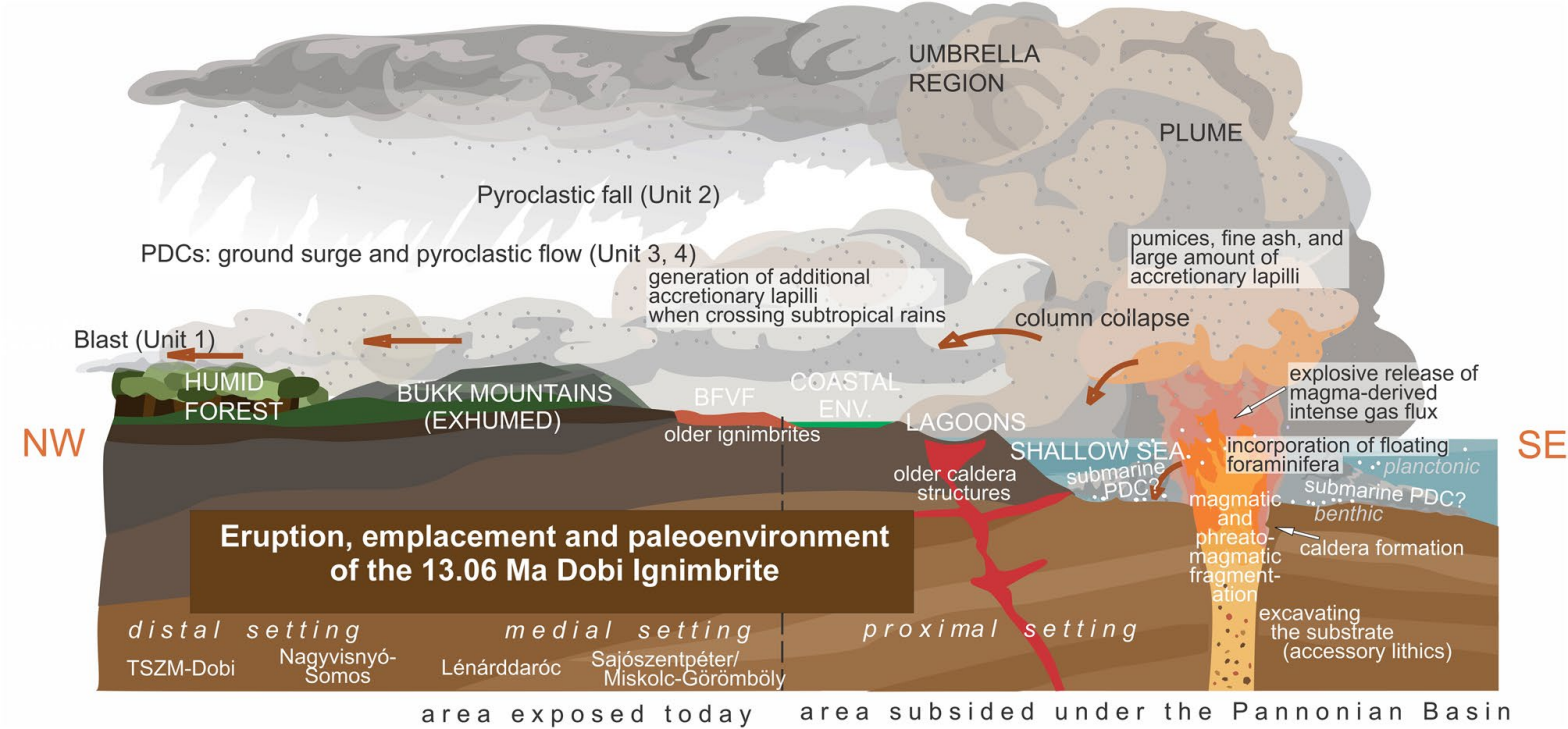
The eruption of the Dobi Ignimbrite as depicted on a virtual snapshot of the Latest Badenian paleogeography image; section oriented from NW to SE (for approximate position, see Fig. 2). BFVF = Bükk Foreland Volcanic Field. Image drawn in CorelDraw 2024 Graphic Suite.

In conclusion (Fig. 7), the Dobi eruption, sourced from a submarine vent, occurred in the coastal region of the Central Paratethys during the latest Badenian. Most of the pyroclastic succession was emplaced subaerially over and beyond the present-day BFVA to the NW and NE. Although only isolated occurrences of medial and distal facies have remained exposed due to basin subsidence or long-term erosion, the Dobi Ignimbrite conserved relics of the flourishing latest Badenian vegetation of the northern Pannonian Basin.

## Methods

### Granulometry of the ignimbrite

A standard, simple sieving procedure was applied to each ~ 0.5 kg of samples taken from Unit 4 ignimbrite of Sajószentpéter, Lénárddaróc and TSZM-Dobi site, respectively. After drying the samples at 80 °C for 24 h, they were carefully separated along grain boundaries by hand and brush, and then hand sieving was applied. Sieve diameters of 32,16, 8, 4, 2, 1, 0.5, 0.25, 0.125, 0.063 mm (corresponding to 4, 3, 2, 1, 0, -1, -2, -3, -4, -5 phi) were used. The classification was based on Inman (1952), where phi = log2(d) and d is the maximum clast diameter in mm. Granulometry statistics are presented in Supplement 2.

### Tree mold directional statistics

At the TSZM-Dobi, Sajószentpéter and Lénárddaróc sites, the plunges of the long axes of fossil tree trunk molds were measured with an iPad mini 4 using FieldMOVE. The results were spot-checked with a Breithaupt geological compass for consistency. The rose plots (Fig. 2, Supplement 3) were created with SG2PS^99^ with a 10° bin size, and were corrected for the bedding dip (112/23 for TSZM-Dobi, 59/11 for Sajószentpéter; no bedding dip available for Lénárddaróc) to account for post-depositional tectonic tilting, and to display the data in their original, 13.1 Ma, position. Supplement 3 shows all obtained data in a numerical format, and both the present-day and the tilt-corrected plunge directions of tree trunk molds.

### Geochemical analysis

Sampling of ignimbrites was carried out at four representative sites. Fresh glass fragments from the juvenile material mounted in epoxy were analyzed by electron microprobe at GEOMAR, Kiel (Germany) and by laser ablation inductively-coupled mass-spectrometry (LA-ICP-MS) at the Institute of Geosciences, Christian-Albrecht-University of Kiel (Germany). The samples were analyzed in the same series with those reported by Hencz et al.^13^ using reference materials and analytical conditions^13,100^. Data on reference glasses analyzed along with the complete analytical data of samples of this study are provided in Supplement 5A.

### Zircon U–Pb dating

^238^U/^206^Pb dating was performed at Université Clermont-Auvergne, Laboratoire Magmas et Volcans, Clermont-Ferrand (France). Zircons were separated from an ignimbrite sample of TSZM-Dobi site using standard techniques of crushing and sieving, followed by Wilfley table, magnetic separation, and heavy liquids before handpicking under a binocular microscope. Zircons were then mounted in epoxy disks, ground, and polished with 0.25 µm diamond grit to expose crystal interiors. U-Th-Pb isotopic data on zircons were obtained by laser ablation inductively coupled plasma mass spectrometry (LA-ICP-MS). Further analytical details as well as data and concordia diagram of the ^238^U/^206^Pb age of Dobi Ignimbrite can be found in Supplement 7.

### Sanidine and plagioclase Ar–Ar dating

^40^Ar/^39^Ar dating was performed at the LSCE ^40^Ar/^39^Ar facility CEA, Université Versailles St-Quentin and GEOPS, Université Paris-Saclay, Orsay (France), on ignimbrite samples taken from the TSZM-Dobi cellar and Lénárddaróc upper quarry. The ^40^Ar/^39^Ar ages were obtained on 21 crystals. The analytical procedure of ^40^Ar/^39^Ar dating for each sample, along with respective plots and evaluation, can be found in Supplement 8A, 8B. Weighted mean age uncertainties including J uncertainty were calculated using Isoplot 4.0. Measurements spreading of the Dobi and Lénárddaróc inverse isochron are around 40% and 60%, and the resulting ^40^Ar/^36^Ar initial intercepts are within the uncertainty of that of the atmosphere (296.9 ± 4.7 and 300.1 ± 2.9, respectively), suggesting that the dated crystals do not contain trapped excess argon.

### Paleomagnetic investigations

Forty drill cores were taken from five sites (TSZM-Dobi, Egercsehi, Lénárddaróc, Edelény, Sajószentpéter) (Supplement 9). Field inspection and/or the magnetic anisotropy measurements implied that the sampled ignimbrites were emplaced in horizontal position, while at TSZM-Dobi site tilt correction (112/23°) has been applied to the paleomagnetic vector. The tectonic tilting at Sajószentpéter was not evident in the paleomagnetic data. The samples contain a porous matrix of ignimbrite with pumice and lithic clasts (e.g., claystone). Sampling was carried out with a low voltage, electric portable drill. Each sample was oriented in situ, marked and numbered to aid subsequent sample preparation in the laboratory.

The samples were processed at the Supervisory Authority of Regulatory Affairs, Paleomagnetic Laboratory of the Geological Survey, Budapest (Hungary), using JR-4 and JR-5a spinner magnetometers and KLY-2 Kappabridge. The former allows the determination of the direction of the natural remanent magnetization (NRM), and the latter the value of the bulk magnetic susceptibility and its anisotropy (AMS). First, the NRM was measured in the initial state. Then, the samples were stepwise demagnetized thermally (and the magnetization left re-measured after each step), thus the full spectrum of possible magnetic components of the samples was obtained. The demagnetization was made in numerous steps on “pilot” samples from each site from 150 up to 575 °C (in 50 °C steps up to 500 °C, then in 25 °C steps) to eliminate secondary magnetization acquired after the ignimbrite cooled. Based on the demagnetization behaviour of the pilot samples, the remaining ones were demagnetized in fewer steps. Following the first NRM measurement and each subsequent demagnetization step, the magnetic susceptibility was also measured to detect the possible changes in the magnetic minerals. The paleomagnetic direction of individual samples were calculated using principal component analysis^56^, while the paleomagnetic mean direction and statistical parameters of sites were calculated using the Fisher method^58^. The mean directions were plotted on stereographic projections using the PMGSC software. When the sampled ignimbrite was tilted (Dobi site), the paleomagnetic direction was corrected: the direction-correction tilt test^39^ is positive.

### Constructing the gravity Bouguer anomaly and dT magnetic anomaly map

Hungary’s geophysical database includes over 380,000 gravity measurements averaging about 4 points per km^2^. From these measurements, using various corrections, we determined the gravity influence of the rocks that make up the crust, i.e., the Bouguer anomaly map. The map shown in Supplement 10 is determined by the gravity effect of causative bodies (geological formations) at different depths and densities. That is, sudden density jumps or sudden depth changes appear on the map as Bouguer anomalies.

The database also contains over 112,000 magnetic survey points, covering roughly 0.8 points/km^2^, with full coverage provided by 76,000 ΔZ measurements from vertical magnetic surveys. After standardization (base level and magnetic normal field correction) of the measured relative values, the magnetic map shows the magnetic influence of the rocks in the crust. The shape, magnetization, geometry and depth position of the magnetic bodies determine the shape (magnitude, amplitude, wavelength) of the anomaly that is generated through a complex relationship. The overall picture can be significantly simplified by representing the variability, i.e., the variance of ΔZ values per unit area. This value will be the largest where the magnetic field is located, or at its edges in the case of large bodies.

## Supplementary Information

Supplementary Information 1.

Supplementary Information 2.

Supplementary Information 3.

Supplementary Information 4.

Supplementary Information 5A.

Supplementary Information 5B.

Supplementary Information 6.

Supplementary Information 7.

Supplementary Information 8A.

Supplementary Information 8B.

Supplementary Information 9.

Supplementary Information 10.

## Data Availability

All data generated or analysed during this study are included in this published article and its Supplementary Information Files 1–10.
