# Supplementary material for: A 13.06 Ma widespread ignimbrite in the Pannonian Basin captured a snapshot of shallow marine to coastal environment in Central Paratethys
Source: Sci Rep. 2025 Jul 2;15:23528. doi: 10.1038/s41598-025-07002-9 (PMC12223212; doi:10.1038/s41598-025-07002-9)
Supplement: Supplementary file 1 — Supplementary Information 1. [file 41598_2025_7002_MOESM1_ESM.pdf]

Site and sample code (arranged from W to E)	*Coordinates*	*Observed/ inferred thickness (m)*	*5 max. pumice size(cm)*	*Thin sec-tion*	*Geo-chem-istry*	*Paleo-mag-netism*	*Dating*	*Geological and volcanological description*
1. Tarnaszentmária-Dobi cellar TSZM-DOBI	47°52'52.4"N 20°12'38.8"E	5	<1	+	+	+	Ar-Ar U-Pb	Contact of marine (claystone-siltstone) basement with paleosol on top, and the pyroclastic succession. Lithified soil overlain by 1-2 cm blast / ground surge (preserving leaf and stalk remains); then 15-20 cm fine-grained pumice fall (with mm-sized pumices); then again 6-8 cm surge; followed by 5 m fine-to coarse grained, unwelded, massive pumice-flow deposit (ignimbrite). Fine tuff matrix contains moderate amount of accr. lapilli, evenly distibuted and also in gas escape pipes. Lower part of ignimbrite contains charred tree trunks and tree trunk molds
2. Irma-major quarry	47°53'16.3"N 20°13'58.0" E	15	<3					15 m fine-grained, massive pumice-flow deposit (ignimbrite); moderate amount of accr. lapilli, evenly distributed, and also in gas escape pipes
3. Egercsehi quarry EGERCS	48°2'32.0" N 20°16'05.1"E	10	<1	+		+		10 m fine-grained, massive pumice-flow deposit (ignimbrite) exposed in a quarry; small amount of accr. lapilli, and also in gas escape pipes
4. Lénárddaróc quarry LENARD_ PHREAT_1	48°8'49.6" N 20°21'38.1"E	50	9, 7, 5, 4, 3	+	+	+	Ar-Ar	A 50 m-thick hillslide exposing massive ignimbrite on bottom (with accr. lapilli) and uphill in a double quarry. In the lower of these, an intercalated reworked epiclastic unit (<1 m) with cross-bedding indicates a time gap between two eruption phases. Accr. lapilli occur only in the upper quarry. Tree trunk molds are frequent, mostly in the lower quarry
5. Nagyvisnyó Nvk-3 borehole NVK_3	48°04'31.4" N 20°29'44.2" E	<<1		+				Reworked fine tuffaceous material in upper part of borehole with identical mineral assemblage to Dobi ignimbrite
6. Nagyvisnyó Somos valley* SOM_532	48°09'30" ? N 20°27'15" ? E	?	?	+	+			A single sample of leaf-bearing fine-grained primary fine tuff with identical mineral assemblage and chemistry to Dobi ignimbrite.
7. Sajószentpéter cellars: SAJSZP_PIN_F upper SAJSZP_PIN_A lower	48°12'34.6" N 20°42'46.0" E	80	19, 17, 16.5, 15, 14	+	+	+		A hillside with cellars and paths in between, <80 m thickness in total, the whole of which is made up of massive ignimbrite. Accr. lapilli often in gas escape pipes are frequent. Two types of pumices: white clast with large quartz and biotite phenocrysts, and dark grey clast with vesicular, fibrous texture and no or minor observable crystals. Trere trunk molds frequently occur
8. Edelény cellars	48°19'29.7" N 20°42'14.3" E	30	10, 6, 5, 5, 4			+		Two cellars reveal massive ignimbrite, with accr. lapilli (often in gas escape pipes) and two types of pumices as described above
9. Miskolc-Görömböly cellars MIS_GOROMB	48°3'3.4" N 20°46'23.5" E	?	20, 18, 17, 14, 13	+				Poorly exposed hillside with cellars. Accr. lapilli often in gas escape pipes are frequent. Total deposit thickness cannot be assessed, but it is probably partly covered due to the closeness of the Nyékládháza-1 borehole
10. Nyékládháza-1 borehole** NYA_1	48°01'14.9" N 20°46'48.2" E	44+22=66	20–30**	+			K-Ar[4]	Both unit C1 and C1 start with 1-2 cm fine tuff interpreted as ground surge, followed by 25-30 cm pumice fall, then thick, massive ignimbrite
11. Sárospatak Megyer Hill quarry*** (SP-MH-1)	48°21'26.3" N 21°34'22.3" E	30	<6-8[1]				U-Pb[5]	Monotonous, massive ignimbrite, heavily altered (silificied, zeolitized); pumice fragments are removed by weathering

12. Somlyód quarry	48°21'20.1" N 21°35'32.8" E	5-10	<1-2				K-Ar[6]	Fine-grained ignimbrite – silicified, zeolitized
13. Mikóháza MIK-1***	48°27'20.5" N 21°34'45.5" E	5-8	<1-2[2]	+			U-Pb[5]	Slightly reworked pumiceous volcaniclastic succession in river bed – silicified, zeolitized
14. Mikóháza road cut (MIK_2)	48°27'27" N 21°34'59" E	?	<1-2	+				Fine-grained ignimbrite with some reworking (pumice lenses) – silicified, zeolitized
15. Vilyvitány (hillslope) VILY-1***	48°30'2.3" N 21°33'42.1"E	10-15	<1-2[3]	+			U-Pb[5]	Fine-grained ignimbrite – silicified, zeolitized

*leaf-bearing tuff samples, stored in the Hungarian Natural History Museum, were collected by István Gaál (1949), unpublished
**borehole succession is described first in Lukács et al. (2010); thickness data are taken from their unit C1 and C2; up to 30 cm pumice size is reported
***sites are identical to those in Lukács et al. (2024) with abbreviated sample names; thickness data taken from their *ESM_All_data table of Supplement*

[1]pumice size taken from Lukács et al. (2024)[30], although it is difficult to assess due to the alteration and removal of pumice clasts
[2]>15 cm size in Lukács et al. (2024)[30], but we find this value unsupported based on field survey and thin section inspection
[3]>25 cm size in Lukács et al. (2024)[30], but we find this value unsupported based on field survey and thin section inspection
[4]Lukács et al. (2010)[65]
[5]Lukács et al. (2024)[30]
[6]Pécskay et al. (1986)[35]
