# Supplementary material for: A 13.06 Ma widespread ignimbrite in the Pannonian Basin captured a snapshot of shallow marine to coastal environment in Central Paratethys
Source: Sci Rep. 2025 Jul 2;15:23528. doi: 10.1038/s41598-025-07002-9 (PMC12223212; doi:10.1038/s41598-025-07002-9)

Phi		SUM	%	%_SUM
	Sajószentpéter	473.7		
-5	>32 mm	8.7	1.84%	1.84%
-4	16 mm	7.8	1.65%	3.48%
-3	8 mm	9.6	2.03%	5.51%
-2	4 mm	26.4	5.57%	11.08%
-1	2 mm	67.2	14.19%	25.27%
0	1 mm	82.8	17.48%	42.75%
1	500 µm	72	15.20%	57.95%
2	250 µm	64.8	13.68%	71.63%
3	125 µm	57.6	12.16%	83.79%
4	63 µm	44.4	9.37%	93.16%
>4	< 63µm	32.4	6.84%	100.00%

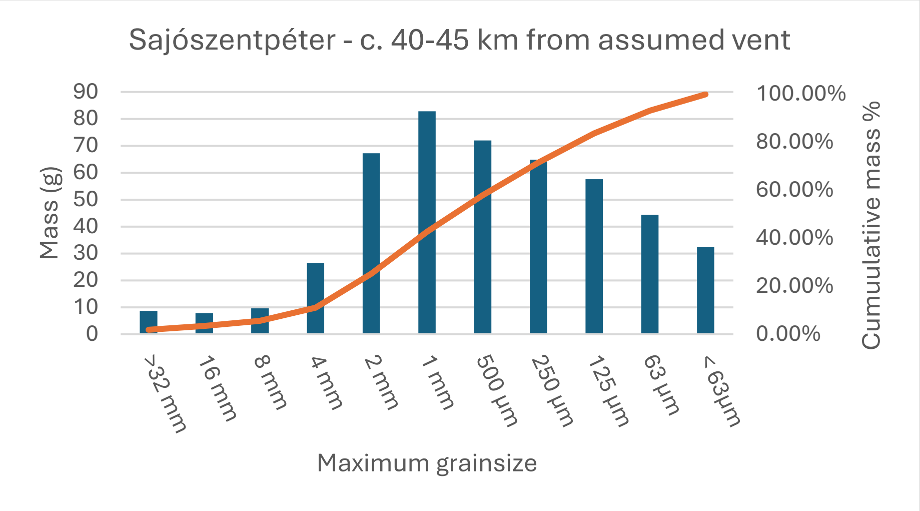

	Lénárddaróc	179.7		
-5	>32 mm	3.2	1.78%	1.78%
-4	16 mm	4.3	2.39%	4.17%
-3	8 mm	2.7	1.50%	5.68%
-2	4 mm	6.3	3.51%	9.18%
-1	2 mm	8.4	4.67%	13.86%
0	1 mm	19.5	10.85%	24.71%
1	500 µm	24.9	13.86%	38.56%
2	250 µm	27.6	15.36%	53.92%
3	125 µm	30	16.69%	70.62%
4	63 µm	26.1	14.52%	85.14%
>4	< 63µm	26.7	14.86%	100.00%

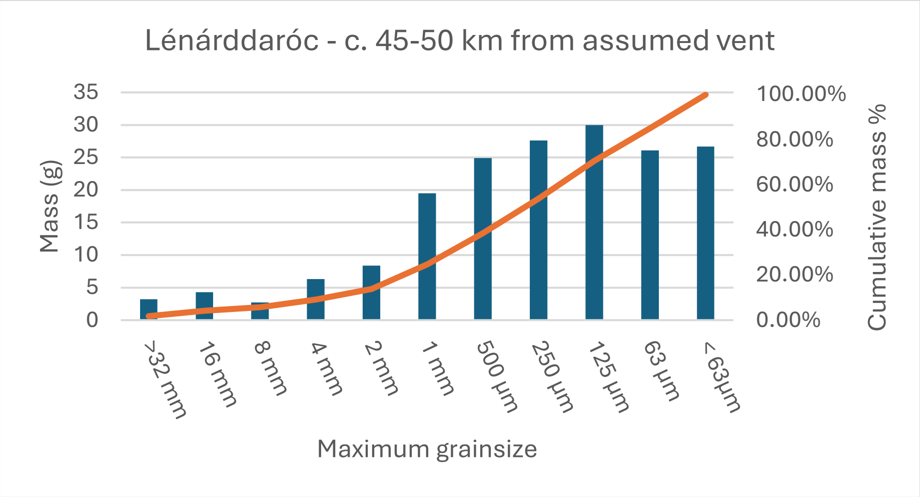

	TSZM-Dobi	354.9		
-5	>36 mm		0.00%	0.00%
-4	16 mm		0.00%	0.00%
-3	8 mm	1.4	0.39%	0.39%
-2	4 mm	4.9	1.38%	1.78%
-1	2 mm	13.3	3.75%	5.52%
0	1 mm	24.5	6.90%	12.43%
1	500 µm	42	11.83%	24.26%
2	250 µm	60.9	17.16%	41.42%
3	125 µm	73.5	20.71%	62.13%
4	63 µm	72.8	20.51%	82.64%
5	35 µm	21	5.92%	88.56%
>5	< 35µm	40.6	11.44%	100.00%

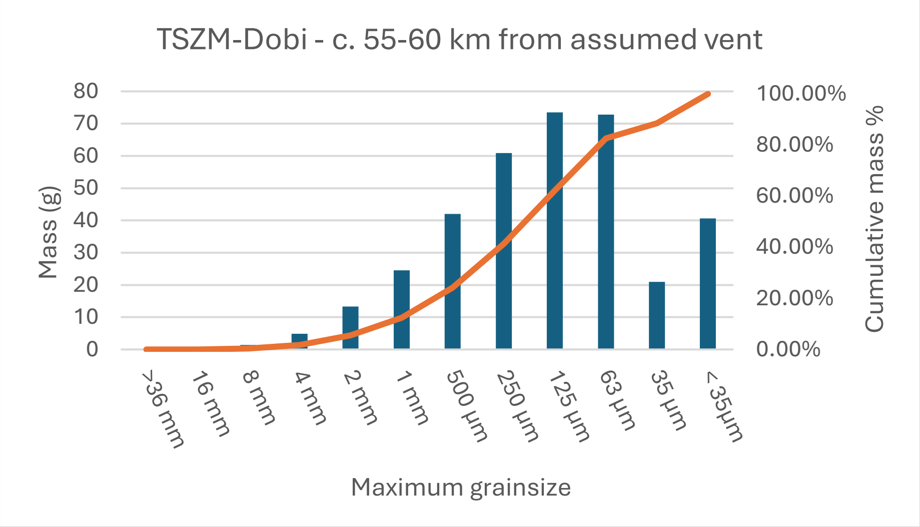

Site	Phi_16	Phi_84	Md_Phi	Rho_Phi
Sajószentpéter	-1.65	3.02	0.48	2.34
Lénárddaróc	-0.80	3.92	1.74	2.36
TSZM-Dobi	0.30	4.23	2.41	1.96

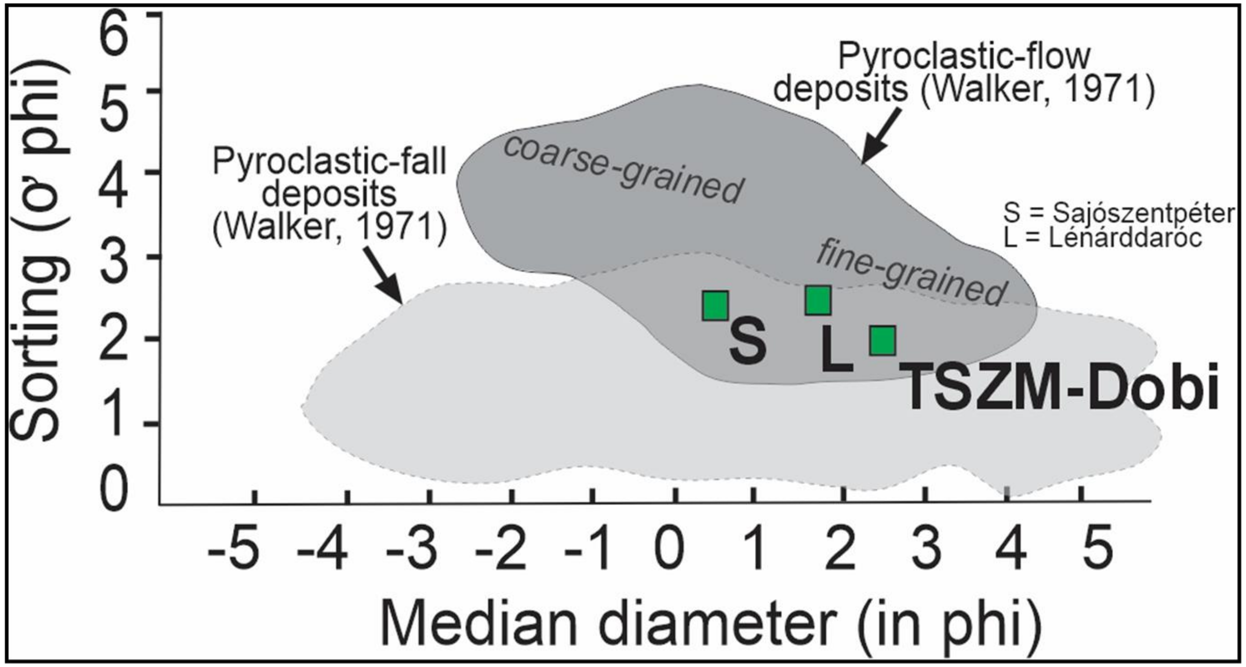

Supplement: Supplementary file 2 — Supplementary Information 2. [file 41598_2025_7002_MOESM2_ESM.pdf]
