# Supplementary material for: A 13.06 Ma widespread ignimbrite in the Pannonian Basin captured a snapshot of shallow marine to coastal environment in Central Paratethys
Source: Sci Rep. 2025 Jul 2;15:23528. doi: 10.1038/s41598-025-07002-9 (PMC12223212; doi:10.1038/s41598-025-07002-9)
Supplement: Supplementary file 3 — Supplementary Information 3. [file 41598_2025_7002_MOESM3_ESM.pdf]

Supplement 3 - Tree trunk mould orientation data

DATA_ID	GROUP_CODE	COLOR_CODE	LOCATION	LOC_X	LOC_Y	FORMATION	DATATYPE	DIP_DIR	DIP	L_DIP_DIR	L_DIP	SENSE	PALEONORTH	COMMENT	diameter (cm)	minimum length (cm)
b7d6a41507c7e740d2c5574b87ac567d			Dobi-oldal			Dobi_ignimbrite	bedding	112	23							
79ca6b8104d0106f74ac72ef36f36454			Dobi-oldal			Dobi_ignimbrite	bedding	113	23							
517390b42d9944b40605d5952ab2cf62			Dobi-oldal			Dobi_ignimbrite	userlineation1	30	14							
5ea1d8da2857088e124de932a5184052			Dobi-oldal			Dobi_ignimbrite	userlineation1	49	10							
c388f45d885fbfaebebc06ed681fd5c5			Dobi-oldal			Dobi_ignimbrite	userlineation1	70	6							
287cd3b43955e367f9285b34af1c967b			Dobi-oldal			Dobi_ignimbrite	userlineation1	79	26							
1ac7845ebe9729e27eab86137f0774a2			Dobi-oldal			Dobi_ignimbrite	userlineation1	85	19							
58c9a05a981c733e378444d8b5d863c0			Dobi-oldal			Dobi_ignimbrite	userlineation1	137	13							
a0113b4fc8c5547e83fc44b9b514e2fd			Dobi-oldal			Dobi_ignimbrite	userlineation1	161	6							
e2ee11b8a0cd022425f69d044988ad63			Dobi-oldal			Dobi_ignimbrite	userlineation1	169	13							
85c4469a8bd825731c498ebcf1ee7761			Dobi-oldal			Dobi_ignimbrite	userlineation1	243	6							
053fbd5abbc70734e91b377b689ef069			Dobi-oldal			Dobi_ignimbrite	userlineation1	283	20							
158fc3e4e9f67757aa048938f5c07c9a			Sajoszentpeter			Dobi_ignimbrite	bedding	59	11							
a02959f18235698457e717a124473096			Sajoszentpeter			Dobi_ignimbrite	userlineation1	2	6							
fcfe61e23cad68792d02763f52078f0b			Sajoszentpeter			Dobi_ignimbrite	userlineation1	10	12							
bda1391dfe6f3a6d3bff5d3931b2cc90			Sajoszentpeter			Dobi_ignimbrite	userlineation1	31	17							
35279e1de682ca72b728f528c1d100b4			Sajoszentpeter			Dobi_ignimbrite	userlineation1	136	9							
69b9ef2200325b06f921dbd4fbba5986			Sajoszentpeter			Dobi_ignimbrite	userlineation1	159	2							
575c090d73d3c40c89737dad46d2d517			Sajoszentpeter			Dobi_ignimbrite	userlineation1	346	7							
b8c009c72370de746cac770ca805c814	A		Lenarddaroc			Dobi_ignimbrite	userlineation1	298	14					bark imprint	5	40
c96e4f88631c605df907cd34b67b99c3	A		Lenarddaroc			Dobi_ignimbrite	userlineation1	300	11					bark imprint	5	25
9bb2f0f07022bad760611f755e5eae45	A		Lenarddaroc			Dobi_ignimbrite	userlineation1	301	8					bark imprint	7	10
0760a78daef47e7eaec6854a688c439d			Lenarddaroc			Dobi_ignimbrite	userlineation1	43	24						30	150
9f92802ffd06280afabf111e6d89b7d7			Lenarddaroc			Dobi_ignimbrite	userlineation1	196	22						16	100
3e19527ee050d55a9826559946af43c9			Lenarddaroc			Dobi_ignimbrite	userlineation1	152	5						7	50
b2dc4363420073b907e719a9279754a2			Lenarddaroc			Dobi_ignimbrite	userlineation1	352	2						10	50
56837c09e4f617cc8f946d75bc887825			Lenarddaroc			Dobi_ignimbrite	userlineation1	238	7						7	40
33429fd6971354ff3fd758be3725119a			Lenarddaroc			Dobi_ignimbrite	userlineation1	241	21						10	40
03bfb8a3bd17f5e235964ae7a200693b			Lenarddaroc			Dobi_ignimbrite	userlineation1	184	13						5	35
a9bcb39a3e63d2373c8032d7e2b3d74e			Lenarddaroc			Dobi_ignimbrite	userlineation1	149	2						4	25
c976dbb0b2237461b6b46506cb9c98d4			Lenarddaroc			Dobi_ignimbrite	userlineation1	168	37						5	25
2be6033d02cc7d43c207133a6bc9f5ba			Lenarddaroc			Dobi_ignimbrite	userlineation1	208	8						2	20
b8ec14694d5ba7ef4fc7fb173637bf0f			Lenarddaroc			Dobi_ignimbrite	userlineation1	189	14						3	20
50b175287291dd10792b80cef5cf5609			Lenarddaroc			Dobi_ignimbrite	userlineation1	163	26						6	20
9b58412a0dc9e4c954c3c0d378d5e25e			Lenarddaroc			Dobi_ignimbrite	userlineation1	166	14						4	15
86f85d34dc969d0a99197d4a70acf788			Lenarddaroc			Dobi_ignimbrite	userlineation1	168	26						3	10

Methodology:
sp2ps.eu

Sasvári, Á. and Baharev, A. 2014: SG2PS (Structural Geology to Post Script Converter) – A graphical solution for brittle structural data evaluation and paleostress calculation. Computers and Geosciences 66, 81-93. https://www.sciencedirect.com/science/article/pii/S0098300413003166?via%3Dihub

Userlineation1 corresponds to tree mould lineation data.

BEDDING FROM LOCATION DOBI-OLDAL

Data number: 2
Average bedding: 112/23

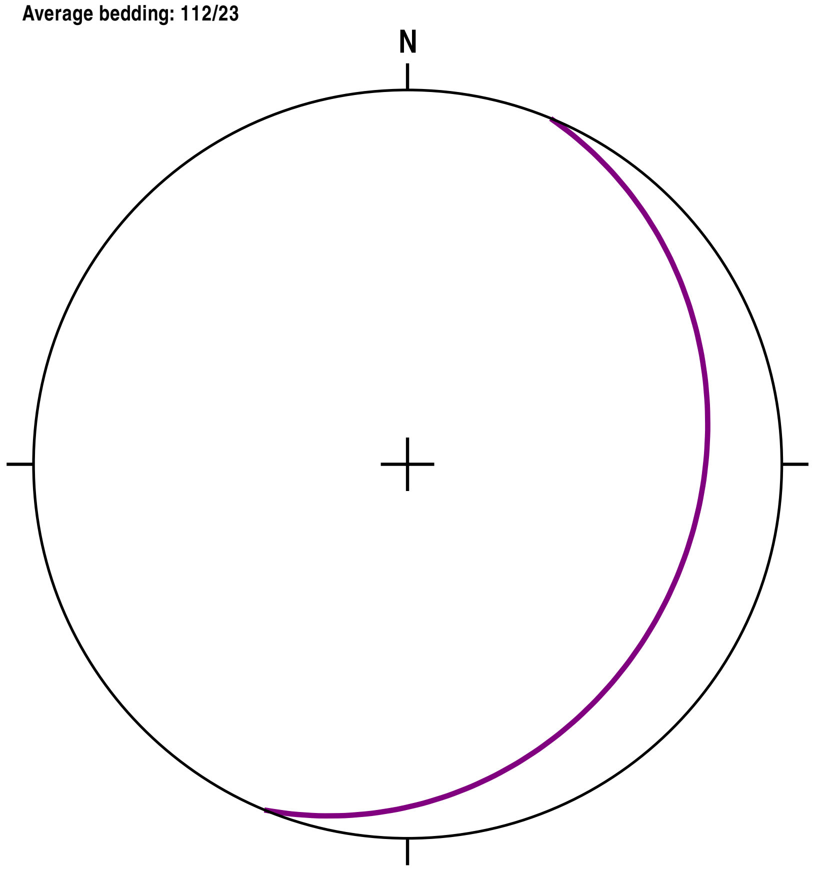

Angelier-plot, Schmidt-net, lower hemisphere

Rose plot for measured data
Average bedding: 112/23

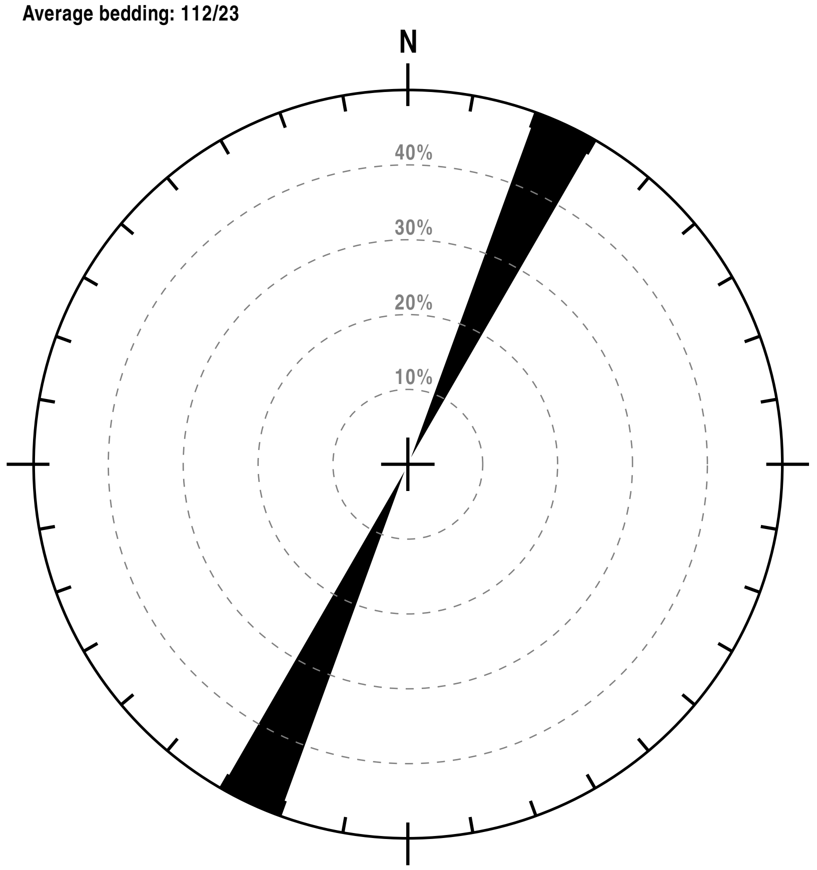

ORIGINAL

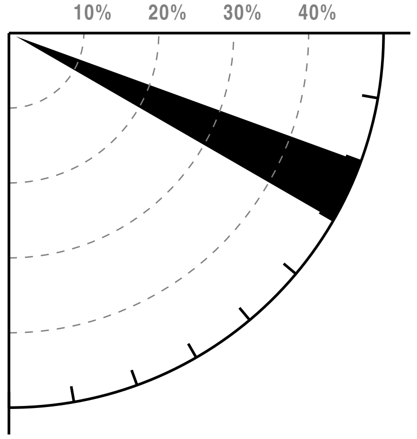

SYMBOLS

PLANES	BINGHAM STATISTICS	ROSE PLOT	GROUPS
Bedding		Plane strike direction	Default group
Bedding Overturned			
Average bedding			
Average bedding Overturned			

Data number: 2
Corrected by the average bedding: 112/23
Corrected by palaeo north directions

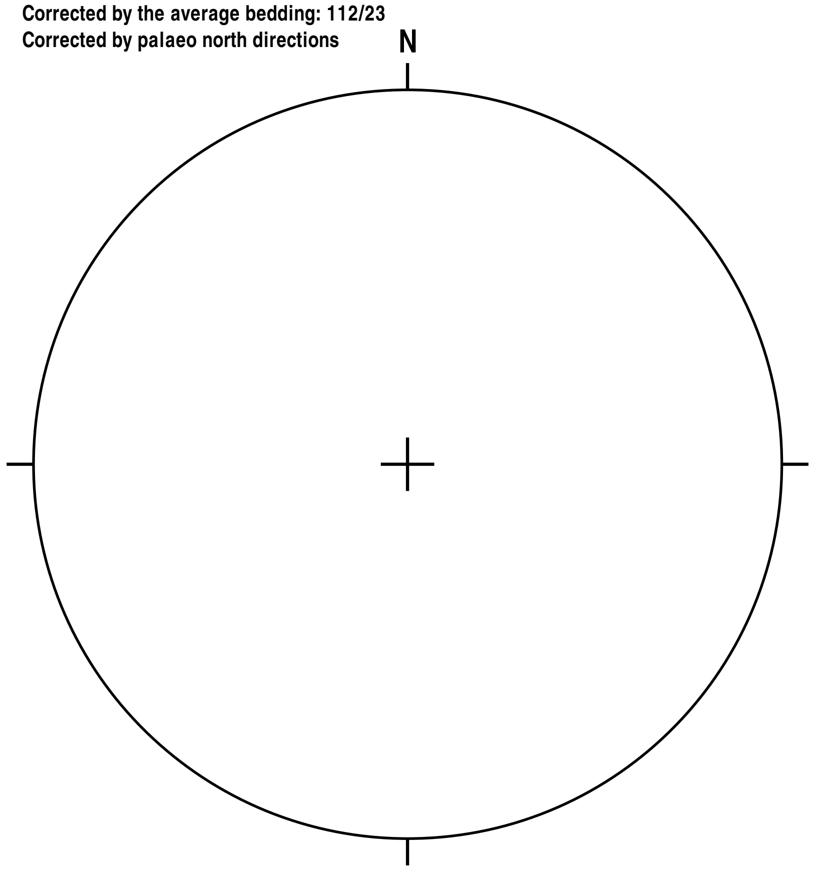

Angelier-plot, Schmidt-net, lower hemisphere

Rose plot for dip corrected data
Corrected by the average bedding: 112/23
Corrected by palaeo north directions

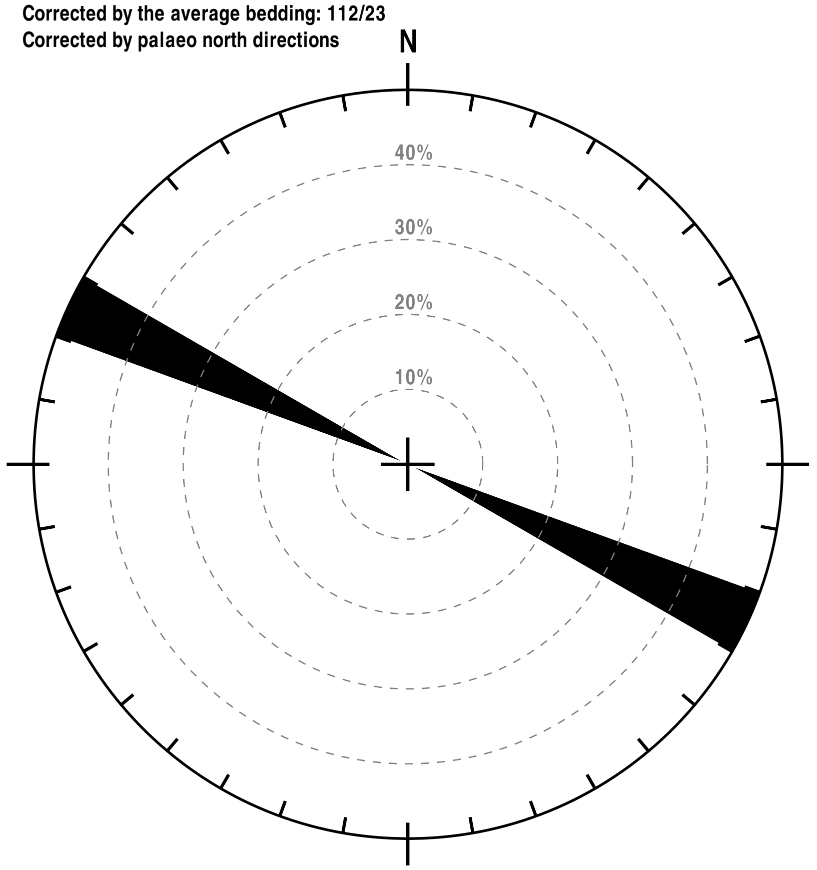

CORRECTED

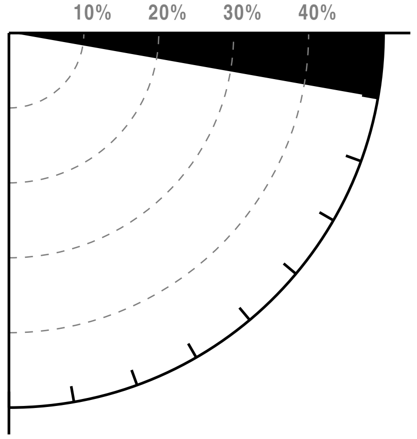

File location: 20240606-134140_DOBI / 5_PS_SEPARATED / BEDDING / DOBI-OLDAL_BEDDING_.EPS
Plotted by SG2PS (version: Jul 1 2017, 23:50:18) - for reference see www.sg2ps.eu webpage.

BEDDING FROM LOCATION SAJOSZENTPETER

ORIGINAL

Data number: 1
Average bedding: 059/11

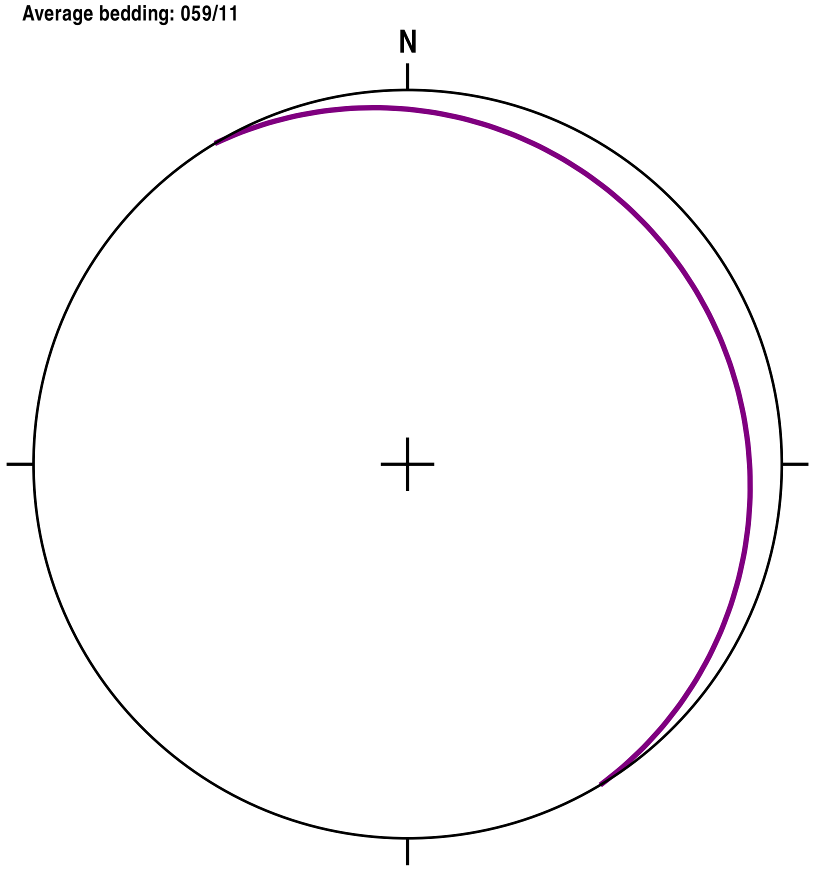

Angelier-plot, Schmidt-net, lower hemisphere

Rose plot for measured data
Average bedding: 059/11

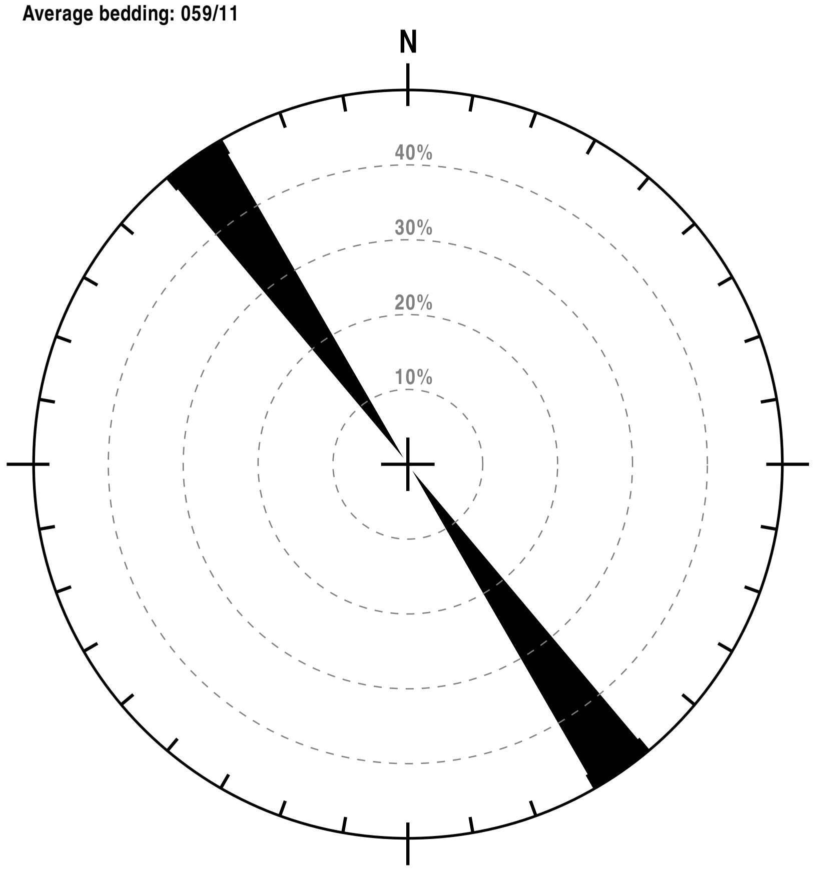

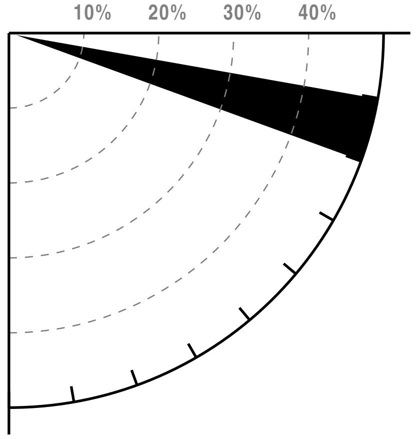

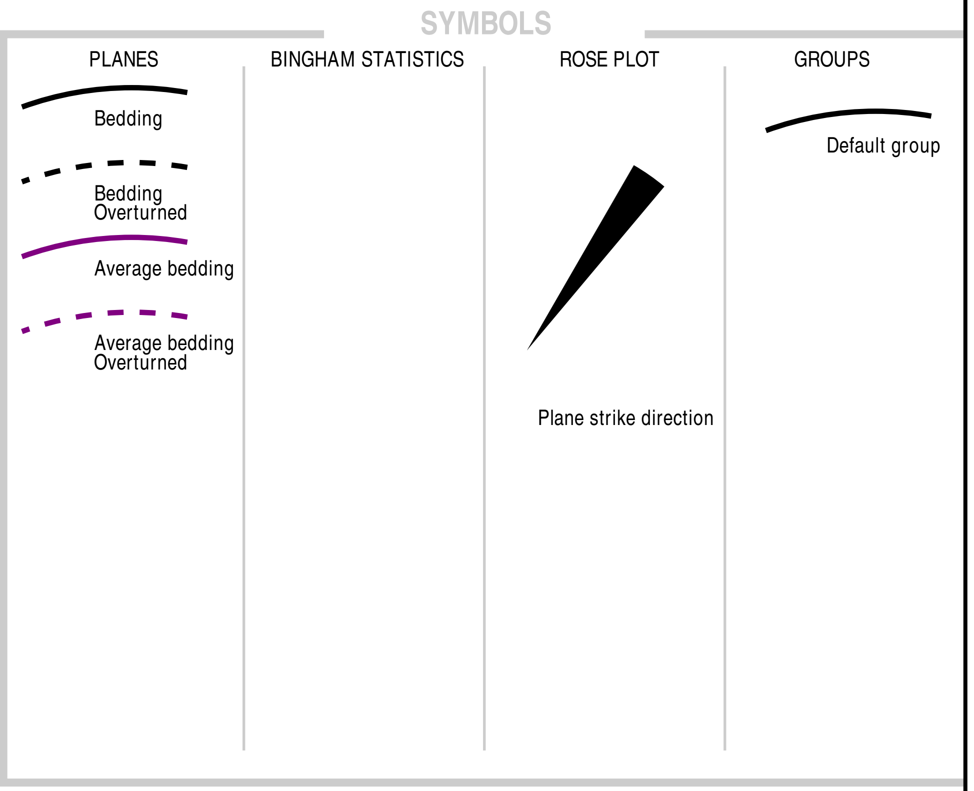

CORRECTED

Data number: 1
Corrected by the average bedding: 059/11
Corrected by palaeo north directions

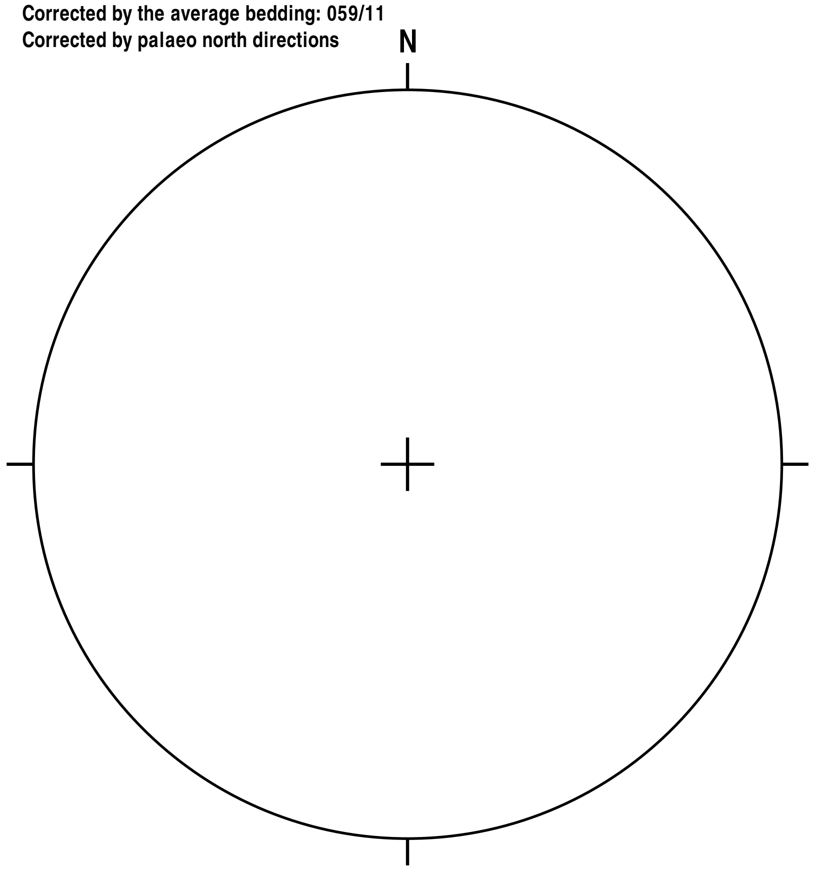

Angelier-plot, Schmidt-net, lower hemisphere

Rose plot for dip corrected data
Corrected by the average bedding: 059/11
Corrected by palaeo north directions

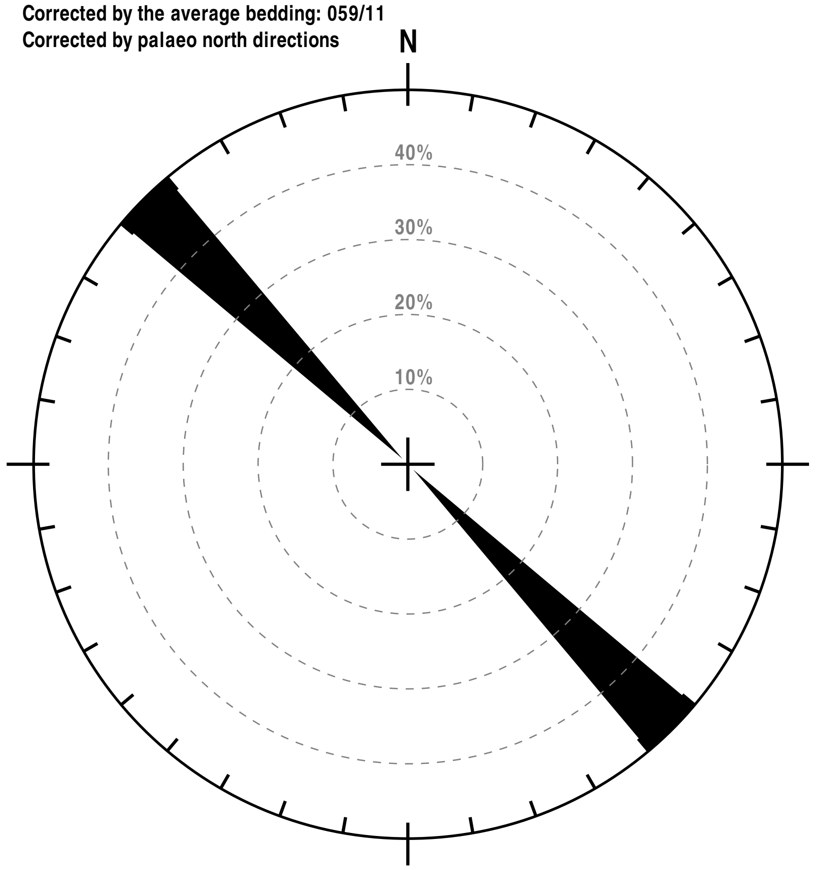

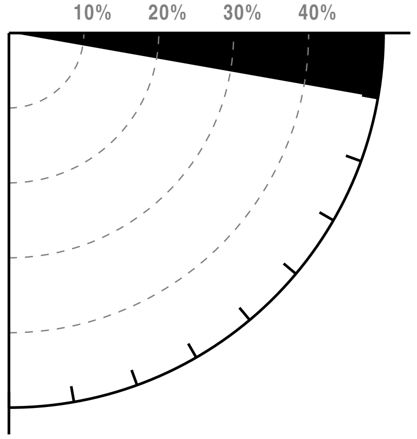

File location: 20240606-134140_DOBI / 5_PS_SEPARATED / BEDDING / SAJOSZENTPETER_BEDDING_.EPS
Plotted by SG2PS (version: Jul 1 2017, 23:50:18) - for reference see www.sg2ps.eu webpage.

BEDDING FROM LOCATION TARNASZENTMARIA-NY

Data number: 2
Average bedding: 119/22

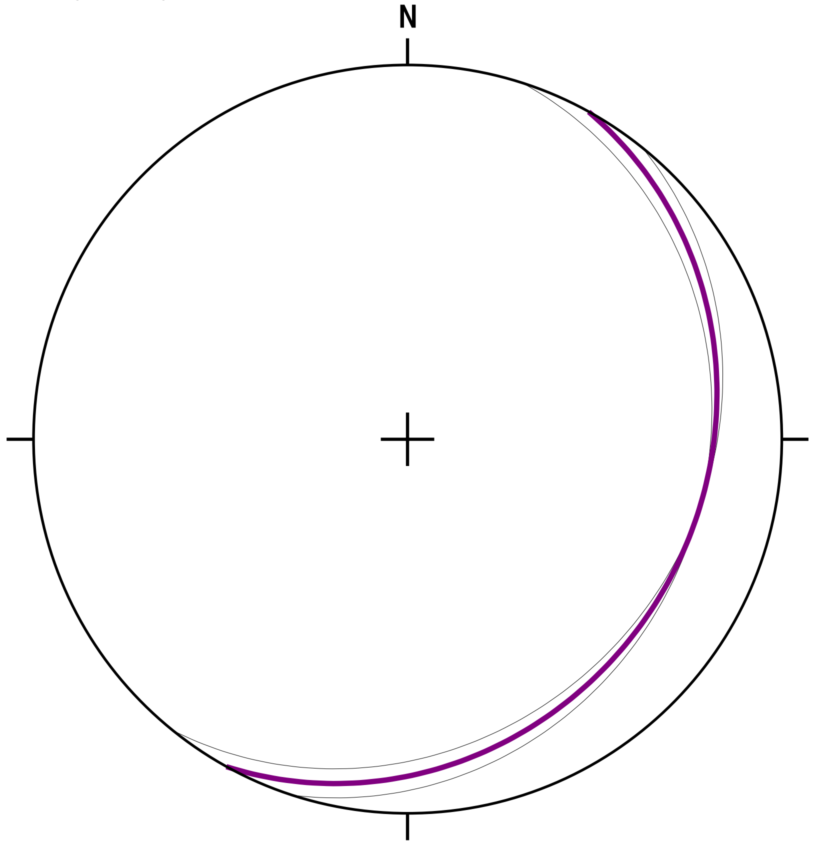

Angelier-plot, Schmidt-net, lower hemisphere

Rose plot for measured data
Average bedding: 119/22

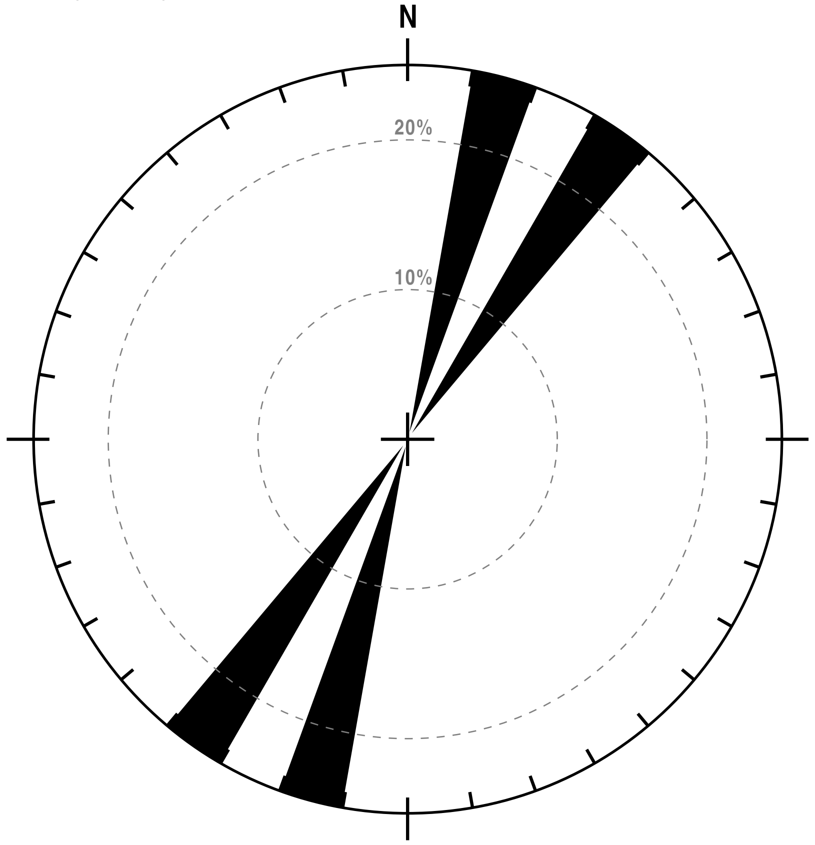

ORIGINAL

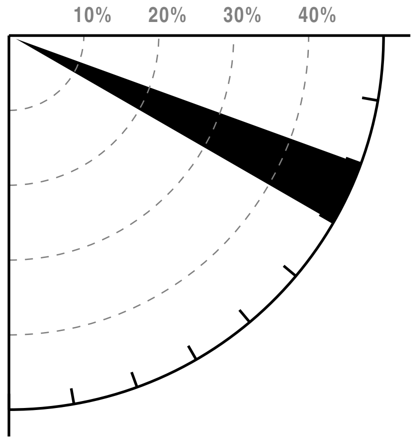

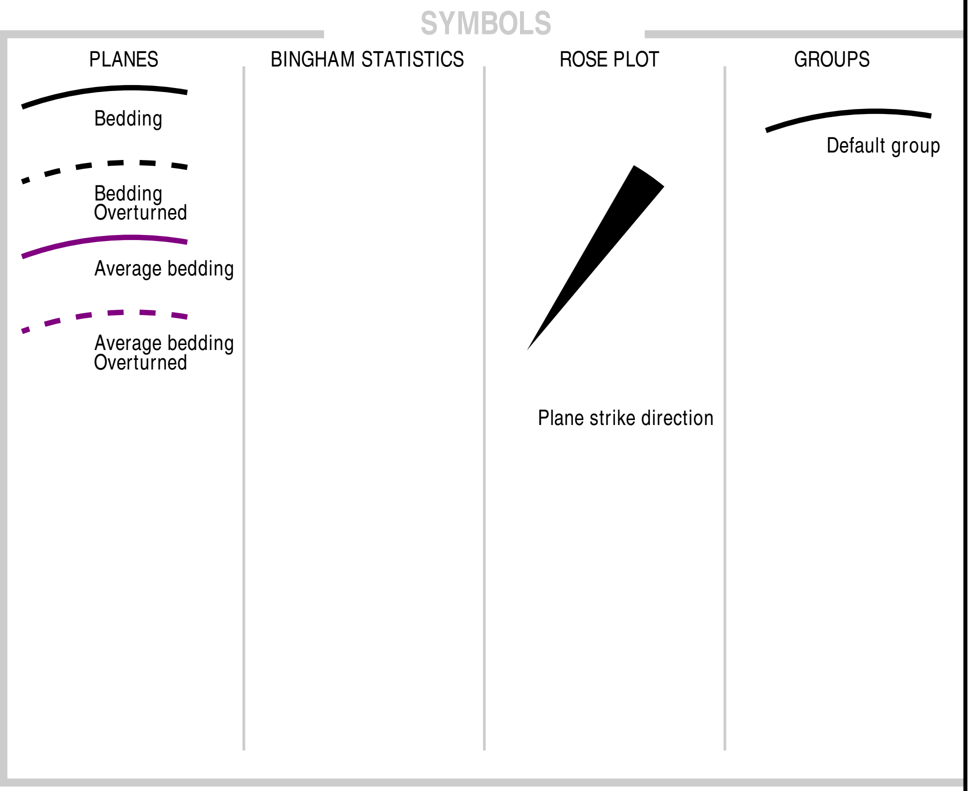

CORRECTED

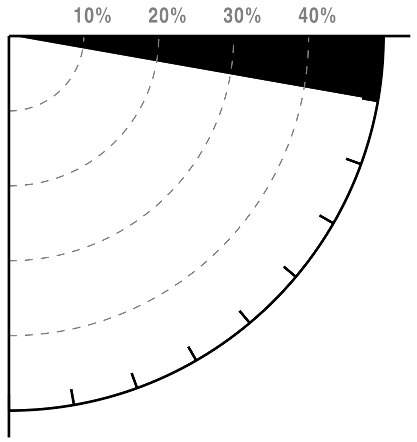

Data number: 2
Corrected by the average bedding: 119/22
Corrected by palaeo north directions

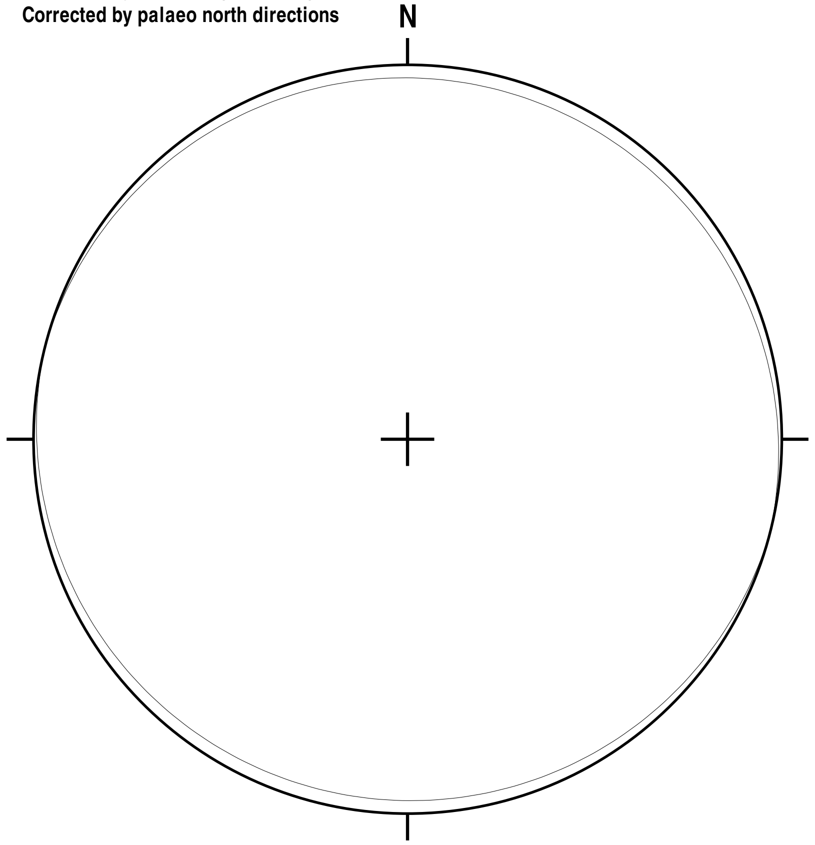

Angelier-plot, Schmidt-net, lower hemisphere

Rose plot for dip corrected data
Corrected by the average bedding: 119/22
Corrected by palaeo north directions

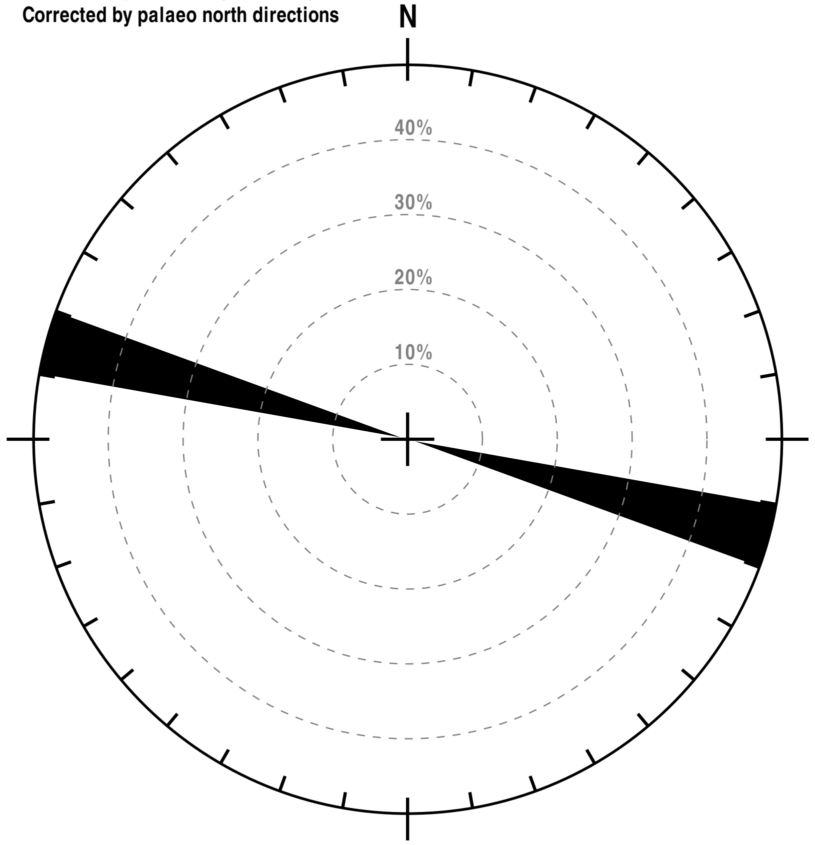

File location: 20240606-134140_DOBI / 5_PS_SEPARATED / BEDDING / TARNASZENTMARIA-NY_BEDDING_.EPS
Plotted by SG2PS (version: Jul 1 2017, 23:50:18) - for reference see www.sg2ps.eu webpage.

USERLINEATION1 FROM LOCATION DOBI-OLDAL

ORIGINAL

Data number: 10
Average bedding: 112/23

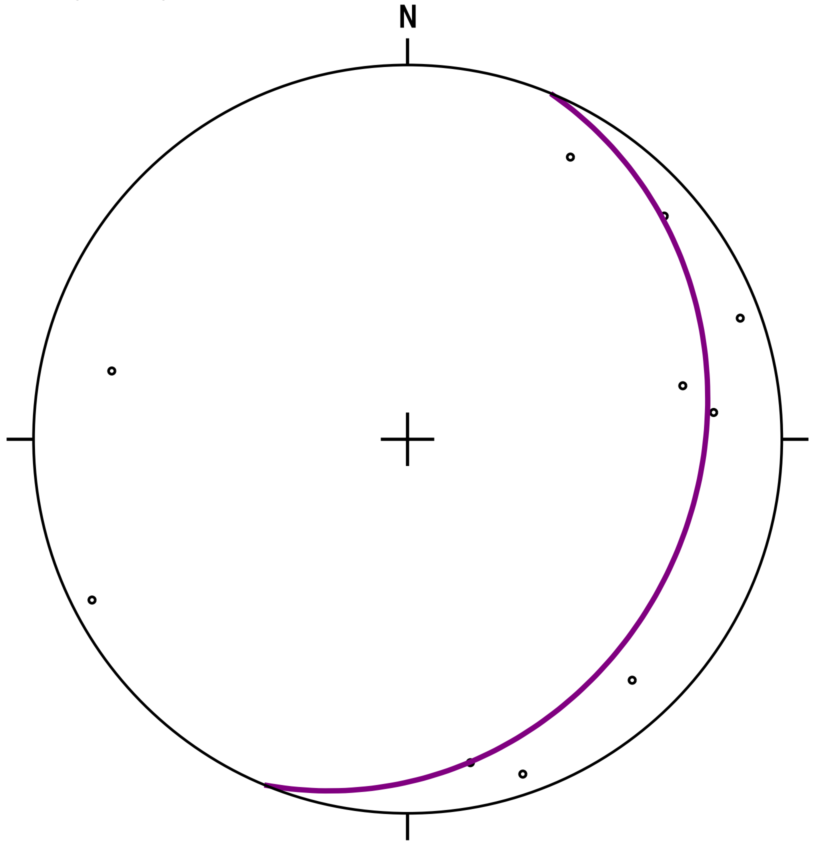

Angelier-plot, Schmidt-net, lower hemisphere

Rose plot for measured data
Average bedding: 112/23

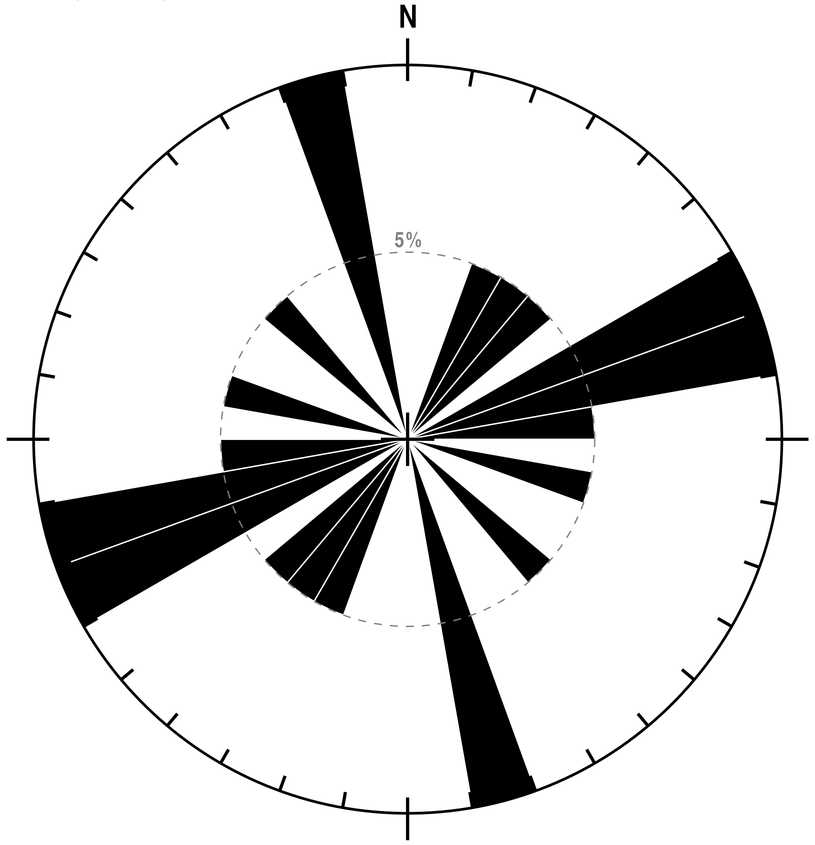

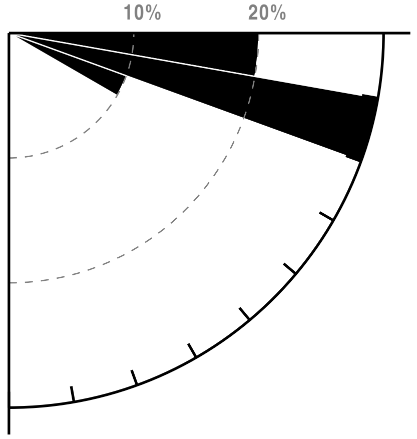

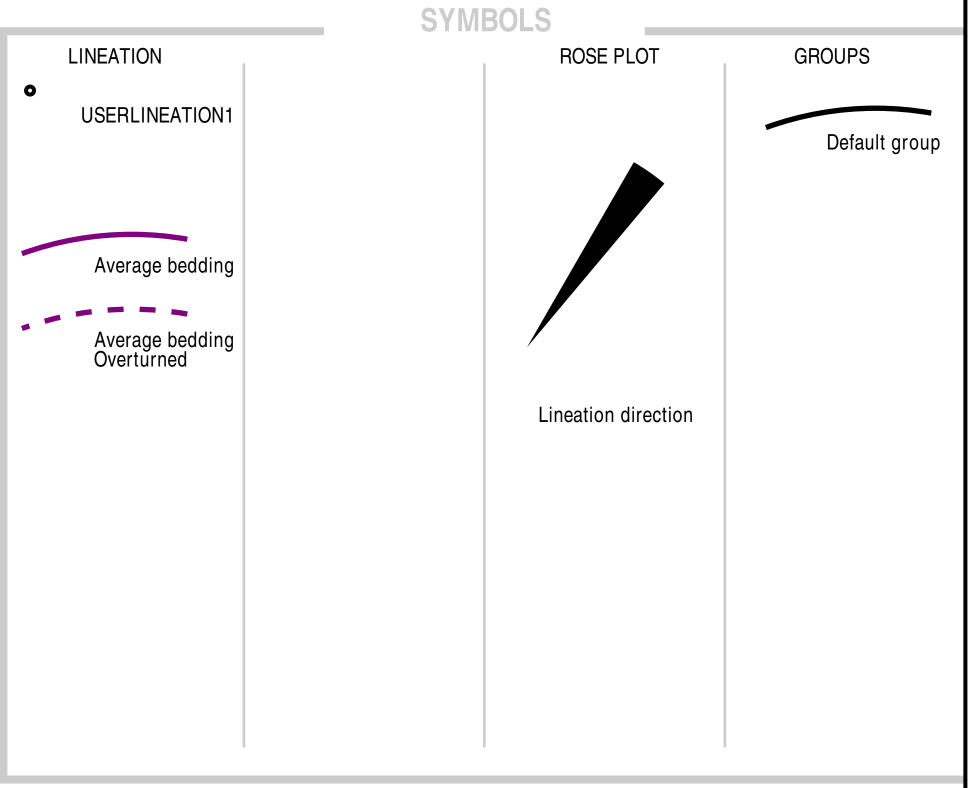

CORRECTED

Data number: 10
Corrected by the average bedding: 112/23
Corrected by palaeo north directions

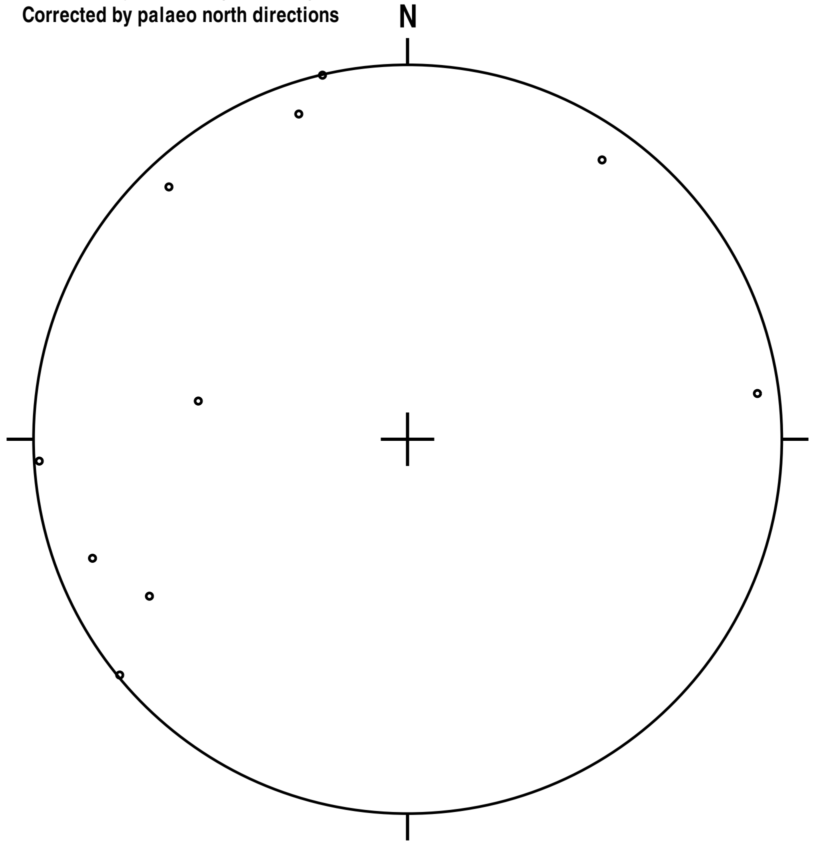

Angelier-plot, Schmidt-net, lower hemisphere

Rose plot for dip corrected data
Corrected by the average bedding: 112/23
Corrected by palaeo north directions

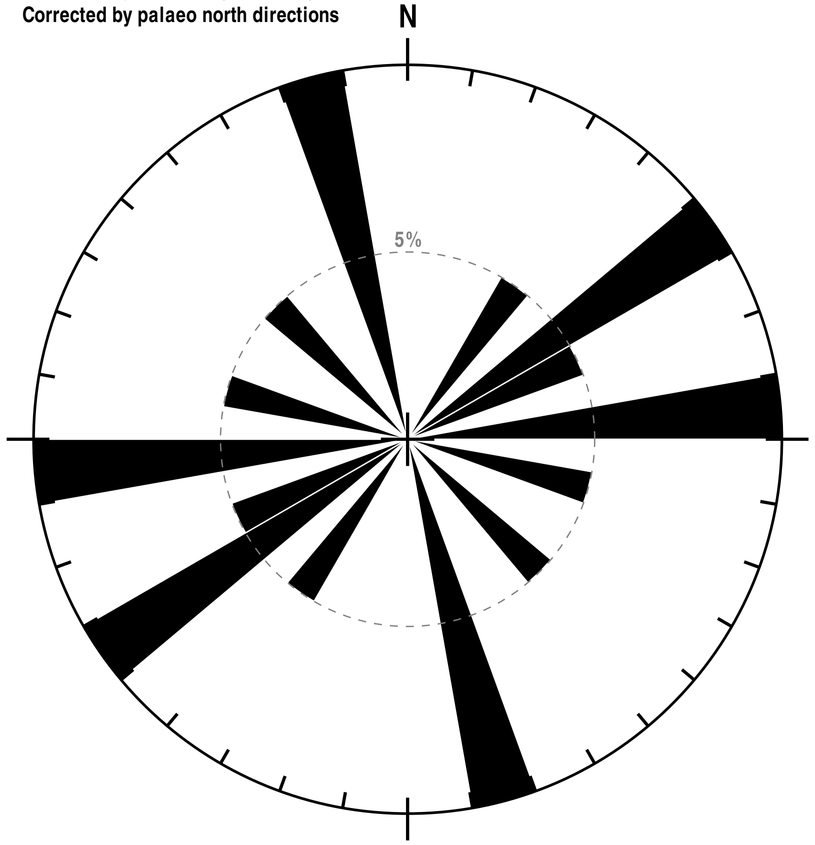

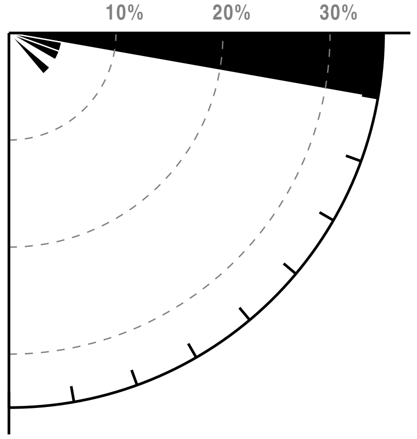

File location: 20240606-134140_DOBI / 5_PS_SEPARATED / USERLINEATION1 / DOBI-OLDAL_USERLINEATION1_.EPS
Plotted by SG2PS (version: Jul 1 2017, 23:50:18) - for reference see www.sg2ps.eu webpage.

USERLINEATION1 FROM LOCATION LENARDDAROC, COLOURED USING GROUP CODE

ORIGINAL

Data number: 17
Average bedding: not measured

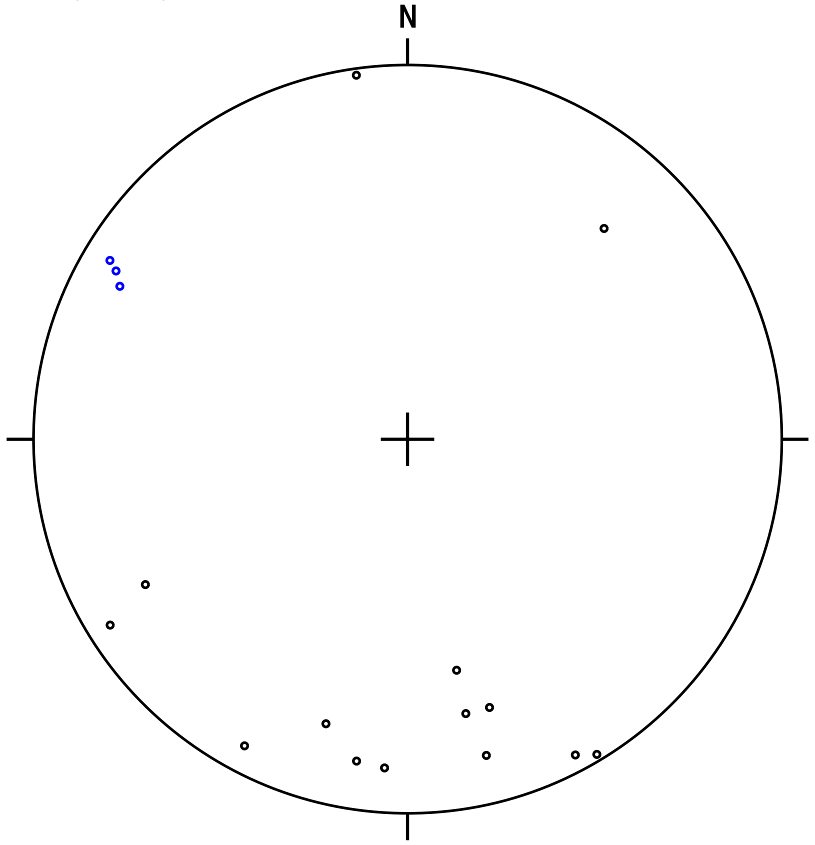

Angelier-plot, Schmidt-net, lower hemisphere

Rose plot for measured data
Average bedding: not measured

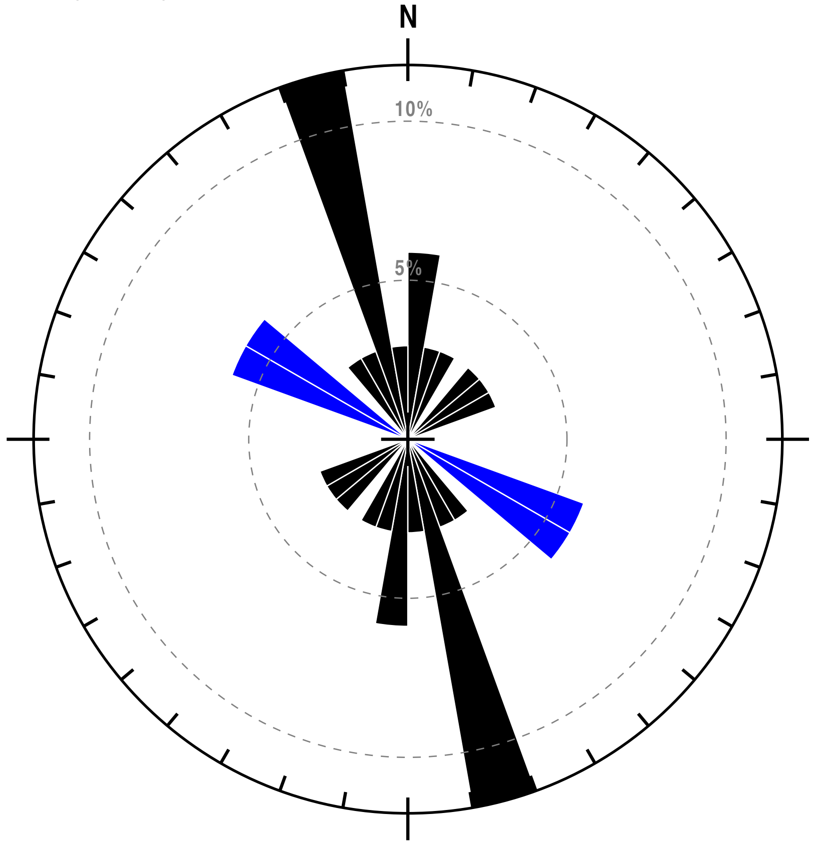

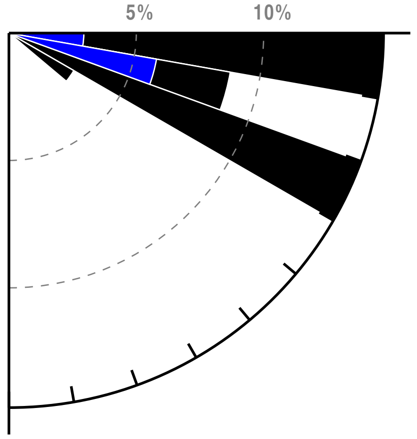

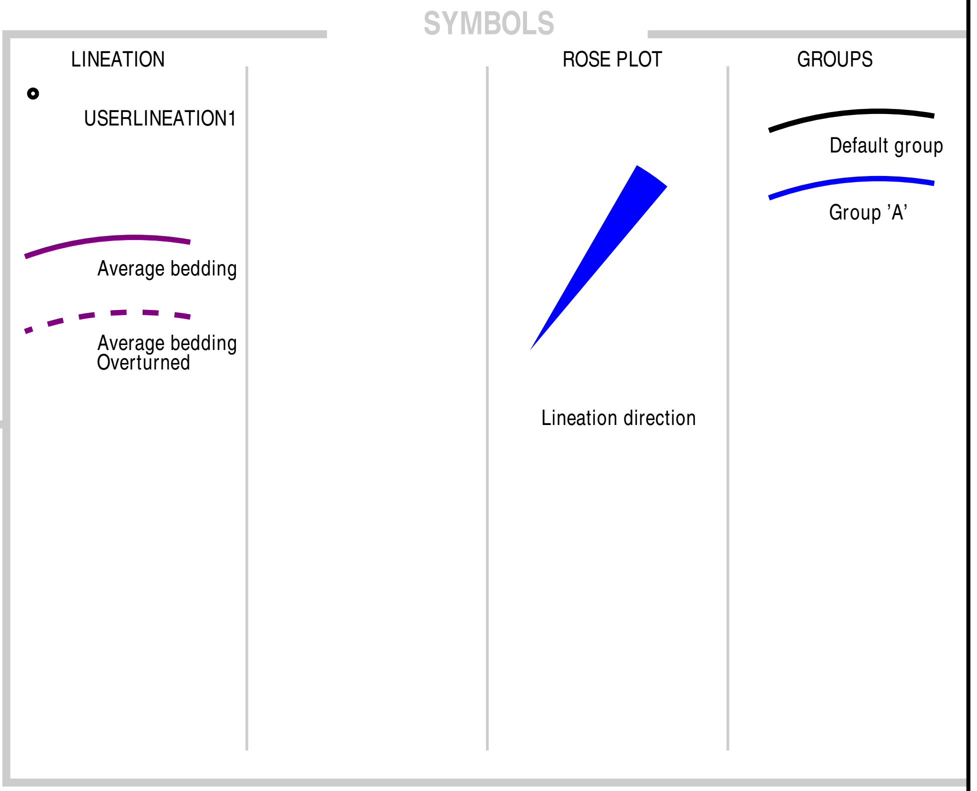

CORRECTED

Data number: 17
Corrected by the average bedding: no bedding measured
Corrected by palaeo north directions

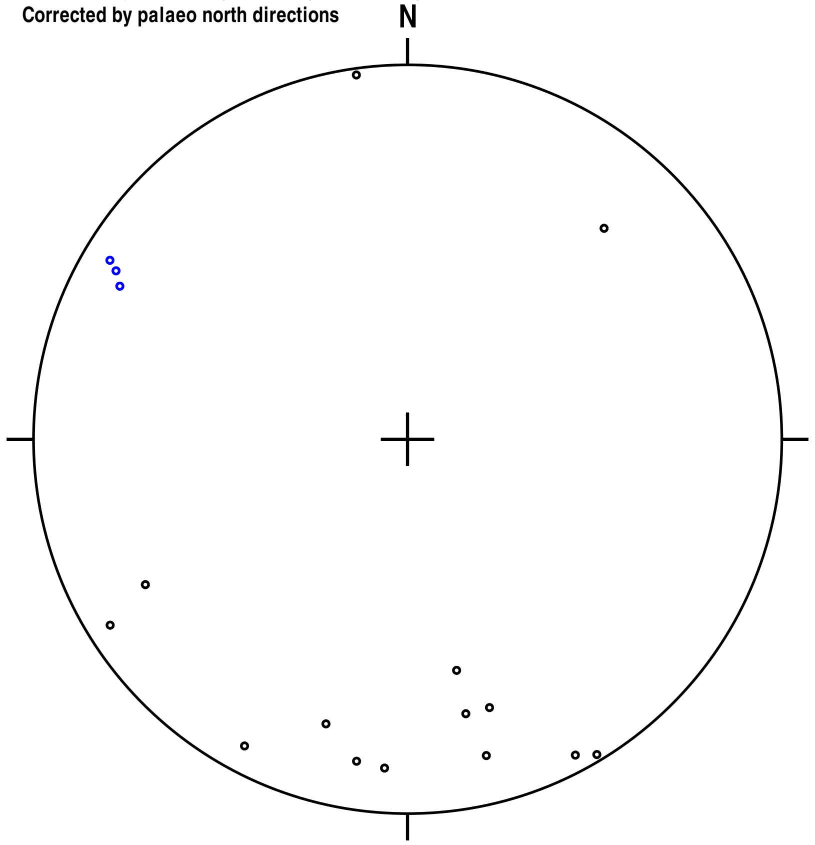

Angelier-plot, Schmidt-net, lower hemisphere

Rose plot for dip corrected data
Corrected by the average bedding: no bedding measured
Corrected by palaeo north directions

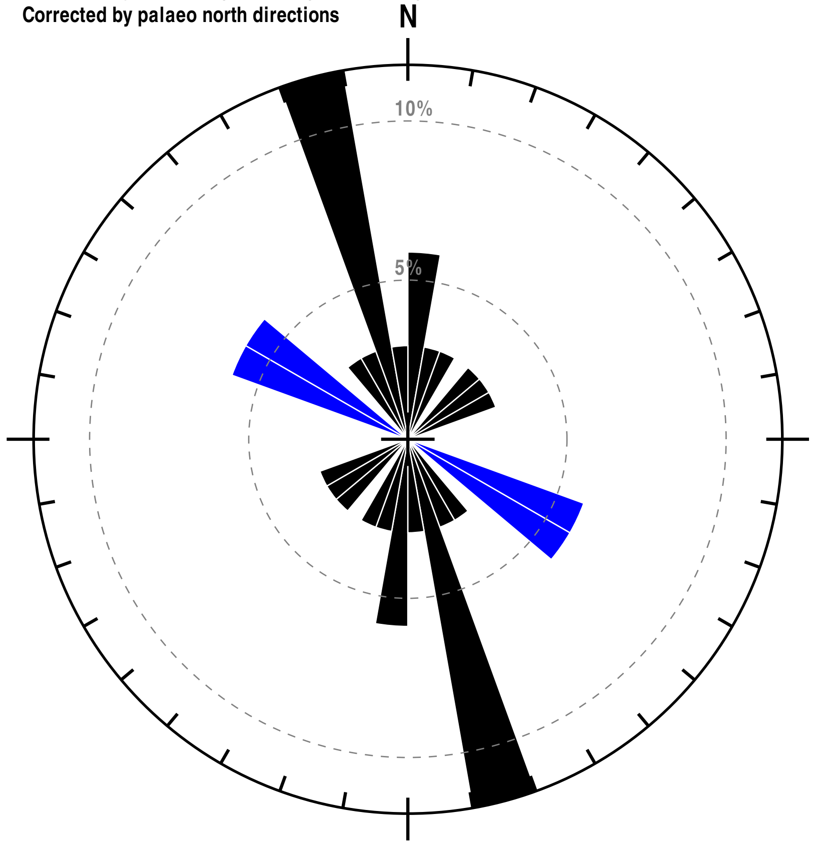

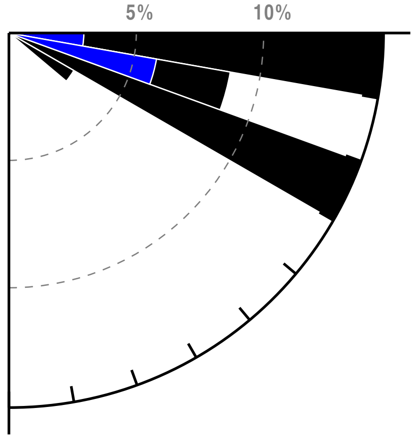

File location: 20240606-134140_DOBI / 5_PS_SEPARATED / USERLINEATION1 / LENARDDAROC_USERLINEATION1_.EPS
Plotted by SG2PS (version: Jul 1 2017, 23:50:18) - for reference see www.sg2ps.eu webpage.

USERLINEATION1 FROM LOCATION SAJOSZENTPETER

Data number: 6
Average bedding: 059/11

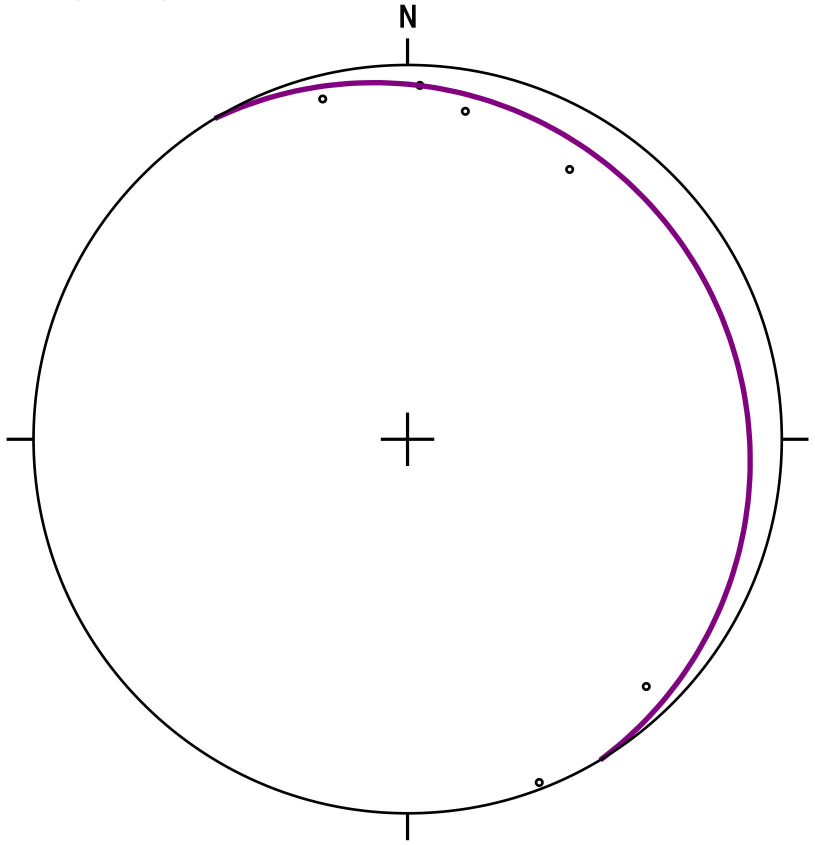

Angelier-plot, Schmidt-net, lower hemisphere

Rose plot for measured data
Average bedding: 059/11

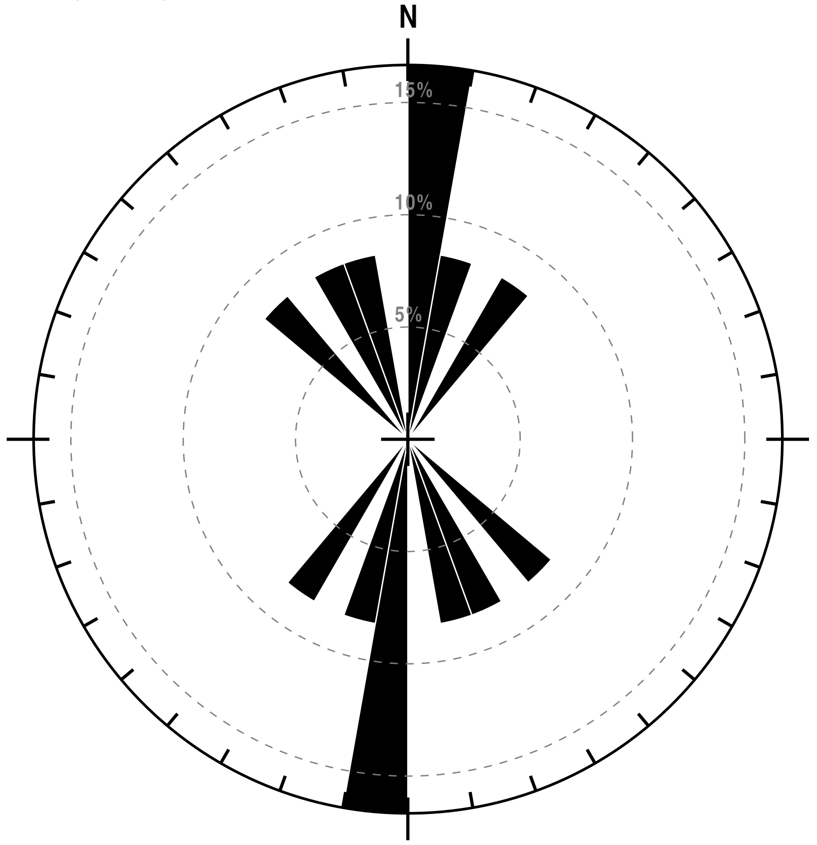

ORIGINAL

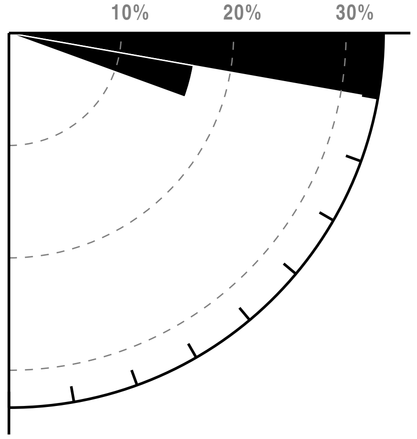

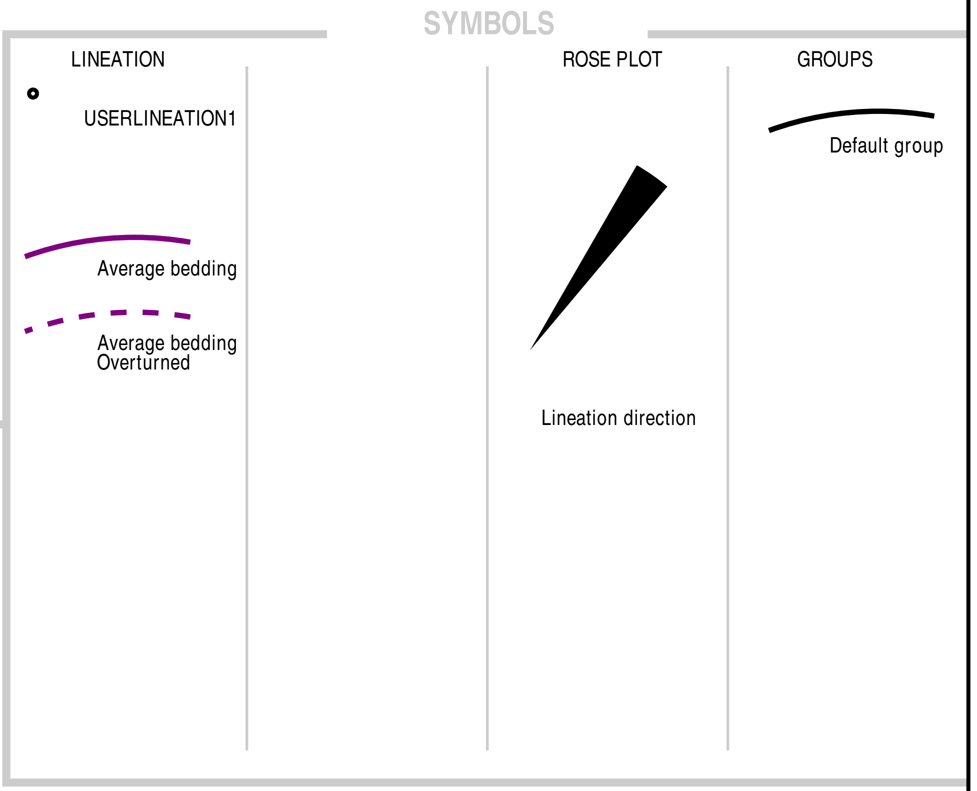

Data number: 6
Corrected by the average bedding: 059/11
Corrected by palaeo north directions

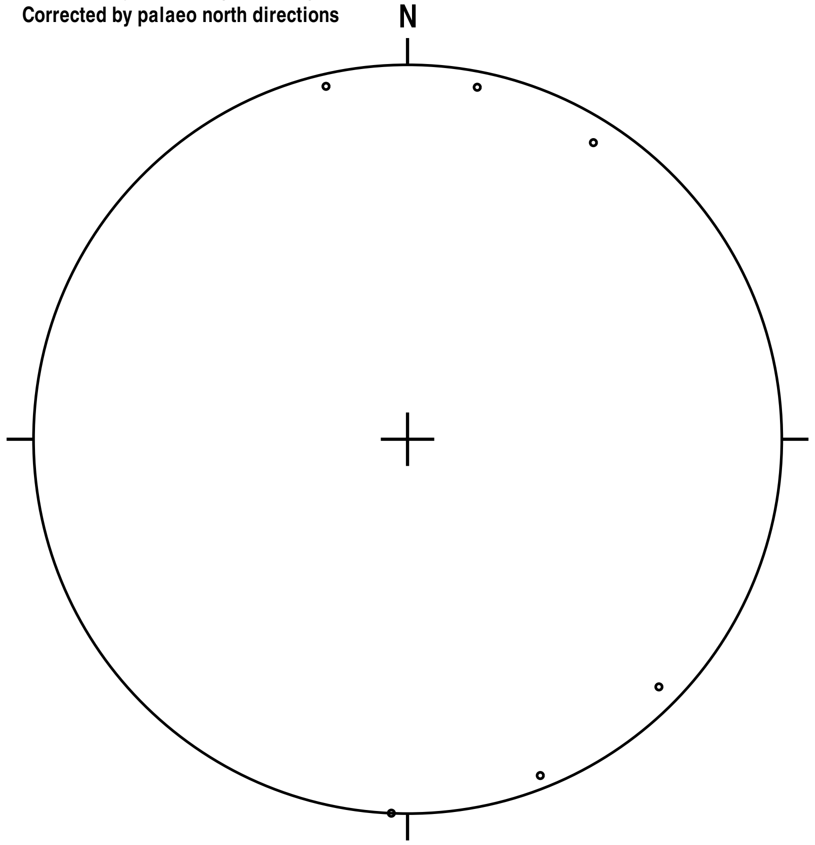

Angelier-plot, Schmidt-net, lower hemisphere

Rose plot for dip corrected data
Corrected by the average bedding: 059/11
Corrected by palaeo north directions

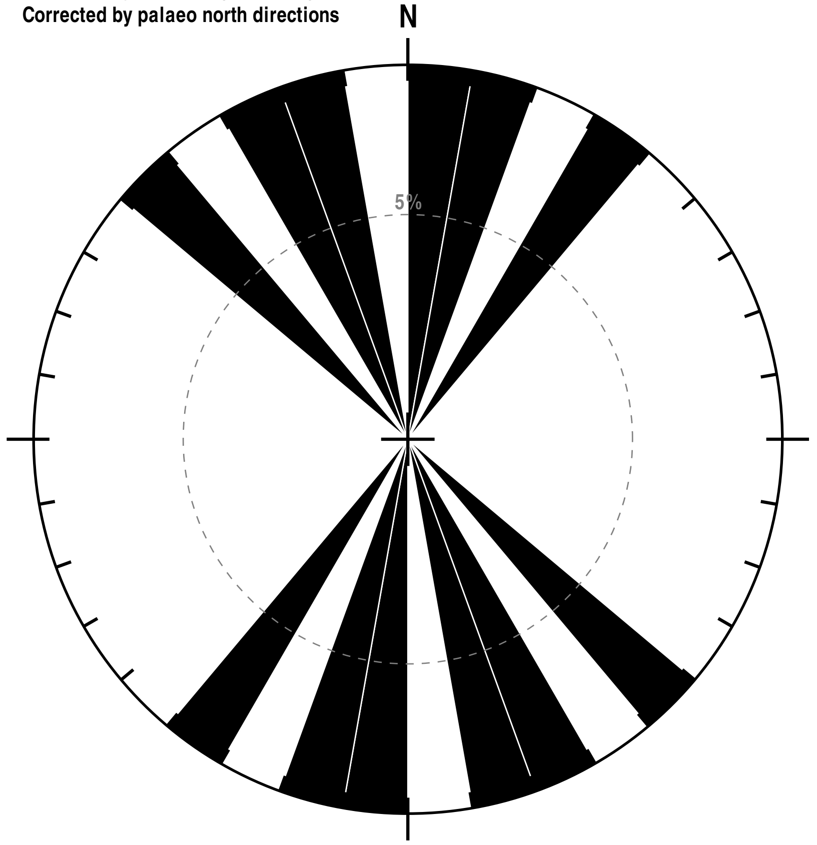

CORRECTED

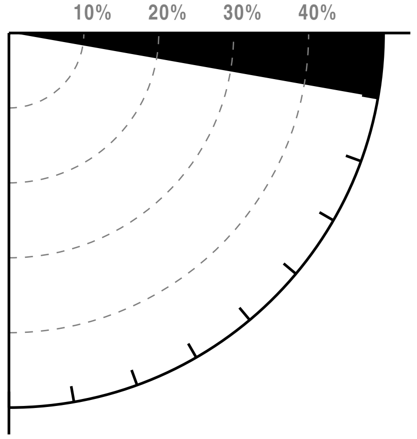

File location: 20240606-134140_DOBI / 5_PS_SEPARATED / USERLINEATION1 / SAJOSZENTPETER_USERLINEATION1_.EPS
Plotted by SG2PS (version: Jul 1 2017, 23:50:18) - for reference see www.sg2ps.eu webpage.
