# Supplementary material for: A 13.06 Ma widespread ignimbrite in the Pannonian Basin captured a snapshot of shallow marine to coastal environment in Central Paratethys
Source: Sci Rep. 2025 Jul 2;15:23528. doi: 10.1038/s41598-025-07002-9 (PMC12223212; doi:10.1038/s41598-025-07002-9)

Site and sample code	*Thin section description*
1. Tarnaszentmária-Dobi cellar<br>TSZM-DOBI	Lapilli tuff with glass shard-rich groundmass.<br>Major components: large quantity of quartz, plagioclase, biotite; pumice clasts and frequent accretionary lapilli. Smaller quantity of sanidine.<br>Accessory components (lithics)*: several metamorphic quartzite, micrite and sparite grains, well-rounded grains of claystone, claystone, meta-siltstone and phyllite. Foraminifera skeleton fragments, both individual (floating) or enclosed in claystone lithics. One andesite lithic clast.
3. Egercsehi<br>EGERCS	Lapilli tuff with glass shard-rich, very fine-grained groundmass with a few small clasts.<br>Major components: biotite, less quartz and plagioclase fragments, very few, small pumices. Considerable amount of sanidine. Accessory components: lots of phyllite, meta-siltstone and less muscovite. A few grains of various volcanic lithics.
4. Lénárddaróc quarry<br>LENARD_ PHREAT_1	Lapilli tuff with glass shard-rich groundmass.<br>Major components: large quantity of quartz, plagioclase, biotite; pumice clasts; a number of accretionary lapilli; smaller quantity of sanidine.<br>Accessory components: large, well rounded meta-claystone containing foraminifera; one siltstone. Three zircon grains.
5. Nagyvisnyó Nvk-3 borehole<br>NVK_3	(thin section made of sand grain mounts). Among calcareous grains of basement rocks, several volcanic resorbed quartz grains appear. One plagioclase grain. Presence of some sanidine grains is questionable but not excluded.
6. Nagyvisnyó Somos valley<br>SOM 532	Tuff with glass shard-rich groundmass.<br>Major components: large quantity of quartz, plagioclase, biotite; pumice clasts; smaller quantity of sanidine.<br>Accessory components: one small, well-rounded claystone.
7. Sajószentpéter cellars:<br>SAJSZP_PIN_F (upper),<br>SAJSZP_PIN_A (lower)	Lapilli tuff with glass shard-rich groundmass.<br>Major components: large quantity of quartz, plagioclase, biotite; pumice clasts; smaller quantity of sanidine.<br>Accessory components: several grains of medium-sized, rounded siltstones.
9. Miskolc-Görömböly cellars	Lapilli tuff with glass shard-rich groundmass.<br>Major components: large quantity of quartz, plagioclase, biotite; pumice clasts; smaller quantity of sanidine.<br>Accessory components: medium-sized, rounded claystones and siltstones. One large muscovite grain.
10. Nyékládháza-1 borehole	Lapilli tuff with glass shard-rich groundmass.<br>Major components: large quantity of quartz, plagioclase, biotite; pumice clasts; less sanidine.<br>Accessory components: large and smaller claystones, silty claystones and one siltstone; two claystone with diatom skeleton remnants. One grain of well-rounded mica schist. A few grains of allanite, garnet and zircon appear as accessories.
12. Mikóháza MIK-1	Lapilli tuff with glass shard-rich groundmass<br>Major components: large quantity of quartz, plagioclase, wavy biotite, and a few sanidine.<br>Accessory components: a number of grains of mica schist and phyllite, muscovite, wavy quartz; one grain of gneiss, microcline, and a composite grain of micrographytic K-feldspare with quartz. One large allanite and one zircon in biotite grain as accessories.
13. Mikóháza road cut<br>(MIK-2)	Lapilli tuff with glass shard-rich groundmass<br>Major components: low quantity of small plagioclase, a few biotite and sanidine grains.<br>Accessory components: well-rounded claystone, wavy quartz and quartzite, mica schist, meta-siltstone, muscovite, and microperthitic orthoclase.
14. Vilyvitány VILY-1<br>(hillslope)	Lapilli tuff with very large grains in strongly albitized and silicified glass shard-rich groundmasss<br>Major components: large quantity of resorbed quartz and partly albitized, perthitic sanidine.<br>Accessory components: a few quartzite, mica schist, muscovite and phyllite. More siltstone and metasandstone. One andesite lithic clast.<br>Elongated large pumices which were affected by strong sericitic, clayey and silicic alteration.

*accessory vs accidental lithics are difficult to distinguish. We consider most of the lithic clast as accessory, having been excavated from the Paleo-Mesozoic substrate.

Cut hand specimens:

1. TSZM-Dobi (Unit 4 ignimbrite)

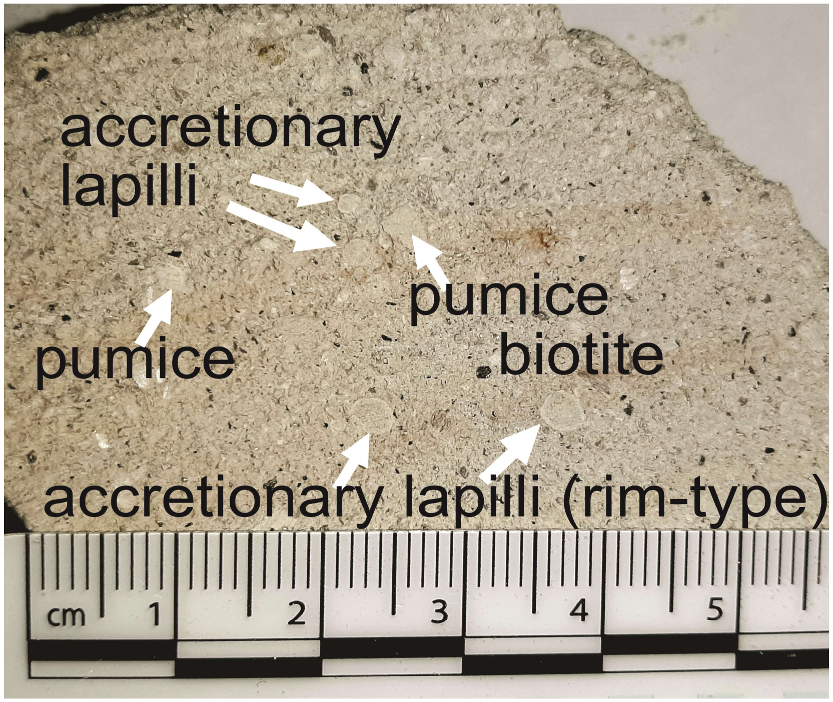

14. Vilyvitány (ignimbrite)

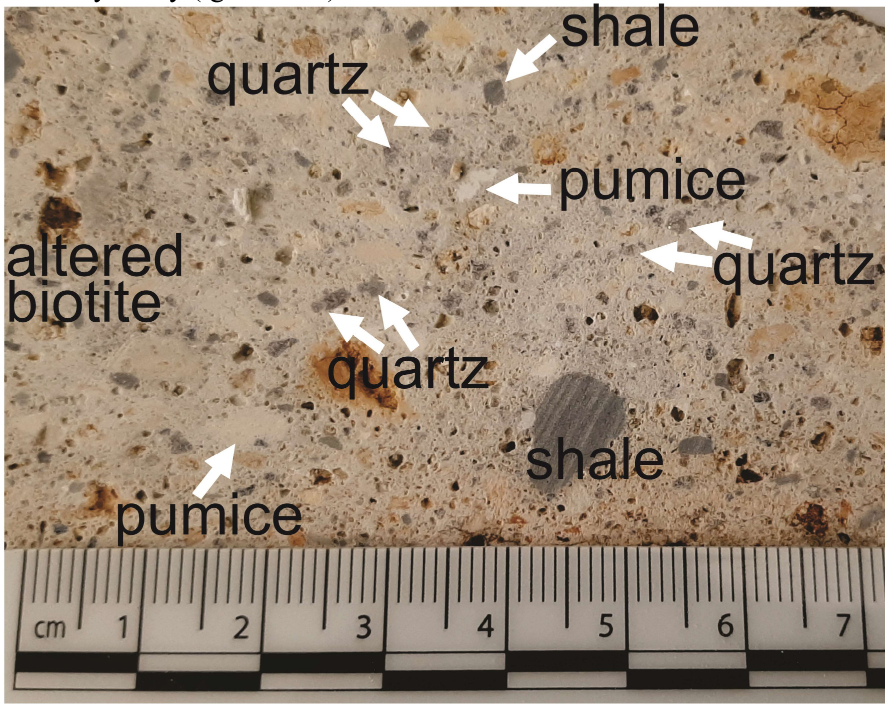

Thin section photomicrographs of TSZM-Dobi Unit 4 ignimbrite

1 N, 4xm (magnification)

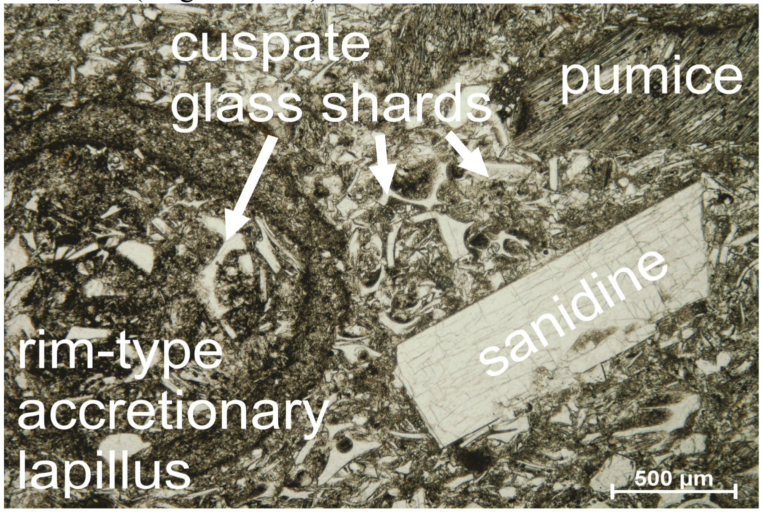

1 N, 10xm

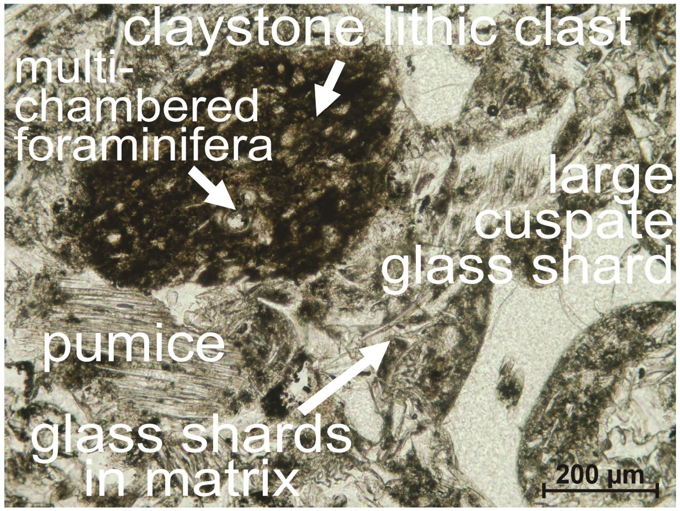

1 N, 4 xm

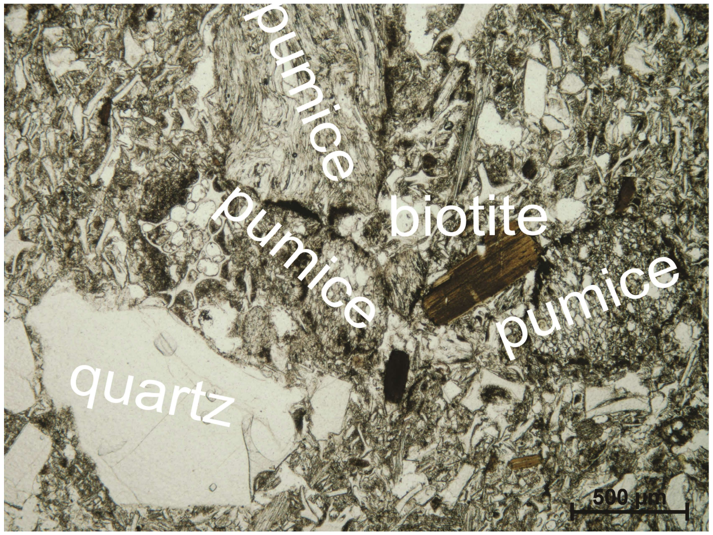

1 N, 20xm

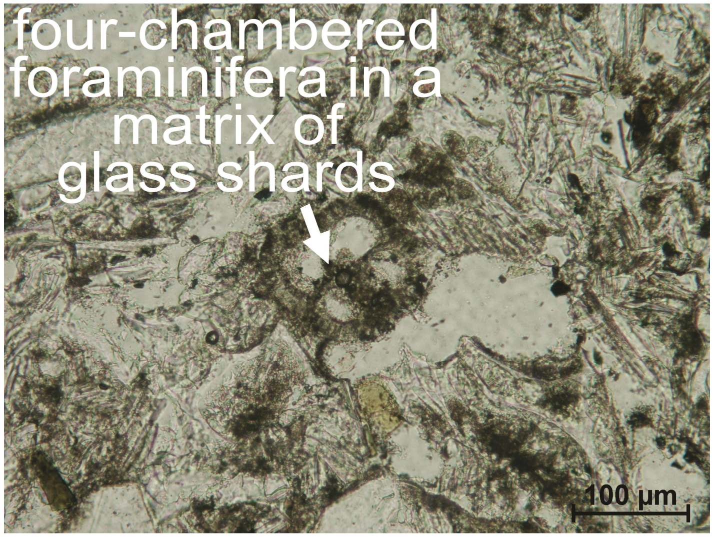

1 N, 10 xm

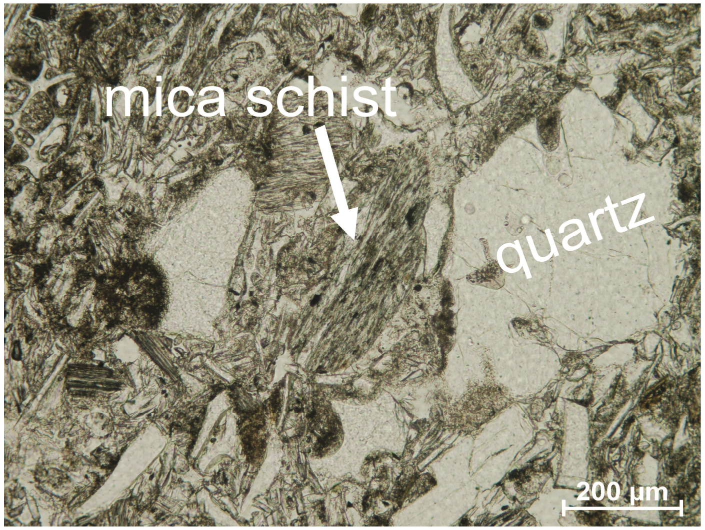

+N, 10xm

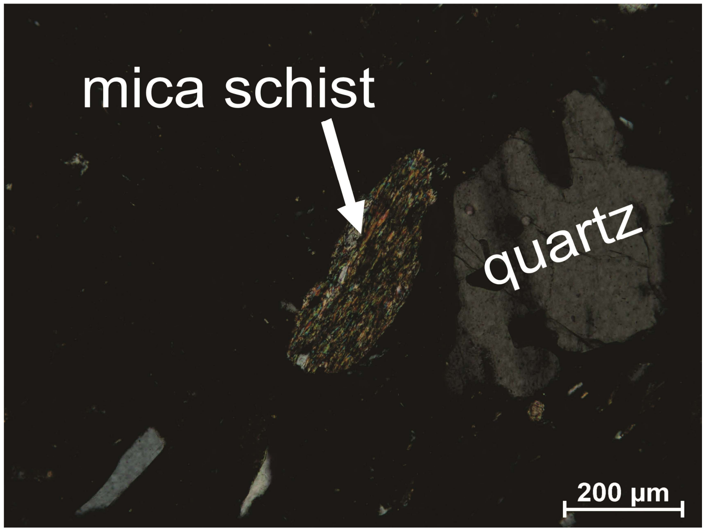

Supplement: Supplementary file 4 — Supplementary Information 4. [file 41598_2025_7002_MOESM4_ESM.pdf]
