# Supplementary material for: A 13.06 Ma widespread ignimbrite in the Pannonian Basin captured a snapshot of shallow marine to coastal environment in Central Paratethys
Source: Sci Rep. 2025 Jul 2;15:23528. doi: 10.1038/s41598-025-07002-9 (PMC12223212; doi:10.1038/s41598-025-07002-9)
Supplement: Supplementary file 6 — Supplementary Information 5B. [file 41598_2025_7002_MOESM6_ESM.pdf]

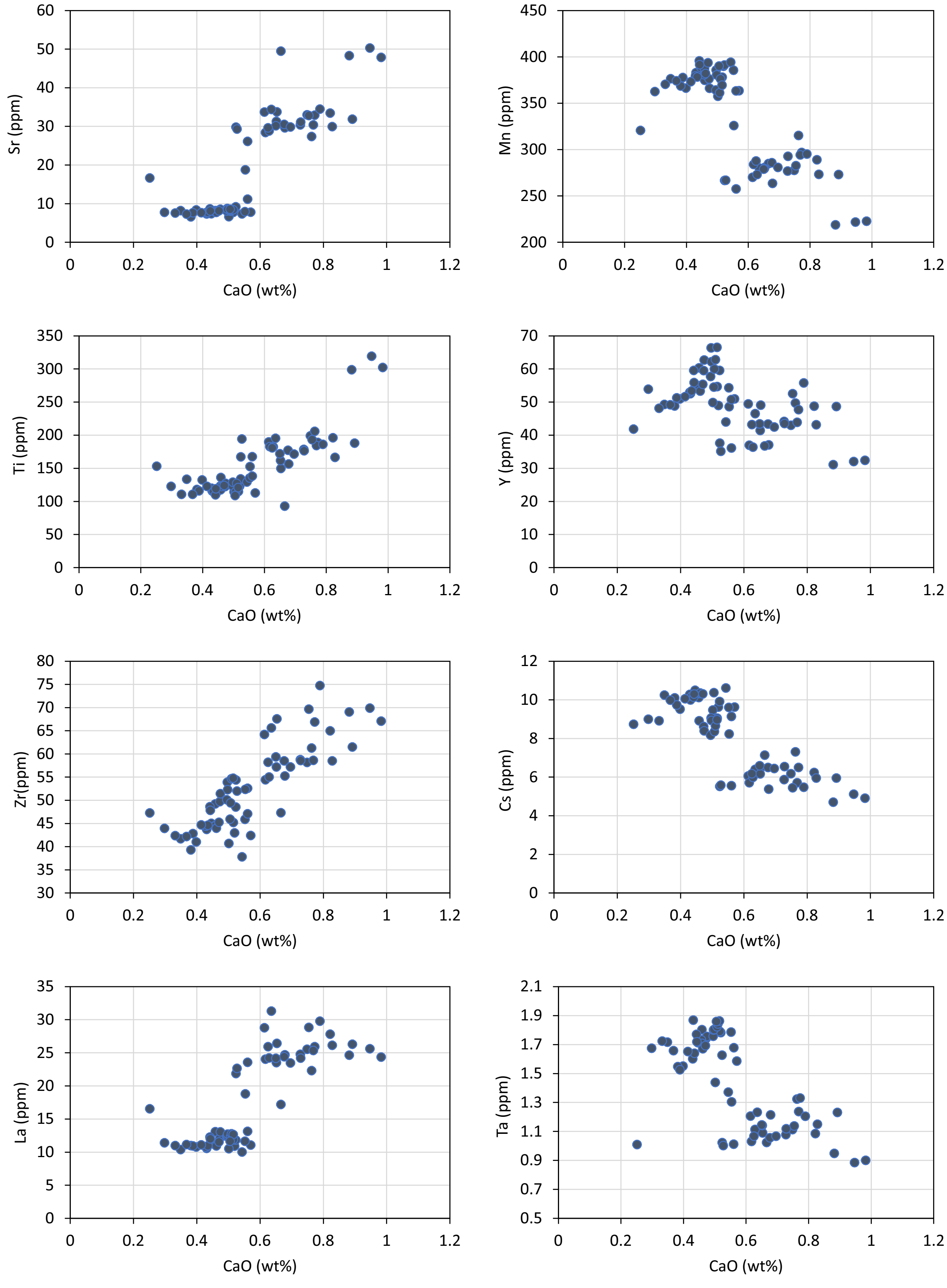

Supplement 5B. Compositional variations of volcanic glass from the Dobi ignimbrites. LA-ICP-MS data.
