# Supplementary material for: A 13.06 Ma widespread ignimbrite in the Pannonian Basin captured a snapshot of shallow marine to coastal environment in Central Paratethys
Source: Sci Rep. 2025 Jul 2;15:23528. doi: 10.1038/s41598-025-07002-9 (PMC12223212; doi:10.1038/s41598-025-07002-9)
Supplement: Supplementary file 7 — Supplementary Information 6. [file 41598_2025_7002_MOESM7_ESM.pdf]

Dobi Ignimbrite volume calculation

The ignimbrite distribution (thickness) is strongly influenced by topography. As a general rule, on steeper slopes there is less, while at the bottom of valleys there is more accumulation, and since the thickness does not depend linearly with distance, volume is a complex task to calculate (Scarpati et al., 2014). Because of a number of uncertainties, there is no single, established method of calculation, but a variety of approximations.

There are many examples of estimates where ignimbrite distribution was treated as radial and an average thickness value was taken (Morgan et al., 1984; Ratté et al., 1984; Henry and Price, 1984; Sparks et al., 1985; Hildreth and Mahood, 1986; Rose and Chesner, 1987; Gardeweg and Ramirez, 1987; Chesner and Rose, 1991; Nairn et al., 1994; Le Pennec et al., 1994; Lipman, 2000; Linsday et al., 2001; Christiansen, 2001; Ort, 1993; Moràn-Zenteno et al., 2004; Soler et al., 2007; Lipman and McIntosh, 2008; Salisbury et al., 2010; Willcock et al., 2013), sometimes with maximum thickness at the centre and zero volume at the perimeter (Fisher et al., 1993; Civetta et al., 1997). However, these methods cannot model the above-mentioned inequalities: approximating the ignimbrite morphology to a regular geometric shape seems to be a simplification that introduces significant error and may also severely underestimate the actual extent of the ignimbrite sheet. On the other hand, they have the advantage of being able to deal with a limited amount of data available. By including several parameters, e.g. ignimbrite crystal concentration (Walker, 1972) or meteorological parameters (atmospheric circulation and wind profile), the calculation method can be made more accurate (e.g. for the Campanian Ignimbrite: Costa et al., 2012).

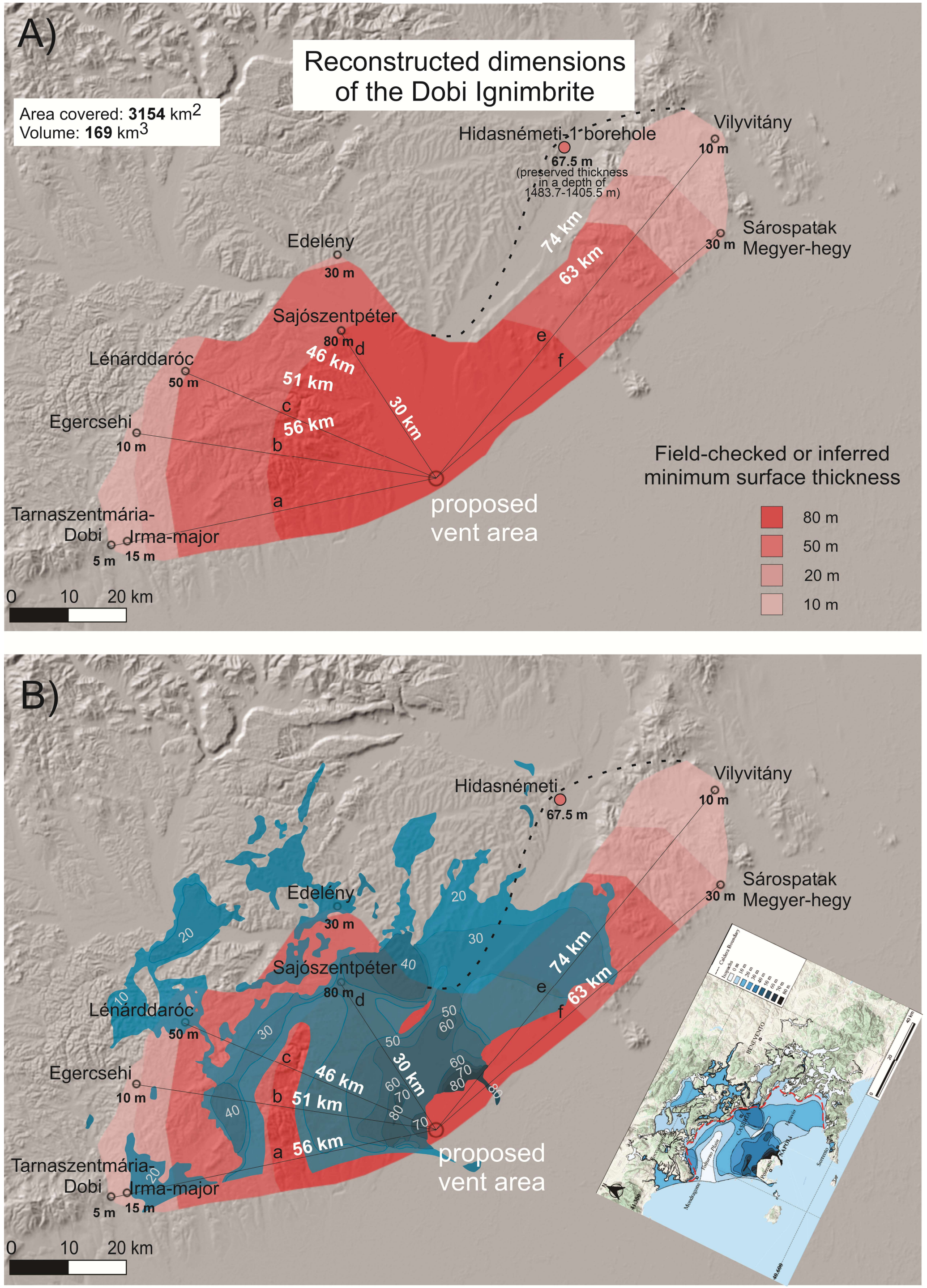

Suppl. 6, Fig. 1. Dimensions of the Dobi Ignimbrite. **A:** Reconstructed areal distribution with observed/inferred thickness (surface) values. **B:** For comparison, the Campanian Ignimbrite with apparently similar areal extent is shown (legend in blue displays field-based isopach data after Silleni et al., 2020)[39].

In this research, at first, due to the limited number of observed/inferred thickness data (**Suppl. 6, Fig. 1**), a simpler approach based on thickness data and areal distribution was applied:

$V=A_1\times h_1 + A_2\times h_2 + A_3\times h_3 + A_4\times h_4$

Where:

V is the volume,

$A_{1\text{-}4}$ are the areas covered by the different-thickness ignimbrite,

$h_{1\text{-}4}$ are the average thickness of the ignimbrite deposit.

The calculations were carried out in open-source software of QGIS: the thickness values found in specific outcrops were interpolated, and the assumed extension boundary and eruption center were determined. The QGIS vector Geoprocessing Buffer Tool was used to define concentric boundaries and the extent of the areas thus defined was also determined using this software. The resulting minimum estimate is 170 km^3.

We can estimate the maximum value if we treat all areas as half-truncated cones. In our study, there are eight known thickness values, and in each case we calculated with the radius farthest from the center, so the volume can be described with three truncated cones and a cylinder, and, finally, halving the value.

$V= \Sigma\, h_i*\pi*(R_i^2+R_i*r_i+r_i^2)/3$

Where:

V is the volume,

h_i are the average thickness of the ignimbrite deposit,

R_i is the base circle for each cone (R_{max} is the last, largest one),

r_i is the cover circle for each cone. After halving the value, the volume is 370 km^3, (**Suppl. 6, Figure 2**) detailed as follows:

Section with min – max thickness	Thickness (in km)	$R_{outside}$ (in km)	R_{inside} (in km)	Volume of each truncated cone (in km^3)
4 (00 – 10)	0.01	74	74	172
3 (10 – 20)	0.01	74	63	148
2 (20 – 50)	0.03	63	46	282

1 (50 – 80)	0.03	46	30	138
		Total (**NOT HALVED**)		740
		Halved total		370

Possibly, the most realistic calculation can be the application of decreasing thickness: i.e., a constant thickness of 80 m in the inner part and decreasing thickness values for each area. Integrating the volume from this thickness behaviour, a volume of ~200 km^3 can be calculated **(Supp. 6, Fig. 2).**

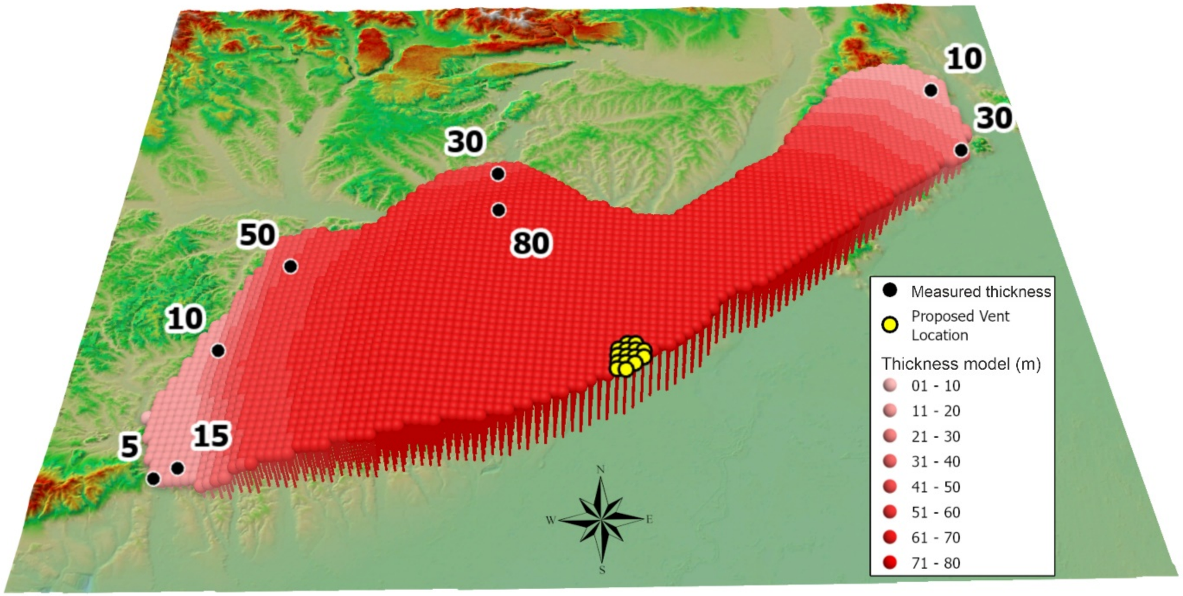

Suppl. 6, Fig. 2. *Application of decreasing thickness behavior from 80 m in the centre*

Finally, the minimum (black) and maximum (green) calculation methods is illustrated in **Suppl. 6, Fig. 3**

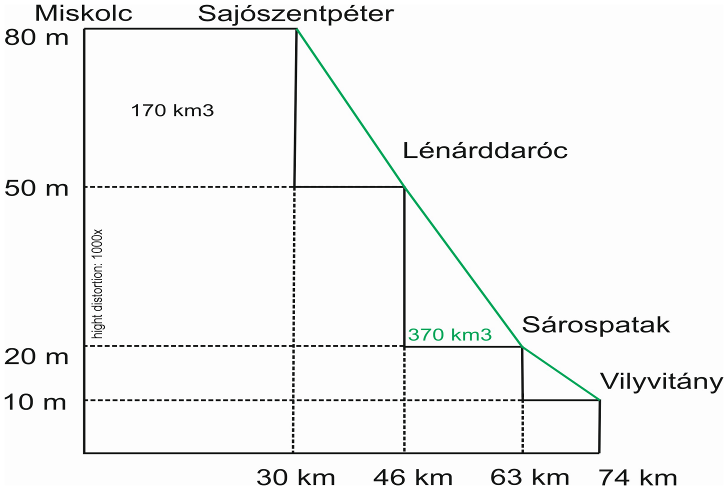

References for Supplement 6:

(1) Scarpati, C., Sparice, D. & Perrotta, A. A crystal concentration method for calculating ignimbrite volume from distal ash-fall deposits and a reappraisal of the magnitude of the Campanian Ignimbrite. Journal of Volcanology and Geothermal Research, 280, 67-75 (2014)

(2) Morgan, L. A., Doherty, D.J. & Leeman, W.P. Ignimbrites of the eastern Snake River plain: evidence from major caldera-forming eruptions. J. Geophys. Res. 89 (B10), 8665-8678 (1984)

(3) Ratté, J.C., Marvin, R.F. & Naeser, C.W., Calderas and ash flow tuffs of the Mogollon Mountains, southwestern New Mexico. J. Geophys. Res. 89 (B10), 8713-8732 (1984)

(4) Henry, C.D., Price, J.G. Variation in caldera development in the Tertiary volcanic field of Trans-Pecos, Texas. J. Geophys. Res. 89 (B10), 8765-8786 (1984)

(5) Sparks, R.S.J., Francis, P.W., Hamer, R.D., Pankhurst, R.J., O'Callaghan, L.O., Thorpe, R.S. & Page, R. Ignimbrites of the Cerro Galan Caldera, NW Argentina. J. Volcanol. Geotherm. Res. 24, 205-248 (1985)
(6) Hildreth, W., Mahood, G.A. Ring-fracture eruption of the Bishop Tuff. Bull. Geol. Soc. Am. 97, 396-403 (1986)
(7) Rose, W.I., Chesner, C.A. Dispersal of ash in the great Toba eruption 75 ka. Geology 15, 913-917 (1987)
(8) Gardeweg, M., Ramìrez, C.F. La Pacana caldera and the Atana Ignimbrite — a major ash-flow and resurgent caldera complex in the Andes of northern Chile. Bull. Volcanol. 49, 547-566 (1987)
(9) Chesner, C.A., Rose, W.I. Stratigraphy of the Toba Tuffs and the evolution of the Toba Caldera Complex, Sumatra, Indonesia. Bull. Volcanol. 53, 343-356 (1991)
(10) Nairn, I.A., Wood, C.P. & Bailey, R.A. The Reporoa caldera, Taupo Volcanic Zone: source of the Kaingaroa Ignimbrites. Bull. Volcanol. 56, 529-537 (1994)
(11) Le Pennec, J.L., Bourdier, J.L., Froger, J.L., Temel, A., Camus, G. & Gourgaud, A. Neogene ignimbrites of the Nevsehir plateau (central Turkey): stratigraphy, distribution and source constrains. J. Volcanol. Geoth. Res. 63, 59-87 (1994)
(12) Lipman, P.W. Central San Juan caldera cluster: regional volcanic framework. Geol. Soc. Am. Spec. Pap. 69, 9-69 (2000)
(13) Lindsay, J.M., de Silva, S., Trumbull, R., Emmermann, R. & Wemmer, K. La Pacana caldera, N. Chile: a re-evaluation of the stratigraphy and volcanology of one of the world's largest resurgent calderas. J. Volcanol. Geotherm. Res. 106, 145-173 (2001)
(14) Christiansen, R.L. The Quaternary and Pliocene Yellowstone plateau volcanic field of Wyoming, Idaho and Montana. USGS Professional Paper 727-G (2001)
(15) Ort, M.H. Eruptive processes and caldera forming in a nested downsag-collapse caldera: Cerro Panizos, central Andes Mountains. J. Volcanol. Geotherm. Res. 56, 221-252 (1993)
(16) Moràn-Zenteno, D.J., Alba-Aldave, L.A., Solè, J. & Iriondo, A. A major resurgent caldera in southern Mexico: the source of the late Eocene Tizapotla ignimbrite. J. Volcanol. Geotherm. Res. 136, 97-119 (2004)
(17) Soler, M.M., Caffe, P.J., Coira, B.L., Onoe, A.T. & Mahlburg Kay, S. Geology of the Vilama caldera: a new interpretation of a large-scale explosive event in the Central Andean plateau during the Upper Miocene. J. Volcanol. Geotherm. Res. 164, 27-53 (2007)
(18) Lipman, P.W., McIntosh, W.C. Eruptive and noneruptive calderas, northeastern San Juan Mountains, Colorado: where did the ignimbrites come from?. Bull. Geol. Soc. Am. 120, 771-795 (2008)
(19) Salisbury, M.J., Jicha, B.R., de Silva, S.L., Singer, B.S., Jiménez, N.C. & Ort, M.H. 40Ar/39Ar chronostratigraphy of the Altiplano-Puna volcanic complex ignimbrites reveals the developments of a major magmatic province. Bull. Geol. Soc. Am. 123, 821-840 (2010)
(20) Willcock, M.A.W., Cas, R.A.F., Giordano, G. & Morelli, C. The eruption, pyroclastic flow behaviour, and caldera in-filling processes of the extremely large volume (> 1290 km3), intra- to extra-caldera, Permian Ora (Ignimbrite) Formation, Southern Alps, Italy. J. Volcanol. Geotherm. Res. 265, 102-126 (2013)

(21) Fisher, R.V., Orsi, G., Ort, M. & Heiken, G. Mobility of a large volume pyroclastic flow — emplacement of the Campanian Ignimbrite, Italy. J. Volcanol. Geotherm. Res. 56, 205-220 (1993)

(22) Civetta, L., Orsi, G., Pappalardo, L., Fischer, R.V., Heiken, G., & Ort, M. Geochemical zoning, mingling, eruptive dynamics and depositional processes — the Campanian Ignimbrite, Campi Flegrei caldera, Italy. J. Volcanol. Geotherm. Res. 75, 183-219 (1997)

(23) Walker, G.P.L. Crystal concentration in ignimbrites. Contrib. Mineral. Petrol. 36, 135-146 (1972)

(24) Costa, A., Folch, A., Macedonio, G., Giaccio, B., Isaia, R. & Smith, V.C. Quantifying volcanic ash dispersal and impact of the Campanian Ignimbrite super eruption. Geophys. Res. Lett. 39 (10), (2012)
