# Supplementary material for: A 13.06 Ma widespread ignimbrite in the Pannonian Basin captured a snapshot of shallow marine to coastal environment in Central Paratethys
Source: Sci Rep. 2025 Jul 2;15:23528. doi: 10.1038/s41598-025-07002-9 (PMC12223212; doi:10.1038/s41598-025-07002-9)
Supplement: Supplementary file 8 — Supplementary Information 7. [file 41598_2025_7002_MOESM8_ESM.pdf]

The analyses involved ablation of minerals with a Resonetics M-50 laser system operating at a wavelength of 193 nm. Spot diameters of 33 µm, repetition rates of 3 Hz and fluence of 2.0 J/cm^2 resulted in a spot depth of 10 µm. The ablated material was carried into helium and then mixed with nitrogen (Paquette et al. 2014) and argon before injection into the plasma source of a Thermo Element XR Sector Field high-resolution ICP-MS equipped with the jet interface pumping device. The alignment of the instrument and mass calibration were performed before every analytical session using the NIST SRM 612 reference glass, by inspecting the signals of ^{238}U, ^{232}Th and ^{208}Pb and by minimising the ThO^+/Th^+ ratio. The analytical method for isotope dating with laser ablation ICP-MS is basically similar to that reported in Hurai et al. (2010) and Paquette et al. (2019). The ^{235}U signal is calculated from ^{238}U based on the ratio $^{238}U/^{235}U$ = 137.818 (Hiess et al. 2012). Single analyses consisted of 30 seconds of background integration with laser off followed by 60 seconds integration with the laser firing and a 30 seconds delay to wash out the previous sample and prepare the next analysis.

Data were corrected for U-Pb fractionation occurring during laser sampling and for instrumental mass bias by standard bracketing with repeated measurements of GJ-1 zircon primary standard (Jackson et al. 2004). Repeated analyses of 91500 zircon reference material (Wiedenbeck et al. 1995) during each analytical session and treated as unknown, independently control the reproducibility and accuracy of the corrections. Data reduction was carried out with the software package GLITTER® from Macquarie Research Ltd (van Achterbergh et al. 2001; Jackson et al. 2004).

Schärer (1984) demonstrated that most zircons are affected during their growth by deficits and excesses of both ^{230}Th and ^{231}Pa relative to the initial Th/U secular equilibrium. In order to obtain accurate crystallization ages, it is necessary to correct for the effect of initial disequilibria caused by intermediate nuclides in the ^{238}U and ^{235}U decay series. Sakata et al. (2017) and Sakata (2018) propose a simplified correction model and a related Microsoft Excel spreadsheet based on the mathematical framework of Wendt and Carl (1985) using the following equations:

$$^{206}Pb^*/^{238}U = (e^{l238t} - 1) + l_{238}/l_{230}\,(f_{Th/U} - 1)\,(1 - e^{-l230t})\,e^{l238t}$$

$$^{207}Pb^*/^{235}U = (e^{l235t} - 1) + l_{235}/l_{231}\,(f_{Pa/U} - 1)\,(1 - e^{-l231t})\,e^{l235t}$$

The concentrations in U-Th-Pb are calibrated relative to the certified contents of GJ-1 zircon (Jackson et al. 2004) reference material. The isotopic ratios and 2s level uncertainties were corrected from elemental and isotopic fractionation, as well as Th/U disequilibria. A systematic external error is subsequently propagated by quadratic addition of uncertainties on U and Th decay constants as well as of the variability of the primary reference material used for corrections (GJ-1) and of the long-term variability of the secondary reference material (91500). According to Horstwood et al. (2016), this systematic external error is added to the 2s error associated to the weighted mean $^{206}Pb/^{238}U$ ages calculation. The Tera and Wasserburg (1972) diagrams were generated using Isoplot/Ex v. 2.49 software package by Ludwig (2001).

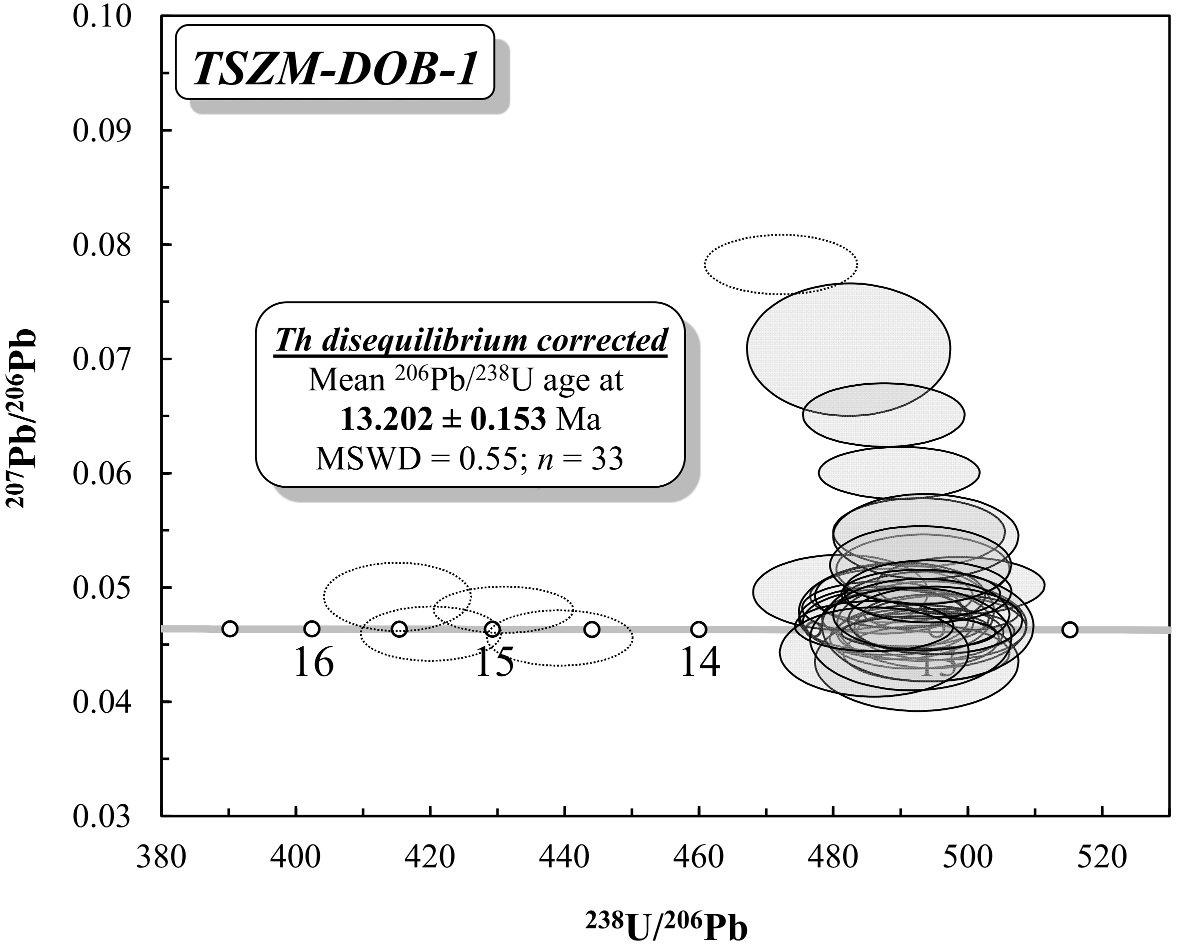

TSZM-DOB-1
Th disequilibrium corrected
Mean 206Pb/238U age at
13.202 ± 0.153 Ma
MSWD = 0.55; n = 33
0.10
0.09
0.08
0.07
0.06
0.05
0.04
0.03
16
15
14
13
380
400
420
440
460
480
500
520
207Pb/206Pb
238U/206Pb

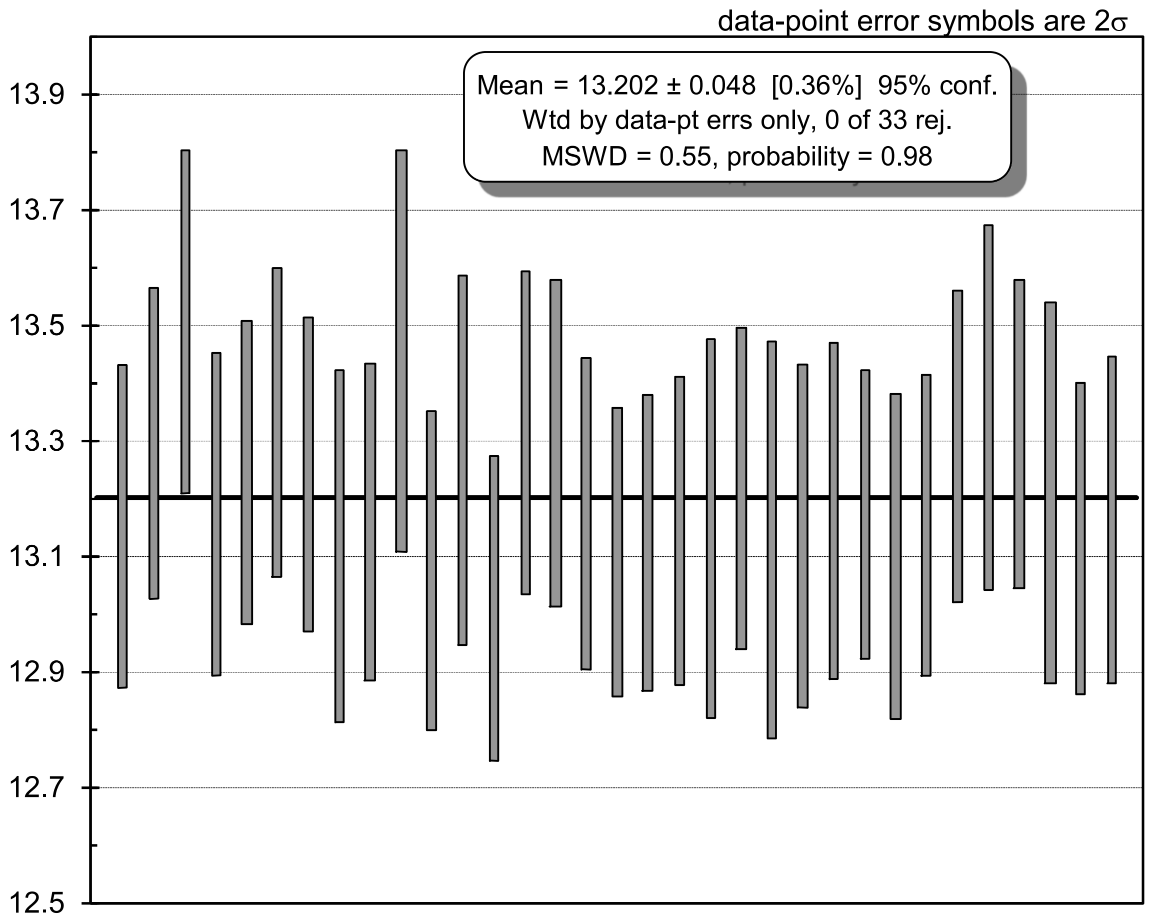

data-point error symbols are 2σ
Mean = 13.202 ± 0.048 [0.36%] 95% conf.
Wtd by data-pt errs only, 0 of 33 rej.
MSWD = 0.55, probability = 0.98
13.9
13.7
13.5
13.3
13.1
12.9
12.7
12.5

GLITTER4.4.2: Laser Ablation Analysis Results

C:\Documents and Settings\oper\Bureau\150421a\150421a.FIN
Created: Fri Apr 16 12:06:43 2021

GLITTER!: Isotope ratios.

Analysis_#	Pb207/Pb20(	Pb206/U238	Pb207/U235	Pb208/Th232
Zr1	0.06011	0.09742	0.80759	0.03068
Zr2	0.06053	0.09706	0.81024	0.03022
Zr3	0.05991	0.09756	0.80604	0.03018
Zr4	0.06009	0.09765	0.80914	0.02955
Zr5	0.07419	0.17962	1.83763	0.0542
Zr6	0.07509	0.17946	1.85839	0.05362
Zr7	0.05125	0.00203	0.01435	0.00067
Zr8	0.04895	0.00205	0.01383	0.00068
Zr9	0.04923	0.00208	0.01413	0.0007
Zr10	0.05445	0.00203	0.01526	0.00067
Zr11	0.04927	0.00211	0.01432	0.00073
Zr12	0.04208	0.0021	0.01221	0.00064
Zr13	0.0603	0.09761	0.81169	0.03036
Zr14	0.05991	0.09769	0.80699	0.02936
Zr15	0.04247	0.00208	0.0122	0.0007
Zr16	0.0597	0.00204	0.01683	0.00077
Zr17	0.04786	0.00205	0.01356	0.00065
Zr18	0.04699	0.00204	0.01322	0.00071
Zr19	0.04636	0.00202	0.01294	0.00065
Zr20	0.04512	0.00203	0.01266	0.00065
Zr21	0.07055	0.00208	0.0202	0.0008
Zr22	0.04607	0.00202	0.01283	0.00064
Zr23	0.05961	0.09762	0.80243	0.03035
Zr24	0.06015	0.09769	0.81034	0.03078
Zr25	0.04592	0.0022	0.01394	0.00072
Zr26	0.04751	0.00205	0.01341	0.00063
Zr27	0.04982	0.00201	0.01379	0.00068
Zr28	0.04688	0.00205	0.01328	0.00066
Zr29	0.03058	0.00219	0.00923	0.00066
Zr30	0.0466	0.00205	0.01318	0.00065
Zr31	0.0462	0.00205	0.01307	0.00065
Zr32	0.04449	0.0021	0.01291	0.00074
Zr33	0.0606	0.0971	0.81135	0.02968
Zr34	0.06018	0.09762	0.81011	0.03084
Zr35	0.04561	0.00203	0.01278	0.00063
Zr36	0.07783	0.01848	0.19831	0.04505
Zr37	0.07806	0.00212	0.02283	0.00145
Zr38	0.04395	0.00202	0.01227	0.00067
Zr39	0.04738	0.00202	0.01319	0.00067
Zr40	0.04522	0.00228	0.0142	0.00073
Zr41	0.05088	0.00212	0.01486	0.00082
Zr42	0.02739	0.00191	0.00721	0.00165
Zr43	0.0602	0.09762	0.81036	0.02985
Zr44	0.06084	0.09679	0.81202	0.03014
Zr45	0.04579	0.00202	0.01277	0.00065
Zr46	0.04385	0.00203	0.01226	0.00065
Zr47	0.04311	0.00203	0.01209	0.00067
Zr48	0.04896	0.00204	0.01379	0.00065
Zr49	0.07441	0.17956	1.84234	0.05323
Zr50	0.07373	0.18017	1.83188	0.0535
Zr51	0.05968	0.09771	0.80421	0.03031
Zr52	0.05984	0.09775	0.80658	0.02999

GLITTER!: Isotopic ratios: 1 sigma uncertainty.

Analysis_#	Pb207/Pb20(	Pb206/U238	Pb207/U235	Pb208/Th232
Zr1	0.0007	0.0009	0.00871	0.0005
Zr2	0.0007	0.00089	0.00865	0.00048
Zr3	0.00068	0.0009	0.00843	0.00046
Zr4	0.00069	0.0009	0.00858	0.00047
Zr5	0.00086	0.00166	0.01967	0.00061
Zr6	0.00086	0.00165	0.01962	0.0006
Zr7	0.00138	0.00002	0.00037	0.00001
Zr8	0.00112	0.00002	0.00031	0.00001
Zr9	0.0015	0.00002	0.00042	0.00001
Zr10	0.00141	0.00002	0.00038	0.00001
Zr11	0.00202	0.00003	0.00057	0.00002
Zr12	0.00177	0.00003	0.0005	0.00001
Zr13	0.00067	0.00089	0.00836	0.00044
Zr14	0.00067	0.00089	0.00833	0.00043
Zr15	0.00186	0.00003	0.00052	0.00002
Zr16	0.00117	0.00002	0.00032	0.00001
Zr17	0.00108	0.00002	0.0003	0.00001
Zr18	0.00118	0.00002	0.00032	0.00001
Zr19	0.00168	0.00002	0.00046	0.00001
Zr20	0.0012	0.00002	0.00033	0.00001

HUNGARY-TSZM-DOB-1

Analysis_#	Sample	Pb ppm	Th ppm	U ppm	Th/U	f min	Uncertainty	Pb207/U235	2 sigma error Pb207/U235	Pb206/U238	2 sigma error Pb206/U238	Rho	Age (Ma) Pb206/U238	2 sigma error Pb206/U238
Zr7 150421a	A01 centre	1.2	440	489	0.90	0.2997	0.1498	0.01435	0.00075	0.002030	0.000043	0.41	13.149	0.278
Zr8	A02 tip	1.5	212	718	0.30	0.0984	0.0492	0.01383	0.00061	0.002049	0.000041	0.46	13.293	0.268
Zr9	A03 tip	0.7	112	312	0.36	0.1201	0.0600	0.01413	0.00083	0.002082	0.000046	0.37	13.503	0.296
Zr10	A04 tip	1.0	294	459	0.64	0.2134	0.1067	0.01526	0.00076	0.002032	0.000043	0.42	13.171	0.278
Zr16	A08 tip	1.9	594	763	0.78	0.2597	0.1298	0.01683	0.00063	0.002044	0.000040	0.53	13.244	0.262
Zr17	A09 centre	1.3	217	596	0.36	0.1215	0.0607	0.01356	0.00059	0.002055	0.000041	0.46	13.329	0.267
Zr18	A10 tip	3.1	280	1554	0.18	0.0602	0.0301	0.01322	0.00064	0.002040	0.000042	0.42	13.240	0.271
Zr19	B01 tip	0.6	193	253	0.76	0.2546	0.1273	0.01294	0.00091	0.002024	0.000047	0.33	13.115	0.304
Zr20	B02 tip	1.3	657	475	1.38	0.4611	0.2305	0.01266	0.00065	0.002034	0.000042	0.40	13.157	0.274
Zr21	B03 tip	0.5	126	181	0.69	0.2315	0.1158	0.02020	0.00145	0.002076	0.000054	0.36	13.452	0.346
Zr22	B05 tip	1.0	421	404	1.04	0.3468	0.1734	0.01283	0.00069	0.002019	0.000043	0.39	13.073	0.276
Zr26	B07 tip	0.7	165	298	0.55	0.1840	0.0920	0.01341	0.00101	0.002046	0.000049	0.32	13.265	0.319
Zr27	B07 tip	1.4	446	619	0.72	0.2398	0.1199	0.01379	0.00063	0.002007	0.000041	0.44	13.008	0.264
Zr28	B08 tip	1.0	295	429	0.69	0.2289	0.1144	0.01328	0.00070	0.002054	0.000043	0.40	13.311	0.279
Zr30	C01 centre	0.9	253	395	0.64	0.2134	0.1067	0.01318	0.00073	0.002051	0.000044	0.38	13.294	0.282
Zr35	C04 tip	1.5	306	694	0.44	0.1470	0.0735	0.01278	0.00062	0.002031	0.000041	0.42	13.172	0.268
Zr37	C05 tip	2.0	163	884	0.18	0.0613	0.0307	0.02283	0.00076	0.002121	0.000042	0.59	~~13.760~~	~~0.268~~
Zr39	C06 tip	3.2	225	1652	0.14	0.0454	0.0227	0.01319	0.00045	0.002019	0.000038	0.56	13.106	0.248
Zr40	C07 tip	1.2	238	503	0.47	0.1579	0.0790	0.01420	0.00068	0.002278	0.000046	0.42	~~14.760~~	~~0.299~~
Zr45	C09 tip	2.1	253	1072	0.24	0.0786	0.0393	0.01277	0.00051	0.002022	0.000039	0.48	13.122	0.255
Zr46	C10 tip	2.3	747	1003	0.75	0.2484	0.1242	0.01226	0.00061	0.002028	0.000041	0.41	13.142	0.267
Zr47	D01 tip	0.6	308	208	1.49	0.4955	0.2477	0.01209	0.00103	0.002033	0.000050	0.29	13.147	0.327
Zr48	D03 tip	1.2	593	430	1.38	0.4600	0.2300	0.01379	0.00072	0.002043	0.000043	0.40	13.215	0.277
Zr7 160421a	D04 side	0.5	153	194	0.79	0.2632	0.1316	0.01291	0.00116	0.002026	0.000053	0.29	13.127	0.341
Zr8	D05 tip	0.6	210	261	0.81	0.2686	0.1343	0.01513	0.00092	0.002027	0.000046	0.37	13.133	0.296
Zr9	D06 centre	0.7	104	356	0.29	0.0971	0.0485	0.01445	0.00085	0.002031	0.000045	0.38	13.178	0.290
Zr10	D07 side	3.8	1350	1567	0.86	0.2872	0.1436	0.01280	0.00041	0.002033	0.000039	0.60	13.170	0.250
Zr11	D08 centre	1.6	606	663	0.91	0.3047	0.1523	0.01303	0.00074	0.002022	0.000043	0.38	13.097	0.281
Zr12	D09 tip	1.2	174	513	0.34	0.1132	0.0566	0.01498	0.00071	0.002383	0.000049	0.43	~~15.440~~	~~0.313~~
Zr15	D10 tip	2.6	863	911	0.95	0.3159	0.1579	0.01527	0.00061	0.002323	0.000046	0.49	~~15.032~~	~~0.295~~
Zr16	D11 centre	1.9	733	749	0.98	0.3260	0.1630	0.01374	0.00056	0.002031	0.000040	0.48	13.153	0.259
Zr17	E01 tip	2.2	1022	786	1.30	0.4336	0.2168	0.01836	0.00074	0.002054	0.000041	0.50	13.289	0.269
Zr18	E02 tip	0.9	143	326	0.44	0.1465	0.0733	0.01623	0.00089	0.002411	0.000051	0.39	~~15.616~~	~~0.331~~
Zr20	E05 tip	0.7	161	303	0.53	0.1768	0.0884	0.01249	0.00095	0.002060	0.000049	0.31	13.355	0.315
Zr21	E06 tip	2.3	1363	758	1.80	0.5995	0.2998	0.01314	0.00057	0.002060	0.000041	0.46	13.310	0.266
Zr22	E07 centre	0.4	92	180	0.51	0.1701	0.0850	0.01268	0.00108	0.002037	0.000051	0.29	13.208	0.328
Zr26	E09 tip	1.4	522	576	0.91	0.3023	0.1512	0.01378	0.00068	0.002027	0.000042	0.41	13.130	0.269
Zr27	E10 tip	0.9	214	381	0.56	0.1876	0.0938	0.01323	0.00077	0.002030	0.000044	0.37	13.161	0.282

GJ-1

Analysis_#	Pb207/U235	2 sigma error Pb207/U235	Pb206/U238	2 sigma error Pb206/U238	Rho
Zr1 150421a	0.8076	0.0174	0.09742	0.00180	0.86
Zr2	0.8102	0.0173	0.09706	0.00178	0.86
Zr3	0.8060	0.0169	0.09756	0.00179	0.88
Zr4	0.8091	0.0172	0.09765	0.00180	0.87
Zr13	0.8117	0.0167	0.09761	0.00179	0.89
Zr14	0.8070	0.0167	0.09769	0.00179	0.89
Zr23	0.8024	0.0165	0.09762	0.00178	0.88
Zr24	0.8103	0.0168	0.09769	0.00178	0.88
Zr33	0.8114	0.0169	0.09710	0.00176	0.87
Zr34	0.8101	0.0170	0.09762	0.00177	0.87
Zr43	0.8104	0.0171	0.09762	0.00177	0.86
Zr44	0.8120	0.0174	0.09679	0.00176	0.85
Zr51	0.8042	0.0175	0.09771	0.00177	0.83
Zr52	0.8066	0.0173	0.09775	0.00176	0.84
Zr1 160421a	0.8054	0.0171	0.09756	0.00180	0.87
Zr2	0.8116	0.0172	0.09743	0.00180	0.87
Zr3	0.8033	0.0171	0.09764	0.00180	0.87
Zr4	0.8118	0.0170	0.09765	0.00180	0.88
Zr13	0.8084	0.0169	0.09750	0.00178	0.87
Zr14	0.8110	0.0171	0.09720	0.00177	0.87
Zr23	0.8090	0.0174	0.09731	0.00176	0.84
Zr24	0.8088	0.0173	0.09740	0.00176	0.85
Zr31	0.8097	0.0177	0.09757	0.00176	0.82
Zr32	0.8054	0.0178	0.09777	0.00176	0.81

91500

Analysis_#	Pb207/U235	2 sigma error Pb207/U235	Pb206/U238	2 sigma error Pb206/U238	Rho
Zr5 150421a	1.8376	0.0393	0.1796	0.0033	0.86

Zr21	0.00264	0.00003	0.00073	0.00002
Zr22	0.00128	0.00002	0.00035	0.00001
Zr23	0.00067	0.00089	0.00827	0.00045
Zr24	0.00068	0.00089	0.0084	0.00046
Zr25	0.00363	0.00004	0.00108	0.00002
Zr26	0.00185	0.00002	0.00051	0.00001
Zr27	0.00119	0.00002	0.00032	0.00001
Zr28	0.00129	0.00002	0.00035	0.00001
Zr29	0.0028	0.00004	0.00083	0.00002
Zr30	0.00134	0.00002	0.00037	0.00001
Zr31	0.0045	0.00005	0.00124	0.00003
Zr32	0.00176	0.00003	0.0005	0.00001
Zr33	0.00069	0.00088	0.00844	0.00045
Zr34	0.00069	0.00089	0.0085	0.00048
Zr35	0.00115	0.00002	0.00031	0.00001
Zr36	0.00108	0.00017	0.00255	0.00055
Zr37	0.00138	0.00002	0.00038	0.00002
Zr38	0.00083	0.00002	0.00022	0.00001
Zr39	0.00085	0.00002	0.00022	0.00001
Zr40	0.00113	0.00002	0.00034	0.00001
Zr41	0.00453	0.00004	0.00129	0.00003
Zr42	0.00426	0.00005	0.00111	0.00005
Zr43	0.00069	0.00088	0.00855	0.00048
Zr44	0.00071	0.00088	0.00868	0.0005
Zr45	0.00096	0.00002	0.00026	0.00001
Zr46	0.00112	0.00002	0.0003	0.00001
Zr47	0.00188	0.00003	0.00051	0.00001
Zr48	0.00133	0.00002	0.00036	0.00001
Zr49	0.00089	0.00163	0.02019	0.00072
Zr50	0.00088	0.00164	0.01991	0.00072
Zr51	0.00071	0.00089	0.00875	0.00052
Zr52	0.0007	0.00088	0.00865	0.0005

GLITTER!: Age estimates (ma).

Analysis_#	Pb207/Pb20(	Pb206/U238	Pb207/U235	Pb208/Th232
Zr1	607.5	599.3	601.1	610.9
Zr2	622.6	597.2	602.6	601.8
Zr3	600.4	600.1	600.2	601.1
Zr4	606.6	600.6	602	588.5
Zr5	1046.6	1064.9	1059	1066.9
Zr6	1070.9	1064	1066.4	1055.7
Zr7	252	13.1	14.5	13.5
Zr8	145.6	13.2	13.9	13.8
Zr9	158.9	13.4	14.3	14.2
Zr10	389.6	13.1	15.4	13.5
Zr11	160.6	13.6	14.4	14.8
Zr12	0.1	13.5	12.3	13
Zr13	614.4	600.4	603.4	604.6
Zr14	600.2	600.8	600.8	585
Zr15	0.1	13.4	12.3	14.2
Zr16	593.2	13.2	16.9	15.5
Zr17	91.1	13.2	13.7	13.2
Zr18	48.6	13.1	13.3	14.3
Zr19	16.5	13	13.1	13.1
Zr20	0.1	13.1	12.8	13.1
Zr21	944.4	13.4	20.3	16.2
Zr22	0.1	13	12.9	12.9
Zr23	589.5	600.4	598.2	604.4
Zr24	609	600.9	602.6	612.8
Zr25	0.1	14.2	14.1	14.6
Zr26	74.4	13.2	13.5	12.8
Zr27	186.5	12.9	13.9	13.8
Zr28	43.1	13.2	13.4	13.4
Zr29	0.1	14.1	9.3	13.3
Zr30	28.9	13.2	13.3	13.2
Zr31	7.9	13.2	13.2	13.2
Zr32	0.1	13.6	13	14.9
Zr33	624.9	597.4	603.2	591.2
Zr34	610	600.4	602.5	613.9
Zr35	0.1	13.1	12.9	12.8
Zr36	1142.4	118	183.7	890.6
Zr37	1148.4	13.7	22.9	29.3
Zr38	0.1	13	12.4	13.5
Zr39	67.8	13	13.3	13.6
Zr40	0.1	14.7	14.3	14.8
Zr41	235.4	13.6	15	16.7
Zr42	0.1	12.3	7.3	33.3
Zr43	610.7	600.4	602.7	594.6
Zr44	633.6	595.6	603.6	600.2
Zr45	0.1	13	12.9	13.2
Zr46	0.1	13.1	12.4	13
Zr47	0.1	13.1	12.2	13.6
Zr48	146	13.2	13.9	13
Zr49	1052.5	1064.6	1060.7	1048.2
Zr50	1034.1	1067.9	1057	1053.5
Zr51	592.8	601	599.2	603.4
Zr52	597.8	601.2	600.5	597.3

Zr6	1.8584	0.0392	0.1795	0.0033	0.87
Zr49	1.8423	0.0404	0.1796	0.0033	0.83
Zr50	1.8319	0.0398	0.1802	0.0033	0.84
Zr5 160421a	1.8419	0.0395	0.1797	0.0033	0.86
Zr6	1.8610	0.0397	0.1796	0.0033	0.86
Zr29	1.8550	0.0416	0.1799	0.0033	0.81
Zr30	1.8684	0.0420	0.1795	0.0033	0.81

GLITTER!: Age estimates: 1 sigma uncertainty (ma).

Analysis_#	Pb207/Pb20	Pb206/U238	Pb207/U235	Pb208/Th232
Zr1	24.93	5.28	4.89	9.77
Zr2	24.67	5.26	4.85	9.46
Zr3	24.3	5.27	4.74	8.95
Zr4	24.59	5.28	4.82	9.14
Zr5	23.14	9.07	7.04	11.68
Zr6	22.79	9.04	6.97	11.48
Zr7	60.75	0.14	0.37	0.19
Zr8	52.91	0.13	0.31	0.23
Zr9	69.71	0.15	0.42	0.28
Zr10	56.89	0.14	0.38	0.2
Zr11	93.29	0.17	0.57	0.44
Zr12	0	0.17	0.5	0.23
Zr13	23.99	5.24	4.68	8.66
Zr14	24.11	5.25	4.68	8.54
Zr15	0	0.17	0.52	0.36
Zr16	41.61	0.13	0.32	0.2
Zr17	53.72	0.13	0.3	0.21
Zr18	59.3	0.13	0.32	0.3
Zr19	84.99	0.15	0.46	0.23
Zr20	14.17	0.14	0.33	0.17
Zr21	74.72	0.17	0.72	0.32
Zr22	66.21	0.14	0.35	0.18
Zr23	24.19	5.22	4.66	8.77
Zr24	24.23	5.23	4.71	9.02
Zr25	173.99	0.27	1.08	0.5
Zr26	90.7	0.16	0.51	0.27
Zr27	54.46	0.13	0.32	0.2
Zr28	64.54	0.14	0.35	0.21
Zr29	0	0.25	0.84	0.42
Zr30	66.35	0.14	0.37	0.21
Zr31	219.13	0.29	1.25	0.59
Zr32	10.38	0.16	0.5	0.3
Zr33	24.32	5.18	4.73	8.92
Zr34	24.57	5.21	4.77	9.41
Zr35	36.69	0.13	0.31	0.21
Zr36	27.26	1.09	2.16	10.54
Zr37	34.83	0.13	0.38	0.45
Zr38	0	0.12	0.22	0.24
Zr39	42.52	0.12	0.22	0.24
Zr40	15.81	0.15	0.34	0.24
Zr41	193.09	0.28	1.29	0.7
Zr42	0	0.3	1.12	0.97
Zr43	24.75	5.2	4.8	9.36
Zr44	24.96	5.16	4.87	9.73
Zr45	36.07	0.13	0.26	0.23
Zr46	0	0.13	0.3	0.2
Zr47	0	0.16	0.52	0.23
Zr48	62.37	0.14	0.36	0.19
Zr49	23.92	8.93	7.21	13.8
Zr50	23.51	8.94	7.14	13.88
Zr51	24.98	5.2	4.93	10.25
Zr52	25.25	5.19	4.86	9.82

GLITTER!: Mean Raw CPS background subtracted.

Analysis_#	Pb204	Pb206	Pb207	Pb208	Th232	U238
Zr1	3	101121	6032	1109	32920	946017
Zr2	35	96935	5823	1055	31775	909834
Zr3	29	98767	5873	1057	31853	921912
Zr4	4	103181	6154	1100	33862	961783
Zr5	18	71071	5234	7635	127971	360001
Zr6	24	72110	5375	7754	131315	365430
Zr7	83	4594	233	1593	2157610	2057763
Zr8	0	6802	330	780	1038732	3017061
Zr9	60	3432	167	485	625928	1497543
Zr10	20	4932	266	1203	1636994	2203818
Zr11	2	3751	183	360	445520	1615122
Zr12	61	1922	80	684	966175	829197
Zr13	0	115403	6910	1258	37518	1071970
Zr14	0	116237	6915	1204	37115	1078426
Zr15	20	1709	72	256	329374	743345
Zr16	0	8263	489	2818	3309704	3661409
Zr17	0	6498	308	872	1210377	2862513
Zr18	0	16815	784	1220	1561742	7458641
Zr19	0	2661	122	751	1044958	1189188
Zr20	0	5021	225	2546	3551320	2231590
Zr21	69	1954	136	602	679765	850678
Zr22	26	4243	194	1611	2272334	1898199
Zr23	11	112643	6671	1209	35901	1041799
Zr24	28	115010	6873	1247	36509	1062411
Zr25	20	873	39	233	290032	358047
Zr26	32	3180	150	626	889941	1401573
Zr27	28	6478	320	1828	2407447	2908867
Zr28	11	4599	214	1178	1593602	2017123
Zr29	8	554	16	174	238247	227922
Zr30	20	4116	190	977	1348679	1806689
Zr31	0	567	26	137	188736	249004

Analysis_#	**Pb CPS**	**Th CPS**	**U CPS**	**Pb CPS moy**	**Th CPS moy**	**U CPS moy**	**Pb ppm**	**Th ppm**	**U ppm**	**Th/U**
Zr1	**108265**	**32920**	**1076454**				***20.2***	***6.7***	***224***	***0.03***
Zr2	**103848**	**31775**	**1035282**				***20.2***	***6.7***	***224***	***0.03***
Zr3	**105726**	**31853**	**1049025**	***108083***	***32858***	***1071709***	***20.2***	***6.7***	***224***	***0.03***
Zr4	**110439**	**33862**	**1094394**	***108083***	***32858***	***1071709***	***20.2***	***6.7***	***224***	***0.03***
Zr5	83958	127971	409638				16	26	86	0.30
Zr6	85263	131315	415815				16	27	87	0.31
Zr7	6503	2157610	2341487				1	440	489	0.90
Zr8	7912	1038732	3433053				1	212	718	0.30
Zr9	4144	625928	1704024				1	112	312	0.36
Zr10	6421	1636994	2507680				1	294	459	0.64
Zr11	4296	445520	1837815				1	80	336	0.24
Zr12	2747	966175	943527				0	173	173	1.00
Zr13	**123571**	**37518**	**1219773**	***123964***	***37317***	***1223446***	***20.2***	***6.7***	***224***	***0.03***
Zr14	**124356**	**37115**	**1227119**	***123964***	***37317***	***1223446***	***20.2***	***6.7***	***224***	***0.03***
Zr15	2057	329374	845837				0	59	155	0.38
Zr16	11570	3309704	4166244				2	594	763	0.78
Zr17	7678	1210377	3257196				1	217	596	0.36
Zr18	18819	1561742	8487038				3	280	1554	0.18
Zr19	3534	1044958	1353153				1	193	253	0.76
Zr20	7792	3551320	2539282				1	657	475	1.38
Zr21	2761	679765	967969				0	126	181	0.69
Zr22	6074	2272334	2159923				1	421	404	1.04
Zr23	**120534**	**35901**	**1185442**	***121846***	***36205***	***1197169***	***20.2***	***6.7***	***224***	***0.03***
Zr24	**123158**	**36509**	**1208896**	***121846***	***36205***	***1197169***	***20.2***	***6.7***	***224***	***0.03***
Zr25	1165	290032	407415				0	54	76	0.70
Zr26	3988	889941	1594822				1	165	298	0.55
Zr27	8654	2407447	3309942				1	446	619	0.72
Zr28	6002	1593602	2295244				1	295	429	0.69
Zr29	752	238247	259348				0	45	50	0.90
Zr30	5303	1348679	2055795				1	253	395	0.64
Zr31	730	188736	283337				0	35	54	0.65

Zr32	0	3762	166	888	1078480	1607820	Zr32	4816	1078480	1829506				1	202	351	0.58
Zr33	0	113921	6861	1219	36863	1054724	Zr33	**122001**	**36863**	**1200149**	***118912***	***35747***	***1166184***	***20.2***	***6.7***	***224***	***0.03***
Zr34	69	108096	6466	1191	34631	995024	Zr34	**115822**	**34631**	**1132218**	***118912***	***35747***	***1166184***	***20.2***	***6.7***	***224***	***0.03***
Zr35	49	7177	325	1154	1631786	3173545	Zr35	8705	1631786	3611113				1	306	694	0.44
Zr36	7	24477	1893	16113	320401	1189308	Zr36	42490	320401	1353290				7	60	260	0.23
Zr37	0	9557	741	1408	867906	4044536	Zr37	11706	867906	4602197				2	163	884	0.18
Zr38	26	14075	614	800	1071786	6235504	Zr38	15515	1071786	7095255				3	201	1363	0.15
Zr39	0	17103	805	922	1223195	7595587	Zr39	18830	1223195	8642867				3	225	1652	0.14
Zr40	39	5876	264	1065	1296550	2312498	Zr40	7244	1296550	2631345				1	238	503	0.47
Zr41	15	363	18	91	98859	153750	Zr41	487	98859	174949				0	18	33	0.54
Zr42	0	267	7	218	118154	125636	Zr42	492	118154	142959				0	22	27	0.79
Zr43	0	111147	6653	1205	36040	1019194	Zr43	**119005**	**36040**	**1159720**	***119784***	***36440***	***1171569***	***20.2***	***6.7***	***224***	***0.03***
Zr44	11	112502	6806	1243	36840	1040019	Zr44	**120562**	**36840**	**1183417**	***119784***	***36440***	***1171569***	***20.2***	***6.7***	***224***	***0.03***
Zr45	1	11139	507	1006	1374000	4926788	Zr45	12653	1374000	5606094				2	253	1072	0.24
Zr46	0	10453	455	2942	4064234	4608844	Zr46	13850	4064234	5244311				2	747	1003	0.75
Zr47	8	2170	93	1270	1677854	953970	Zr47	3541	1677854	1085503				1	308	208	1.49
Zr48	35	4418	215	2259	3116978	1932284	Zr48	6927	3116978	2198707				1	593	430	1.38
Zr49	0	71186	5268	7581	126846	353974	Zr49	84035	126846	402780				14	24	79	0.31
Zr50	21	72215	5296	7810	129936	357724	Zr50	85342	129936	407047				15	25	80	0.31
Zr51	45	106933	6348	1157	33983	976294	Zr51	**114483**	**33983**	**1110905**	***118184***	***35224***	***1146395***	***20.2***	***6.7***	***224***	***0.03***
Zr52	25	113854	6777	1229	36464	1038672	Zr52	**121885**	**36464**	**1181884**	***118184***	***35224***	***1146395***	***20.2***	***6.7***	***224***	***0.03***
