# Supplementary material for: A 13.06 Ma widespread ignimbrite in the Pannonian Basin captured a snapshot of shallow marine to coastal environment in Central Paratethys
Source: Sci Rep. 2025 Jul 2;15:23528. doi: 10.1038/s41598-025-07002-9 (PMC12223212; doi:10.1038/s41598-025-07002-9)
Supplement: Supplementary file 10 — Supplementary Information 8B. [file 41598_2025_7002_MOESM10_ESM.pdf]

Supplement 8 B

age for ____ : Age ± internal Ar/Ar uncertainty // full external error source uncertainty

age for ___ : Age ± for comparison to other Ar/Ar result // for comparison to other method result

age for TSZM-Dobi site: 13.046 ± 0.065 // 0.091

age for Lénárddaróc site: 13.066 ± 0.038 // 0.073

age for Lénárddaróc with sanidine only: 13.065 ± 0.033 // 0.073

age for both sites with plagioclase grains only: 13.060±0.033 // 0.071

age for both sites, all grains together, i.e. the **eruption age**: 13.064 ± 0.019 // 0.065

$^{40}Ar/^{39}Ar$ result

$^{40}Ar/^{39}Ar$ dating result are presented as probability diagrams in Fig. 6. Details of measurements and results are detailed in Supplement 8B. All errors in this study are reported at 2σ. Weighted mean age uncertainties are reported, including J uncertainty and were calculated using Isoplot 4.0 (Ludwig, 2012). Measurements spreading of the TSZM-Dobi and Lénarddaróc inverse isochrone are around 40% and 60%, and the resulting $^{40}Ar/^{36}Ar$ initial intercepts is within uncertainty of that of the atmosphere (296.9±4.7 and 300.1±2.9, respectively), suggesting that the dated crystals do not contain trapped excess argon.

For the TSZM-Dobi site, the 11 crystal measurements yield a weighted mean age of 13.046±0.065 Ma (0.091 Ma including all uncertainties, MSWD=2.31, p=0.021) with an age spectrum ranging between 12.91±0.17 and 13.22±0.18 Ma. The probability spectrum is flattened by the spreading of individual $^{40}Ar/^{39}Ar$ ages and their individual uncertainties.

For the Lénárddaróc site, the age spectrum is more tightened, individual $^{40}Ar/^{39}Ar$ ages ranging between 13.03±0.06 and 13.16±0.23 Ma. It has benefitted to the presence of 3 sanidine grains among dated grains, the uncertainties of which, 5 times lower than on plagioclase grains, is significantly improving the age uncertainty. This allowed the calculation of a robust and precise weighted mean age of 13.066±0.038 Ma (0.073 Ma including all uncertainties, MSWD=0.84, p=0.58). A reasonable probability of fit coincides with a roughly Gaussian distribution centred at 13.069 Ma (Fig. X). Note age obtained from the three sanidine grains only and from the 7 plagioclase grains are indistinguishable (13.069±0.031 and 13.065±0.033 Ma, respectively), supporting the idea of a cogenetic crystallization of the two population.

As TSZM-Dobi and Lénárddaróc weighted mean ages are compatible, and considering that such huge eruptions are quite rare, it supports the hypothesis of a single eruption sampled and dated twice. To test this hypothesis, all grains results were merged together. Inverse isochrone yields a resulting $^{40}Ar/^{36}Ar$ initial intercept of 297.9±1.9 (MSWD=1.7) within uncertainty compatible with that of the atmosphere. The 21 grains yield a weighted mean age of 13.064 ± 0.019 Ma (0.065 Ma including all external errors; MSWD=1.4, p=0.25). This result is highly affected by the well-constrained ages from sanidine grains. Considering all grains but sanidine ones (18 grains) yields a weighted mean age of 13.060±0.033 Ma (MSWD=1.6, p=0.065) that remains indistinguishable from the one of sanidines, supporting this absence of inherited argon in plagioclase grains. The absence of measured sanidine in

TSZM-Dobi sample is explained by the aim to privilege the biggest crystals in order to assure a minimal amount of released gas, which should result in missing the very small and rare sanidine in it. Furthermore, the possibility of a polyphased eruption with heterogeneity in the crystal content of each pulse, possibly due to a stratified magmatic chamber, may have also led to the rarity of sanidine in the TSZM-Dobi site.

Average analytical errors, which express the amount of contamination affecting a grain population, is four times larger for the TSZM-Dobi than the Lénárddaróc site (±0.056 and ±0.012, respectively), in accordance with the spread of dispersion of individual age. We applied the alteration index (AI) defined by Baksi (2007), which is a measure of the extent of secondary alteration based on ^{36}Ar and ^{39}Ar contents, to better constrain our data and check how the final age was affected from altered feldspars. Plagioclases are considered unaltered if their AI value is lower than 0.00006, or relative AI (i.e. RAI = AI/000006) lower than 1. For the Lénárddaróc site, all but 2 grains have relative AI values lower than the cutoff value. The 2 other grains, having ages the most different to the mean value with the worst uncertainties, display RAI values of about 1.8 and 25, supporting a global good quality for most of the grains. For the TSZM-Dobi site, RAI values are significantly higher ranging between 1.3 and 12.5 times for the permitted threshold value. The RAI values, larger for TSZM-Dobi grains, are consistent with the average individual ^{40}Ar/^{39}Ar ages' uncertainty that are 50% higher for the TSZM-Dobi plagioclase than the Lénárddaróc ones (± 0.080 and ± 0.054, respectively). Splitting the TSZM-Dobi grains in two populations, RAI<4 and RAI>4, yield slightly different ages (13.08±0.12 and 13.01±0.10, respectively, with analytical error only), both compatible together and with Lénnárddaróc age, and the one provided by the lower RAI value being very close to these of Lénárddaróc site. This age values supports the hypothesis of a slight loss of argon in the more altered grains but in a proportion that is not outside of the uncertainty ranges. The state of grains from the TSZM-Dobi site much more altered than those from Lénárddaróc, deduced from the alteration index computed thanks to isotopic argon measurements, is also supported by other weathering indices that all provided average values larger on TSZM-Dobi samples (Supplementary Material table, see below).

From these data, considering that both sites give compatible ages, we retain the age values provided by the 21 grains, 13.064±0.019 Ma for ^{40}Ar/^{39}Ar age comparison and 13.064±0.065 Ma, including all external errors, for comparison to other methods, as the most probable eruption age.

Table: Main weathering indexes, computed for samples obtained at TSZM-Dobi and Lénárddaróc pyroclastic samples, respectively. All but WIP display values that highlight the higher degree of weathering at TSZM-Dobi compared to Lénárddaróc site.

weathering indexes	**L.O.I.**	**WIP** Parker (1970)	**V** Roaldset (1972)	**P.I.A.** Fedo et al. (1995)	**C.I.A.** Nesbitt & Young (1982)	**CIW** Harnois (1988)
TSZM-Dobi	4.95 ± 0.59	68.6 ± 2.94	7.97 ± 1.47	69.21 ± 1.42	57.03 ± 0.71	83.43 ± 2.24
Lénárdd.	4.24 ± 0.6	62.07 ± 2.94	5.21 ± 1.37	67.23 ± 2.88	58.59 ± 1.03	78.05 ± 2.83

Cox, S. E., Hemming, S. R. & Tootell, D. The Isotopx NGX and ATONA Faraday amplifiers. Geochronology 2, 231–243. https://doi. org/ 10. 5194/ gchron- 2- 231- 2020 (2020).

Ludwig K.R., 2021, User's Manual for Isoplot Version 3.75–4.15: a Geochronological Toolkit for Microsoft Excel, Berkeley Geochronological Center Special Publication, 5

Niespolo, E.M., Rutte, D., Deino, A., et Renne, P.R., 2017. Intercalibration and Age of the Alder Creek Sanidine 40Ar/39Ar Standard. Quaternary Geochronology in press. https://doi.org/10.1016/j.quageo.2016.09.004.

Renne, P.R., Mundil, L.R., Balco, G., Min, K., et Ludwig, K.R., 2011. Joint determination of 40K decay constants and 40Ar*/40K for the Fish Canyon sanidine standard, and improved accuracy for 40Ar/39Ar geochronology. Response to the comment by W.H. Schwarz et al. Geochem. Cosmochim. Acta 75, 5097e5100.
