# Supplementary material for: A 13.06 Ma widespread ignimbrite in the Pannonian Basin captured a snapshot of shallow marine to coastal environment in Central Paratethys
Source: Sci Rep. 2025 Jul 2;15:23528. doi: 10.1038/s41598-025-07002-9 (PMC12223212; doi:10.1038/s41598-025-07002-9)
Supplement: Supplementary file 11 — Supplementary Information 9. [file 41598_2025_7002_MOESM11_ESM.pdf]

Table 1. 1/1

	Locality	Lon[˚]<br>Lat[˚]	n/no	D [˚]	I [˚]	k	α_{95}[˚]	Dc [˚]	Ic [˚]	k	α_{95}[˚]	dip, f	Paleo lat	Pole lat	Pole long	dp	dm
1	Tarnaszentmária, Dobi-oldal H547-559	47.8814<br>20.2108	12/13	337	+39	37	7	357	+50	37	7	112/23, f=0.54	31	73	209	6	10
2	Lénárddaróc 6292-300	48.1472<br>20.3604	8/9	353	+46	58	7	353	+46	58	7	f=0.54	28	69	218	6	9
3	Egercsehi 9093-103	48.0425<br>20.2683	10/11	346	+47	319	3	346	+47	319	3	f=0.54	28	67	233	2	4
4	Sajószentpéter 9174-181	48.2101<br>20.7153	8/8	343	+56	88	8	343	+56	88	8	f=0.54	36	73	253	8	11
5	Edelény, Csisztapuszta 9182-187	48.3239<br>20.7075	4/6	348	+57.9	104	9	348	+53	104	9	f=0.54	39	77	248	10	13

Key: Lat.N, Lon.E: Geographic coordinates (WGS84) measured by GPS (Garmin GPSmap 60CSx), n/no: number of used/collected samples (the samples are independently oriented cores D, I (Dc, Ic): declination, inclination before (after) tilt correction; k and α95: statistical parameters (Fisher, 1953); Lat and Lon: coordinates of the paleomagnetic pole;δp and δm: half cones of the error ellipse of the paleomagnetic pole.
