# Supplementary material for: A 13.06 Ma widespread ignimbrite in the Pannonian Basin captured a snapshot of shallow marine to coastal environment in Central Paratethys
Source: Sci Rep. 2025 Jul 2;15:23528. doi: 10.1038/s41598-025-07002-9 (PMC12223212; doi:10.1038/s41598-025-07002-9)
Supplement: Supplementary file 12 — Supplementary Information 10. [file 41598_2025_7002_MOESM12_ESM.pdf]

Possible source vents as seen on Bouguer anomaly map (greenish);
magnetic ΔZ anomalies (deep blue to reddish) are also shown

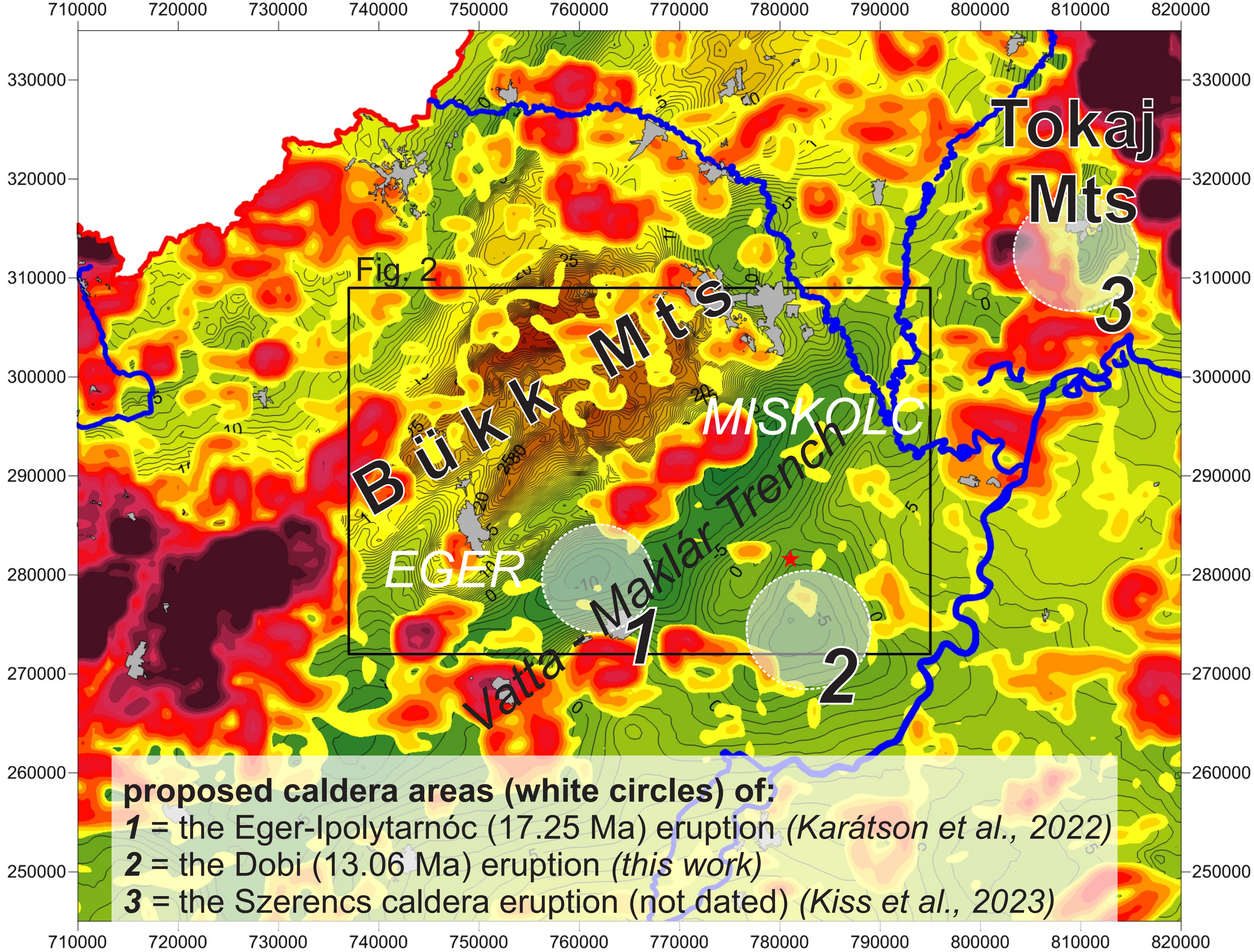

References:

Karátson et al. (2022): Karátson, D., Biró, T., Portnyagin, M., Kiss, B., Paquette, J.-L., Cseri, Z., Hencz, M., Németh, K., Lahitte, P., Márton, E., Kordos, L., Józsa, S., Hably, L., Müller, S., Szarvas, I. Large-magnitude (VEI ≥ 7) 'wet' explosive silicic eruption preserved a lower Miocene habitat at the Ipolytarnóc Fossil Site, North Hungary. Sci. Rep. 12, 9743 (2022).

Kiss et al. (2023): Kiss, J., Cserkész-Nagy, Á., Lőrincz, K., Rádi, K.:The interpretation of the deep geological construction of Nyírség - local volcanic morphology based on geophysical measurements. Magyar Geofizika, 64 (3), 131-155 (2023)
